# The CtrCBL1/CtrCIPK6 Complex of Citrus Phosphorylates CtrBBX32 to Regulate CtrSTP1‐Mediated Sugar Accumulation and Cold Tolerance

**DOI:** 10.1002/advs.202508372

**Published:** 2025-09-26

**Authors:** Xiangming Shang, Zeqi Zhao, Wei Xiao, Yike Zeng, Mengdi Li, Xin Jiang, Bachar Dahro, Lele Chu, Min Wang, Chunlong Li, Ji‐Hong Liu

**Affiliations:** ^1^ National Key Laboratory for Germplasm Innovation and Utilization of Horticultural Crops College of Horticulture and Forestry Science Huazhong Agricultural University Wuhan 430070 China; ^2^ Hubei Hongshan Laboratory Wuhan 430070 China; ^3^ College of Life Sciences Gannan Normal University Ganzhou 341000 China

**Keywords:** citrus trifoliata, cold stress, protein kinase, sugar transport protein, transcriptional regulation

## Abstract

Sugar accumulation is a crucial adaptive strategy for plant cold tolerance, yet its regulatory mechanisms in the roots under cold conditions remain largely unclear. Here, a cold‐inducible sugar transporter protein (*CtrSTP1*), which is highly expressed in the roots of trifoliate orange (*Citrus trifoliata* L.), is identified. CtrSTP1 functions positively in cold tolerance by facilitating sugar accumulation in the roots. Two transcription factors, CtrBBX32 and CtrZAT10, bind to the *CtrSTP1* promoter, with CtrBBX32 acting as transcriptional repressor and CtrZAT10 as activator. CtrBBX32 also suppresses *CtrZAT10* expression. Accordingly, CtrBBX32 and CtrZAT10 act as negative and positive regulators, respectively, of cold tolerance by modulating *CtrSTP1* expression. Moreover, CtrCBL1 and CtrCIPK6 form a complex to phosphorylate CtrBBX32 and promote its protein degradation, leading to relief of CtrBBX32‐mediated repression on *CtrZAT10* and *CtrSTP1*. Further transgenic evidence confirms that CtrCIPK6 plays a positive role in cold tolerance through modulating the sugar pathway. Taken together, these findings uncover a novel regulatory module composed of CtrCBL1/CtrCIPK6–CtrBBX32 that controls cold‐induced sugar accumulation by directly or indirectly (via CtrZAT10) modulating the expression of the root‐localized *CtrSTP1*. The work provides valuable knowledge to advance the understanding of sugar transport and homeostasis in the roots when plants are exposed to adverse environments.

## Graphical abstract

The CtrCBL1‐CtrCIPK6 module of trifoliate orange (*Citrus trifoliata* L.) phosphorylates CtrBBX32, triggering its protein degradation under cold conditions, thereby relieving CtrBBX32‐mediated transcriptional repression of *CtrZAT10* and *CtrSTP1*. The consequent upregulation of CtrZAT10 further activates *CtrSTP1* expression, which enhances hexose transport into cells and maintains sugar homeostasis, ultimately leading to improved cold tolerance.

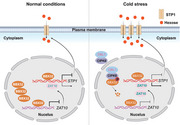

## 1 Introduction

Temperature stands as one of the most critical environmental factors governing plant growth and development, with low temperature posing significant constraints on crop productivity and geographic distribution.^[^ ^1^, ^2^ ^]^ As sessile organisms, plants cannot evade cold stress through physical relocation but have evolved intricate molecular regulatory networks to orchestrate physiological and molecular adaptations. These responses encompass transcriptional reprogramming, translational adjustments, and metabolic remodeling, which collectively modulate the abundance of stress‐related proteins, metabolites, and phytohormones.^[^ ^3^, ^4^ ^]^ A hallmark of cold adaptation is the intracellular accumulation of osmoprotectants, a strategy critical for preserving membrane integrity and mitigating cellular dehydration under low‐temperature conditions. Key compounds in this process include betaine, proline, and soluble sugars.^[^ ^5^, ^6^, ^7^, ^8^ ^]^ While soluble sugars are predominantly synthesized in photosynthetic source tissues (e.g., leaves), their subsequent allocation to sink organs via source‐to‐sink translocation mechanisms represents a vital component of systemic cold tolerance.^[^ ^9^ ^]^ Nevertheless, the molecular mechanisms governing sugar transport and utilization in sink tissues, particularly roots, during cold stress remain incompletely characterized.

The spatial distribution of carbohydrates in plants is tightly regulated by diverse sugar transporters localized across tissues and cellular compartments. Multiple classes of membrane‐bound sugar transporters have been identified in plants, including sugars will eventually be exported transporters (SWEETs), sucrose transporters (SUTs), and monosaccharide transporters (MSTs).^[^ ^10^ ^]^ These transporters play pivotal roles in orchestrating plant responses to both biotic and abiotic stresses through carbon source distribution or sugar‐mediated signaling.^[^ ^11^ ^]^ Notably, sugar transport proteins (STPs) within the MST family primarily mediate hexose uptake from the apoplast. Current studies of STPs are predominantly reported in rhizospheric sugar acquisition from the soil,^[^ ^12^, ^13^ ^]^ competition with pathogens for apoplastic sugars or the import of soluble sugars into the apoplast that may be utilized by pathogens,^[^ ^14^, ^15^ ^]^ and sugar utilization and storage processes.^[^ ^16^, ^17^ ^]^ These multifaceted roles underscore the critical involvement of STPs in balancing nutrient acquisition, pathogen interaction dynamics, and metabolic regulation within plant systems. While cold stress has been demonstrated to induce the expression of *STP* members,^[^ ^18^, ^19^, ^20^ ^]^ the mechanistic underpinnings of STP‐mediated cold tolerance, particularly their regulatory networks and upstream effectors, remain inadequately characterized.

The expression of genes is precisely regulated by diverse transcription factors (TFs), enabling organisms to adapt to various environmental stimuli. Among these regulators, ZAT proteins, members of the cysteine‐2/histidine‐2 (C2H2)‐type zinc finger family, have been documented to participate in salt, drought, and cold stress responses.^[^ ^21^, ^22^ ^]^ Similarly, BBX proteins, which belong to the B‐Box zinc finger family, play critical roles in plant adaptation to abiotic stresses.^[^ ^23^, ^24^ ^]^ Despite the gradual elucidation of the roles of BBX and ZAT family transcription factors under a variety of environmental cues, including cold stress, further investigation is required to understand how these factors influence plant cold responses and their potential involvement in the accumulation of soluble sugars under cold stress.

Under transient cold stress, plant cells exhibit a rapid elevation in cytosolic Ca^2^⁺ levels due to Ca^2^⁺ influx from the apoplast or vacuole,^[^ ^25^ ^]^ which is subsequently recognized by calcium sensor proteins.^[^ ^26^ ^]^ A suite of calcium‐decoding proteins, including calmodulin (CaM), calmodulin‐like proteins, calcium‐dependent protein kinases, and calcineurin B‐like proteins (CBLs), have been identified as critical components in the calcium signaling response to cold stress.^[^ ^27^, ^28^, ^29^, ^30^, ^31^ ^]^ Notably, the CBL/CIPK signaling network plays a pivotal role in plant responses to cold stress.^[^ ^32^ ^]^ Furthermore, their involvement in sugar transport processes under stress conditions has been reported through multiple studies. For instance, in apple (*Malus domestica*), MdCIPK22 was shown to phosphorylate MdSUT2.2, promoting sugar accumulation and drought tolerance;^[^ ^33^ ^]^ while under salt stress, MdCIPK13 enhanced the stability and transport activity of MdSUT2.2 through phosphorylation.^[^ ^34^ ^]^ In cotton (*Gossypium hirsutum*), the CBL2–CIPK6–TST2 signaling cascade regulated glucose homeostasis to improve abiotic stress resilience.^[^ ^35^ ^]^ In rice (*Oryza sativa*), transgenic plants overexpressing *OsCIPK03* and *OsCIPK12* exhibited significantly higher soluble sugar content compared to wild‐type plants under cold and drought stresses, likely attributed to the markedly elevated expression levels of sugar transporter genes.^[^ ^36^ ^]^ However, the mechanistic understanding of how CBL/CIPK modules regulate sugar transport specifically under low‐temperature signaling remains to be elucidated.

In this study, we identified an STP‐encoding gene, *CtrSTP1*, which exhibited high expression levels in the roots and was upregulated under cold conditions in trifoliate orange (*C. trifoliata* L.), a cold‐hardy plant widely used as citrus rootstock. We further elucidated its positive role in enhancing soluble sugar accumulation in roots and conferring plant cold resistance. Additionally, the regulatory mechanisms underlying cold‐induced *CtrSTP1* expression involving CtrBBX32, CtrZAT10, and calcium signal sensor complex CtrCBL1/CIPK6 were investigated. These regulators modulate plant cold tolerance by controlling CtrSTP1‐mediated soluble sugar accumulation. Collectively, we established a regulatory module composed of CtrCBL1/CtrCIPK6–CtrBBX32–CtrZAT10 that fine‐tunes *CtrSTP1* expression to modulate root sugar homeostasis, thereby enhancing cold tolerance in citrus.

## 2 Results

### 2.1 *CtrSTP1* was Cold‐Inducible and Exhibited Abundant Expression in the Roots

**Figure 1:**
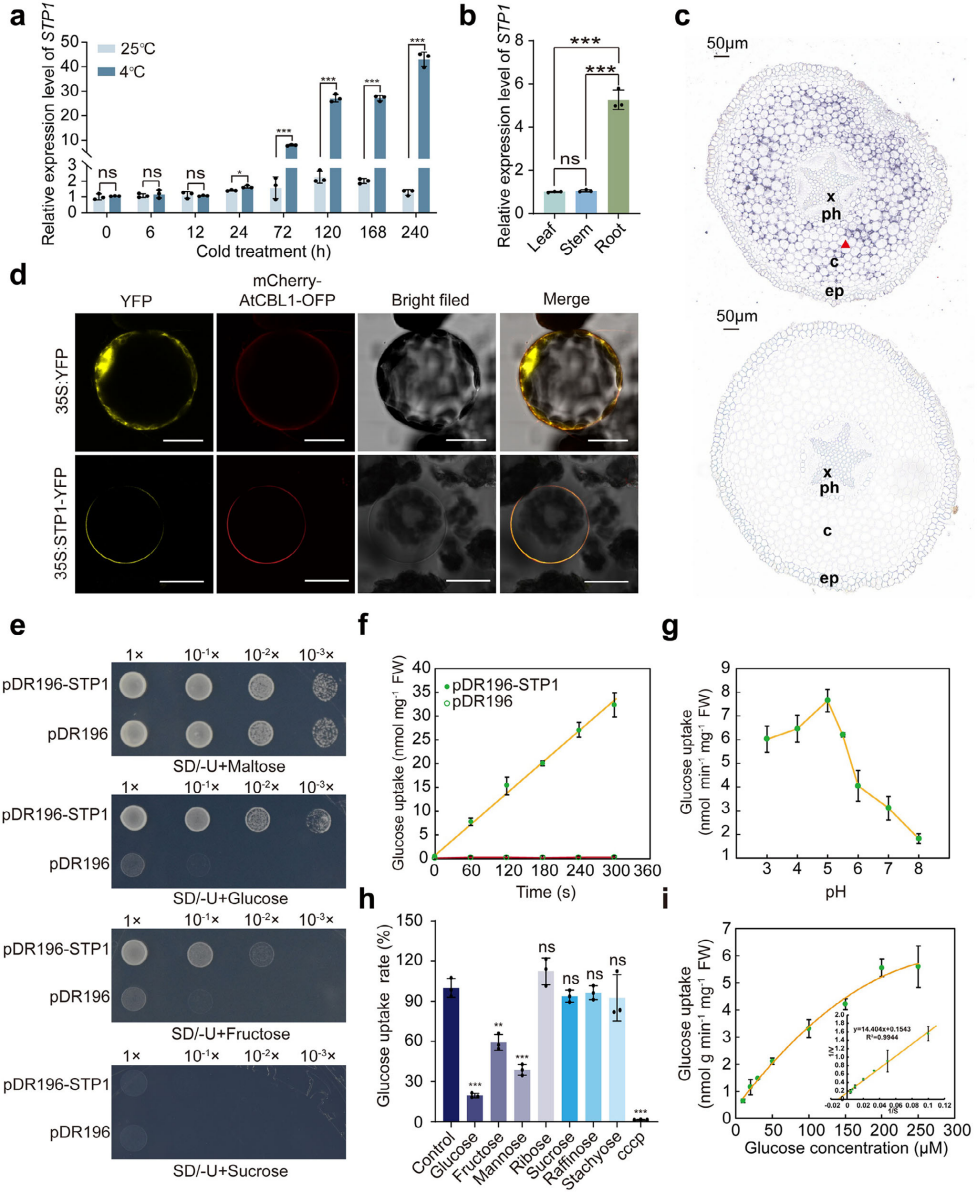
CtrSTP1 is a plasma membrane‐localized monosaccharide transporter and responds to cold stress. a) The relative expression of *CtrSTP1* in trifoliate orange plants under normal growth conditions (25 °C) or cold treatment (4 °C). The expression level of *CtrSTP1* at 0 h under 25 °C was set to 1.0. b) The relative expression of *CtrSTP1* in different tissues, including leaves, roots, and stems. The expression level of *CtrSTP1* in the leaves was set to 1.0. c) Cell‐type‐specific in situ detection of *CtrSTP1* mRNA within transverse sections of trifoliate orange root tips. The upper and lower panels show the images after hybridization with the digoxigenin‐labeled *CtrSTP1* antisense probe and sense probe, respectively. ep, epidermis; c, cortex; ph, phloem; x, xylem. Scale bars: 50 µm. d) The subcellular localization of CtrSTP1‐YFP in leaf protoplasts of *N. benthamiana*. AtCBL1‐OFP works as a plasma membrane marker. Scales bar: 25 µm. e) Growth of the sugar transporter‐deficient yeast (CSY4000) cells transformed with pDR196‐CtrSTP1 or pDR196 on synthetic dropout (SD)/‐U (Ura) medium added with maltose, glucose, fructose or sucrose. f) Time–course changes in the uptake rate of ^14^C‐glucose by CSY4000 cells transformed with pDR196‐*CtrSTP1* or pDR196. g) Relative uptake rates of ^14^C‐glucose by CSY4000 cells expressing *CtrSTP1* at different pH. h) Uptake rates of ^14^C‐glucose (100 µm) in the CSY4000 cells expressing *CtrSTP1* in medium containing different competitive sugars (1 mm) or CCCP (50 µm), a metabolic inhibitor. The competitive sugars and CCCP were added 60 s before adding ^14^C‐glucose. The rate without any competitor was used as the internal control. i) Uptake rates of ^14^C‐glucose in the CSY4000 cells expressing *CtrSTP1* in the medium containing glucose at different concentrations. The Eadie–Hofstee transform is performed on the data used to estimate *Km*. Error bars denote ± standard deviation (SD, *n* = 3). Two‐tailed Student^’^s *t*‐test was conducted for analyzing the significant difference (*P < 0.05, **P < 0.01, ***P < 0.001; *P* > 0.05, ns, no significance).

To explore the potential role of sugar transporters in modulation of soluble sugar accumulation in citrus under cold stress, we analyzed the expression patterns of sugar transporter genes based on the previous transcriptome dataset generated by RNA‐seq from cold‐treated trifoliate orange plants.^[^ ^37^ ^]^ One of the *STPs*, named *CtrSTP1* (*Pt2g026610*), was found to be dramatically induced by cold treatment (Figure S1a,b, Supporting Information). RT‐qPCR also showed that transcript levels of *CtrSTP1* began to increase in the roots at 24 h under cold conditions (4 °C), and then surged sharply from 72 h to the highest level at 240 h, whereas the mRNA abundance remained low and stable at normal temperature (25 °C) (**Figure** **1**a). In addition, *CtrSTP1* was prominently expressed in the root, relative to the leaf and stem (Figure S1c, Supporting Information), which was corroborated by RT‐qPCR (Figure 1b). And in situ hybridization assays showed that *CtrSTP1* was mainly expressed in cortical parenchyma cells of roots (Figure 1c). Subcellular localization analysis using tobacco protoplasts demonstrated that yellow fluorescent protein (YFP) signal was distributed in the both cytoplasm and nucleus when the control vector (35S:YFP) was used. By contrast, the YFP signal was only observed in the plasma membrane, precisely overlapping with the membrane marker, when 35S:CtrSTP1‐YFP vector was transformed. These results indicate that CtrSTP1 is a plasma membrane‐localized protein (Figure 1d). Given that *CtrSTP1* expression was upregulated under cold conditions and predominantly expressed in the roots, we measured the contents of soluble sugars, including fructose, glucose, and sucrose, in the roots of trifoliate orange exposed to cold treatment. The results indicated that the soluble sugars in the roots gradually increased in the presence of cold treatment (Figure S2, Supporting Information), which were positively correlated with the expression levels of *CtrSTP1*, implying the potential role of *CtrSTP1* in mediating soluble sugar accumulation in the roots under cold stress.

The sugar transport characteristics of CtrSTP1 was examined using a sugar transporter deficient yeast strain CSY4000, which cannot take up hexose or sucrose.^[^ ^38^ ^]^ To this end, CSY4000 transformed with a fusion construct pDR196‐*CtrSTP1* or the empty vector pDR196 (used as a negative control) was plated on the synthetic dropout (SD)/‐Ura (U) media supplemented with different sugars. The yeast cells grew well on medium containing maltose, but not on the medium added with sucrose. By contrast, the yeast cells transformed with pDR196‐*CtrSTP1* could grow normally on the media supplemented with glucose or fructose as the sole carbon source, whereas the growth of negative control was repressed (Figure 1e), implying that CtrSTP1 could uptake both glucose and fructose. To further determine the kinetic parameters of CtrSTP1, we performed sugar uptake assays using ^14^C‐glucose (^14^C‐Glc). As expected, the yeast cells transformed with pDR196‐*CtrSTP1* absorbed ^14^C‐Glc, which was not taken up by the negative (Figure 1f). In addition, the highest transport rate of ^14^C‐Glc for the yeast cells transformed with pDR196‐*CtrSTP1* was detected when they were cultured in the medium with a pH of 5 (Figure 1g). We then determined the substrate specificity of CtrSTP1 by measuring the transport rates of ^14^C‐Glc in the presence of tenfold excess nonradioactive sugars. The uptake rate of ^14^C‐Glc was significantly reduced by adding the hexoses, including glucose, fructose and mannose, but not by either pentose (ribose) or other oligosaccharides (sucrose, raffinose, stachyose), indicating that CtrSTP1 uses hexose as its specific substrate (Figure 1h). Additionally, the proton uncoupler carbonyl cyanide m‐chlorophenyl hydrazine (CCCP) significantly inhibited the uptake of ^14^C‐Glc by the yeast cells transformed with pDR196‐CtrSTP1 (Figure 1h), suggesting that CtrSTP1‐mediated hexose uptake required a proton gradient. According to the uptake of ^14^C‐Glc supplied at different concentrations, the *Km* of CtrSTP1 for glucose was 100.63 ± 23.92 µm, and the maximum uptake rate (*V*_max_) was 6.85 ± 1.22 pmol mg^−1^ min^−1^ cells at pH 5.0 (Figure 1i). Taken together, these results indicate that CtrSTP1 is a cold‐induced hexose transporter localized in the plasma membrane.

### 2.2 *CtrSTP1* Functions in Cold Tolerance by Affecting the Soluble Sugar Content

**Figure 2:**
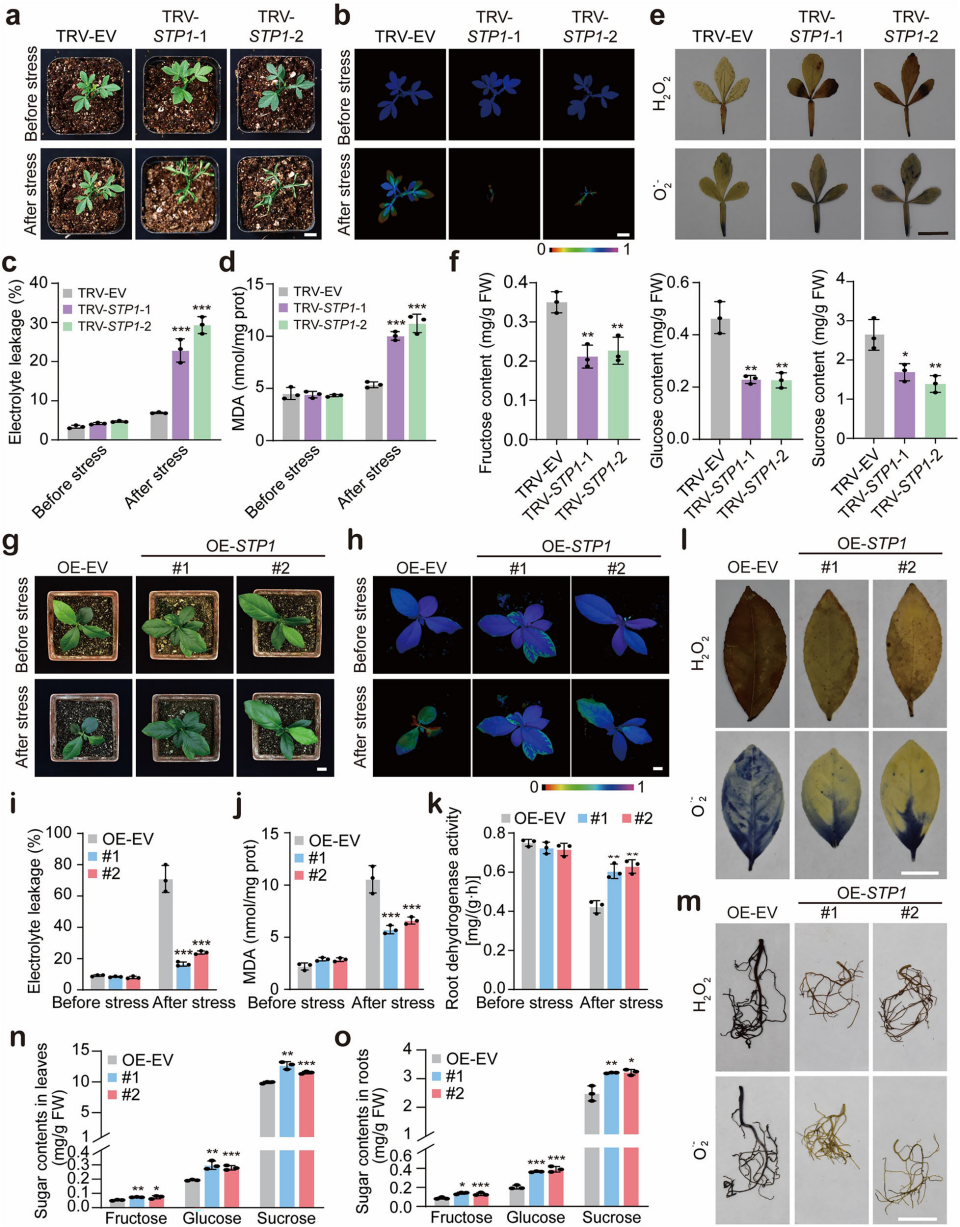
Functional analysis of *CtrSTP1* in cold tolerance. a) Phenotype of representative plants from the virus‐induced gene silencing (VIGS) lines (TRV‐*CtrSTP1*‐1, TRV‐*CtrSTP1*‐2) and the TRV‐EV control before cold treatment (before stress) and after exposure to −6 °C for 6 h, followed by growth recovery at room temperature for 12 h (after stress). Three independent plants were used for each line. Scale bars: 2 cm. b–d) Chlorophyll fluorescence imaging (b), electrolyte leakage (c), and MDA content (d) of the tested plants before and after the cold treatment. Scale bar: 2 cm. e) In situ detection of H_2_O_2_ and O_2_^•−^ by histochemical staining with 3,3‐diaminobenzidine (DAB) (upper panels) and nitro blue tetrazolium (NBT) (lower panels) in the leaves of the tested plants after the cold treatment (Scale bar: 2 cm). f) The contents of glucose, fructose, and sucrose in the roots of the test lines. g) Plant phenotypes of the OE lines (OE‐*CtrSTP1*) and the OE‐EV (control plant) before cold treatment (before stress) and after exposure to −2 °C for 4 h, followed by growth recovery at room temperature for 12 h (after stress). Scale bars: 2 cm. h–k) Chlorophyll fluorescence imaging (Scale bar = 2 cm) (h), EL (i), MDA content (j), and root dehydrogenase activity (k) of the tested lines before and after the cold treatment. l,m) In situ detection of H_2_O_2_ and O_2_^•−^ by histochemical staining with DAB (upper panels) and NBT (lower panels) in the leaves (l) and roots (m) of the tested plants after the cold treatment (Scale bar: 2 cm). n,o) The contents of glucose, fructose, and sucrose in the leaves (n) and roots (o) of the test lines. Error bars denote ± standard deviation (SD, *n* = 3). Two‐tailed Student^’^s *t*‐test was conducted for analyzing the significant difference (*P < 0.05, **P < 0.01, ***P < 0.001).

As *CtrSTP1* was induced by cold stress, efforts were made to investigate the biological function of *CtrSTP1* in cold tolerance. To this end, tobacco rattle virus (TRV)‐based virus‐induced gene silencing (VIGS) approach was employed to knock down *CtrSTP1* in trifoliate orange (Figure S3a, Supporting Information). No morphological variation was observed in the VIGS plants relative to the TRV‐EV (empty vector) control under normal growth conditions. When exposed to cold treatment (−6 °C for 6 h), the VIGS lines exhibited quicker and more conspicuous plant damages, as evidenced by severe leaf curling and withering, compared with the TRV‐EV control (**Figure** **2**a). Congruent with the plant phenotype, the VIGS plants showed substantially dampened chlorophyll fluorescence (Figure 2b), higher electrolyte leakage (EL) and Malondialdehyde (MDA) levels (Figure 2c,d), greater accumulation of reactive oxygen species (ROS), including H_2_O_2_ and O_2_^.−^ (Figure 2e), relative to the TRV‐EV control under cold treatment. In addition, the contents of soluble sugars, including both hexose (fructose and glucose) and sucrose, in the roots of the VIGS plants were significantly lower than those in the TRV‐EV control (Figure 2f). To clarify the changing of sucrose content, we further analyzed transcript levels of sucrose phosphate synthase (SPS) genes (*CtrSPS1*/*CtrSPS2*/*CtrSPS3*/*CtrSPS4*), which are the key rate‐limiting enzymes regulating sucrose synthesis. The expression of *CtrSPS2*/*CtrSPS3*/*CtrSPS4* in the VIGS lines was lower than that in the TRV‐EV control (Figure S3b, Supporting Information), implying that lower fructose and glucose content led to a decrease in the expression level of *CtrSPS2*/*CtrSPS3*/*CtrSPS4*, which in turn led to a decrease in the synthetic sucrose. The photosynthetic rate of the VIGS lines and the TRV‐EV control was also measured, which showed that there was no dramatic difference in the photosynthetic rate (Figure S3c, Supporting Information), indicating that the decrease of sugar content in the VIGS lines was caused by sugar transport and metabolism.

Next, *Agrobacterium*‐mediated genetic transformation was utilized to generate transgenic lemon (*Citrus × limon*) plants overexpressing the *CtrSTP1* gene, a citrus species known for its sensitivity to cold. Two independent transgenic lines (#1 and #2) were selected for subsequent cold tolerance evaluation (Figure S3d, Supporting Information). Following exposure to freezing conditions (−2 °C for 4 h), the control plants (OE‐EV) exhibited severe leaf desiccation and wilting, whereas the transgenic lines overexpressing *CtrSTP1* (OE‐*CtrSTP1*) maintained viability with minimal leaf damage (Figure 2g). Under standard growth conditions, no significant differences were observed in chlorophyll fluorescence parameters, EL, MDA content, or root dehydrogenase activity between OE‐*CtrSTP1* and OE‐EV plants. However, under cold stress conditions, the OE lines demonstrated significantly enhanced chlorophyll fluorescence, reduced EL and MDA accumulation, higher root dehydrogenase activity, and lower ROS accumulation in both leaves and roots compared to the control plants (Figure 4h–m). Additionally, the contents of fructose, glucose, and sucrose in the leaves and roots of the OE‐*CtrSTP1* lines were significantly higher than those of the OE‐EV control (Figure 4n,o). These findings collectively demonstrate that *CtrSTP1* overexpression promotes soluble sugar accumulation and confers improved cold stress tolerance in lemon plants.

To further explore the function of *CtrSTP1* in roots, we generated hairy roots overexpressing *CtrSTP1* (OE‐*CtrSTP1*) via *Agrobacterium rhizogenes*‐mediated transformation of trifoliate orange shoots with a vector containing *CtrSTP1* fused in‐frame with a green fluorescent protein (GFP) gene, using hairy roots expressing the empty vector as a negative control (OE‐EV).^[^ ^39^ ^]^ GFP signal was detected in all of the positive hairy roots, but absent in the shoots (Figure S4a, Supporting Information). RT‐qPCR showed that the expression levels of *CtrSTP1* in the OE‐*CtrSTP1* lines were significantly higher than that of OE‐EV (Figure S4a, Supporting Information). When subjected to the cold treatment (−6 °C for 12 h), the OE‐EV was damaged to a worsened extent, as manifested by severe leaf curling and withering, dramatically impaired chlorophyll fluorescence (Figure S4b,c, Supporting Information), significantly higher EL and MDA levels (Figure S4d,e, Supporting Information), lower root dehydrogenase activity (Figure S4f, Supporting Information), and greater ROS accumulation in the roots (Figure S4g, Supporting Information), in comparison with the OE‐*CtrSTP1* plants. In addition, the contents of fructose, glucose, and sucrose in the roots of the OE‐*CtrSTP1* lines were significantly higher than those of the OE‐EV control, as well as the transcript levels of *CtrSPS2*/*CtrSPS3*/*CtrSPS4* in the roots of the overexpression lines were higher in compare with the OE‐EV control (Figure S4h,i, Supporting Information). In summary, these results indicate that overexpression of *CtrSTP1* confers enhanced cold tolerance by promoting soluble sugar accumulation in roots.

### 2.3 CtrBBX32 Represses and CtrZAT10 Activates *CtrSTP1* Transcription

To understand the molecular regulation of *CtrSTP1*, yeast one‐hybrid (Y1H) screening of a DNA library derived from trifoliate orange seedlings was conducted using the promoter of *CtrSTP1* (p*CtrSTP1*) as bait. A total of 70 positive clones were sequenced (Table S1, Supporting Information), in which *Pt7g004290.1* and *Pt8g003020.1* were annotated as B‐box zinc finger protein 32‐like (*CtrBBX32*) and C2H2‐type zinc finger protein (*CtrZAT10*), respectively. *CtrBBX32* and *CtrZAT10* exhibited relatively higher transcript level in the roots than in the other tissues (Figure S5a, Supporting Information). Under cold stress, the expression levels of both genes in the roots were induced, with *CtrBBX32* exhibiting rapid induction followed by a decline over prolonged exposure (Figure S5b, Supporting Information), whereas *CtrZAT10* displayed slower initial induction and progressively increased expression with extended cold treatment (Figure S5c, Supporting Information). Subcellular localization assays indicated that both CtrBBX32 and CtrZAT10 were confirmed to be localized in the nucleus (Figure S6a, Supporting Information). Furthermore, transcriptional activation analysis indicates that CtrZAT10 showed normal transcriptional activation activity within its C‐terminal protein region, whereas CtrBBX32 had no transcriptional activation activity in the yeast system (Figure S6b,c, Supporting Information).

**Figure 3:**
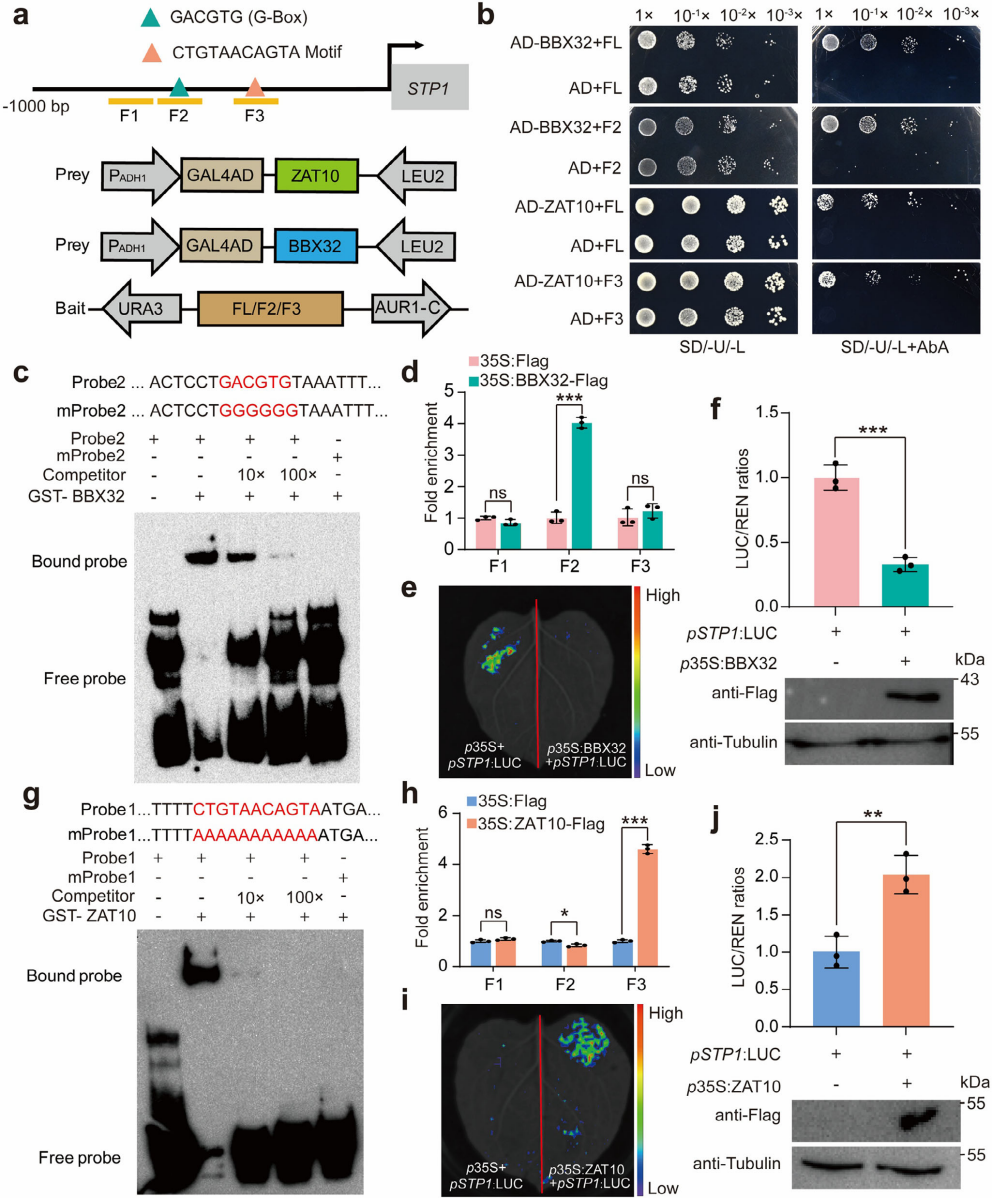
CtrBBX32 and CtrZAT10 act as transcriptional repressor and activator, respectively, of *CtrSTP1*. a) Schematic diagrams of the *CtrSTP1* promoter, the bait, and prey vectors used for yeast one‐hybrid assay. The green and red triangles represent the G‐box element and CTGTAACAGTA motif, responsively. b) Growth of yeast cells transformed with preys (pGADT7‐CtrBBX32 or pGADT7‐CtrZAT10) and different baits as depicted on selective medium without (left) or with (right) AbA (250 ng mL^−1^). Negative control, baits + pGADT7. c) EMSA assays using fusion protein GST‐CtrBBX32 that was incubated with biotin‐labeled original or mutated probes, added with or without competitor DNA. d) ChIP‐qPCR analysis for revealing the enrichment of the promoter regions of *CtrSTP1* with CtrBBX32 protein, using 35S: Flag as a control. e,f) Luciferase imaging (e) and LUC/REN (*Rentilla* LUC) ratios (f) in the leaves infiltrated with the effector, 35S:CtrBBX32 or 35S:Flag, with the LUC reporter driven by the *CtrSTP1* promoter. The protein levels were detected by Western blotting using the antibodies against anti‐Flag and anti‐Actin. g) EMSA assays based on the incubation of the fusion protein GST‐CtrZAT10 with biotin‐labeled probes harboring the original or mutated motif, added with or without competitor DNA. h) ChIP‐qPCR analysis for revealing the enrichment of the promoter regions of *CtrSTP1* with CtrZAT10 protein, using 35S:Flag as a control. i,j) Luciferase imaging (i) and LUC (firefly luciferase)/REN (Renilla luciferase) ratios (j) in the leaves infiltrated with the effector, 35S:CtrZAT10 or 35S:Flag, with the LUC reporter driven by the *CtrSTP1* promoter. The protein levels were detected by Western blotting using the antibodies against anti‐Flag and anti‐Tubulin. Error bars denote ± standard deviation (SD, *n* = 3). Two‐tailed Student^’^s *t*‐test was conducted for analyzing the significant difference (*P < 0.05, **P < 0.01, ***P < 0.001; *P* > 0.05, ns, no significance).

We then used different approaches to assess the interaction between CtrZAT10 and CtrBBX32 with p*STP1*. The 1‐kb p*CtrSTP1* sequence contains a CTGTAACAGTA motif and a G‐box (GACGTG) that are recognized by CtrZAT10 and CtrBBX32 proteins, respectively. First, we constructed the bait vectors using full‐length p*CtrSTP1* or two partial fragments containing either the G‐box elements or CTGTAACAGTA motif, along with the GAL4AD‐CtrBBX32/CtrZAT10 constructs as the preys (**Figure** **3**a). Y1H showed that yeast cells transfected with the prey of CtrBBX32 and the bait containing the G‐box or those transfected with the prey of CtrZAT10 and the bait harboring CTGTAACAGTA core sequence grew well in the synthetic dropout media supplemented with aureobasidin A (AbA) (Figure 3b), implying that CtrBBX32 and CtrZAT10 interact with p*CtrSTP1* through the two *cis*‐acting elements, respectively. To evaluate whether CtrBBX32 and CtrZAT10 directly and specifically interact with their recognizing elements in p*CtrSTP1*, we carried out an electrophoretic mobility shift assay (EMSA) using the recombinant glutathione S‐transferase (GST)‐CtrBBX32 and GST‐CtrZAT10 proteins and biotin‐labeled 40‐bp DNA probes containing the G‐box sequences or the CTGTAACAGTA. An intense shifted band was observed when GST‐CtrBBX32 was incubated with the probe containing G‐box element or when GST‐CtrZAT10 was incubated with the probe containing the CTGTAACAGTA motif. The band shift was reduced by adding the unlabeled competitors in a dose‐dependent manner, whereas the gel shift was completely abolished when the recombinant proteins were incubated with the probe harboring the mutated *cis*‐acting elements (Figure 3c,g). These results demonstrate that CtrBBX32 and CtrZAT10 interact with p*CtrSTP1* by directly and specifically binding to their recognizing motifs. To further assess the interaction between the two TFs and p*CtrSTP1* in vivo, we introduced the constructs of 35S:FLAG (used as a control), 35S:CtrBBX32‐FLAG or 35S:CtrZAT10‐FLAG into citrus callus. Then chromatin immunoprecipitation (ChIP) assay in combination with quantitative PCR (ChIP‐qPCR) using specific primers was performed. As a result, we found that CtrBBX32 and CtrZAT10 were substantially enriched in the promoter regions containing the G‐box motif or the CTGTAACAGTA sequence, respectively (Figure 3d,h), which supported the specific interaction between the two TFs and p*CtrSTP1*. To examine the transcriptional activation ability of CtrBBX32 and CtrZAT10, we carried out dual luciferase (LUC) transient assays in *Nicotiana benthamiana* leaves, using 35S:CtrBBX32 and 35S:CtrZAT10 as the effectors, together with the p*CtrSTP1*:LUC as a reporter. Both fluorescence visualization (Figure 3e,i) and quantitative measurements (Figure 3f,j) indicate that the promoter activity was significantly decreased by 35S:CtrBBX32, but significantly increased by 35S:CtrZAT10, compared with the control. Collectively, our data revealed that both CtrBBX32 and CtrZAT10 directly targeted the *CtrSTP1* promoter, with CtrBBX32 mediating transcriptional repression while CtrZAT10 activated expression.

### 2.4 CtrBBX32 Binds to the Promoter of *CtrZAT10* and Represses its Expression

To delve deeper into the regulatory relationship between CtrBBX32 and CtrZAT10, we performed point‐to‐point verification of the physical interaction and regulatory relationship between them. The yeast two‐hybrid assay results initially indicated that CtrBBX32 and CtrZAT10 do not physically interact with each other (Figure S7a, Supporting Information). Moreover, the bimolecular fluorescence complementation (BiFC) assay also demonstrated that no interaction was detected between the two proteins (Figure S7b, Supporting Information). Based on the promoter sequence analysis, protein–DNA interaction verification using Y1H and dual‐LUC transient assays, we found that CtrZAT10 did not bind to the promoter of *CtrBBX32* (Figure S8, Supporting Information). However, two G‐box *cis*‐acting elements were identified in the promoter of *CtrZAT10* (p*CtrZAT10*) (Figure S9, Supporting Information). Y1H demonstrated that CtrBBX32 bound to one of the two p*CtrZAT10* fragments (Figure S10a,b, Supporting Information). Furthermore, EMSA showed that CtrBBX32 directly and specifically bound to the promoter fragment containing the G‐box sequence (Figure S10c, Supporting Information). ChIP‐qPCR assay indicated that CtrBBX32 was significantly enriched in the promoter region harboring the G‐box element, further verifying the interaction in vivo (Figure S10d, Supporting Information). Dual‐LUC assay revealed that transfection of CtrBBX32 effector and the p*CtrZAT10*:LUC reporter led to a significant repression of the promoter activity (Figure S10e,f, Supporting Information). Collectively, these results demonstrate that CtrBBX32 acts as a transcriptional repressor of *CtrZAT10* by interacting with the G‐box in p*CtrZAT10*.

### 2.5 CtrBBX32 Negatively Regulates Cold Tolerance of Trifoliate Orange

**Figure 4:**
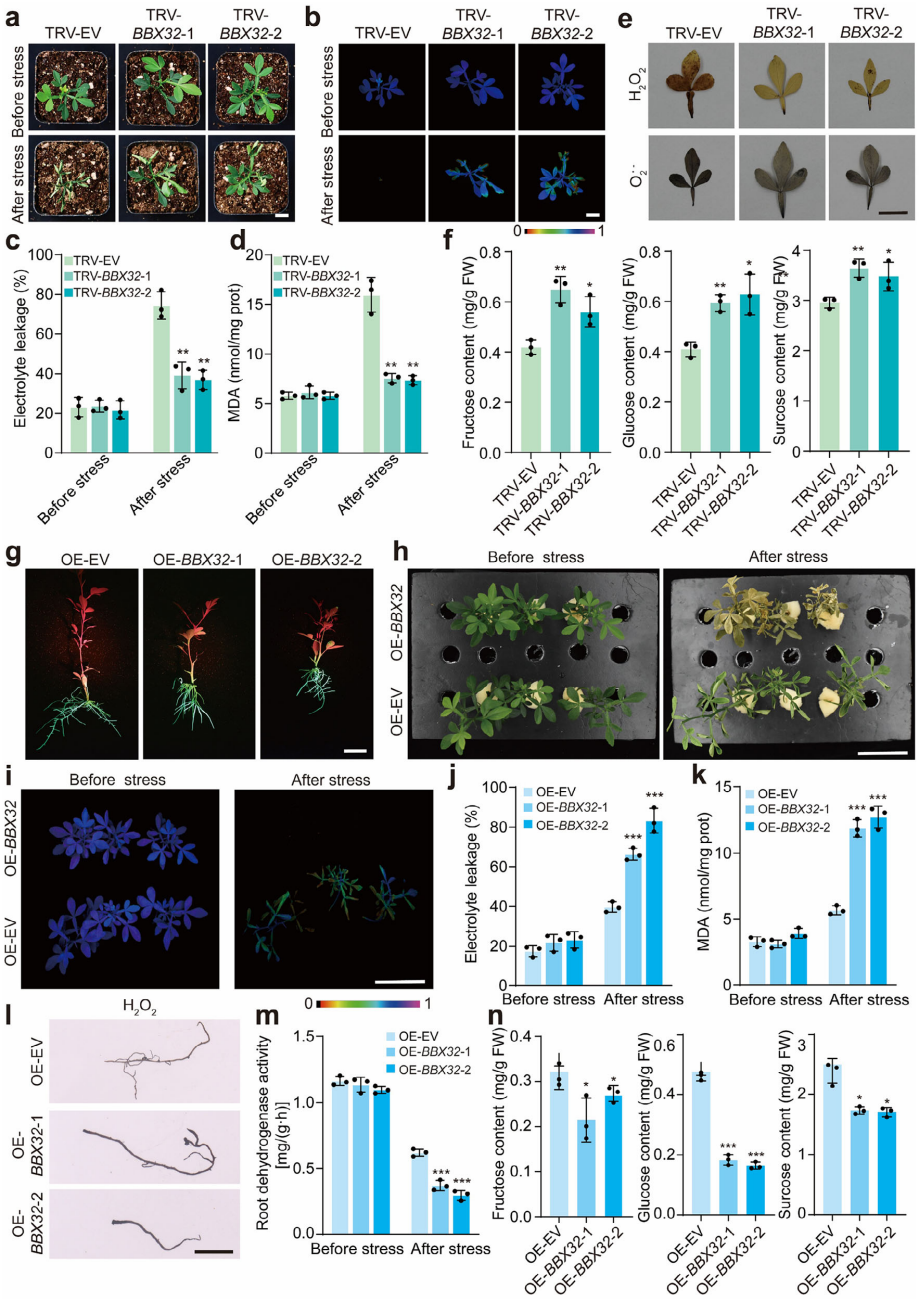
*CtrBBX32* functions negatively in the cold tolerance. a) Phenotypes of representative plants from the virus‐induced gene silencing (VIGS) lines (TRV‐*CtrBBX32*‐1, TRV‐*CtrBBX32*‐2) and the TRV‐EV control before cold treatment (before stress) and after exposure to −6 °C for 6 h, followed by growth recovery at room temperature for 12 h (after stress). Three independent plants were used for each line. Scale bar: 2 cm. b–d) Chlorophyll fluorescence (b), EL (c), and MDA content (d) of the tested lines before and after the cold treatment. Scale bar: 2 cm. e) In situ detection of H_2_O_2_ and O_2_^•−^ by histochemical staining with DAB and NBT, respectively, in the leaves of the tested lines after the cold treatment. Scale bar: 2 cm. f) Contents of fructose, glucose, and sucrose in the tested lines. g) Visualization of green fluorescent protein (GFP) in the trifoliate orange hairy roots derived from *Agrobacterium rhizogenes*‐mediated transformation of 35S:CtrBBX32‐GFP (OE‐*CtrBBX32*) or 35S:GFP empty vector (OE‐EV). The photos shown in the figure labeled OE‐EV, OE‐*CtrBBX32*‐1, and OE‐*CtrBBX32*‐2 are representative plants of each group containing three individual transgenic plants. Scale bar: 2 cm. h) Phenotypes of the plants with hairy roots of OE‐*CtrSTP1* and OE‐EV before cold treatment (before stress) and after exposure to −6 °C for 6 h, and subsequent recovery at room temperature for 12 h (after stress). Scale bar: 5 cm. i–k) Chlorophyll fluorescence imaging (i), EL (j), and MDA content (k) of the tested lines before and after the cold treatment. Scale bar: 5 cm. l) In situ detection of H_2_O_2_ in the hairy roots of the tested lines after the cold treatment. Scale bars: 2 cm. m) Root dehydrogenase activities of the tested lines before and after the cold treatment. n) The contents of fructose, glucose, and sucrose in the hairy roots of the tested lines. Error bars denote ± standard deviation (SD, *n* = 3). Two‐tailed Student^’^s *t*‐test was conducted for analyzing the significant difference (*P < 0.05, **P < 0.01, ***P < 0.001).

To explore the biological function of *CtrBBX32* in the cold tolerance, we knocked down *CtrBBX32* in trifoliate orange by using VIGS. Under normal growth conditions, the silenced plants and the TRV‐EV control were morphologically similar from each other. Phenotypic evaluation was performed by exposing 6‐week‐old seedlings to cold treatment (−6 °C for 6 h). We observed that the TRV‐EV plants exhibited quicker plant damage and drastically decreased cold tolerance relative to the VIGS plants; these phenotypic differences were evidenced by serious leaf curling and necrosis, weakened chlorophyll fluorescence, higher levels of EL, MDA, and ROS in the presence of cold stress (**Figure** **4**a–e). In view of the fact that CtrBBX32 repressed the expression of *CtrSTP1* and *CtrZAT10*, the soluble sugar contents and the expression levels of *CtrSTP1* and *CtrZAT10* were analyzed. The results showed that the contents of fructose, glucose, and sucrose, along with the mRNA abundance of *CtrSTP1* and *CtrZAT10*, in the *CtrBBX32*‐VIGS lines were significantly higher than those in the TRV‐EV control (Figure 4f; Figure S11a, Supporting Information). To further elucidate the role of *CtrBBX32* in cold tolerance, we generated trifoliate orange hairy roots overexpressing *CtrBBX32* by *Agrobacterium rhizogenes*‐mediated transformation. Plants with positive hairy roots (OE‐EV, OE‐*CtrBBX32*) confirmed by examining the expression of GFP (Figure 4g; Figure S11b, Supporting Information) were subjected to cold treatment (−6 °C for 6 h). The OE‐*CtrBBX32* lines were damaged to a greater degree compared with the OE‐EV plants, as indicated by obvious plant death, and collapsed chlorophyll fluorescence (Figure 4h,i). Furthermore, significantly higher EL, MDA, and ROS, but lower root dehydrogenase activity, were noted in the OE‐*CtrBBX32* lines relative to the OE‐EV counterpart (Figure 4j–m). In addition, the contents of fructose, glucose, and sucrose, together with the transcript levels of *CtrSTP1* and *CtrZAT10*, in the roots of the OE‐*CtrBBX32* lines were significantly decreased in comparison with the OE‐EV lines (Figure 4n; Figure S11b, Supporting Information). Taken together, these results show that CtrBBX32 functions as a negative regulator of cold tolerance by downregulating the expression of *CtrSTP1* and *CtrZAT10* and inhibiting the soluble sugar accumulation in the roots.

### 2.6 CtrZAT10 Plays a Positive Role in Cold Tolerance

**Figure 5:**
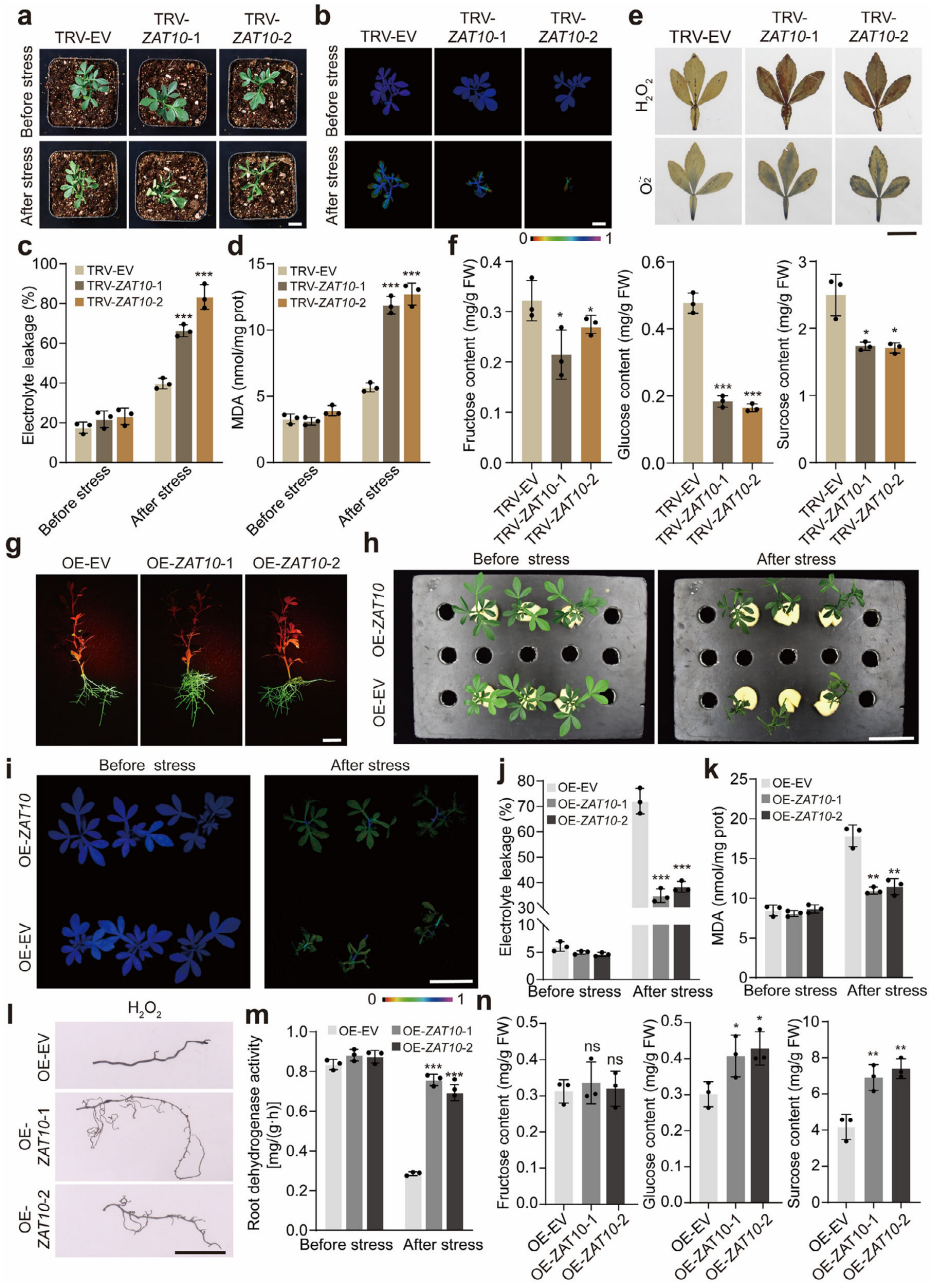
CtrZAT10 plays a positive role in modulation of cold tolerance. a) Phenotypes of plants from the virus‐induced gene silencing (VIGS) lines (TRV‐*CtrZAT10*‐1, TRV‐*CtrZAT10*‐2) and the TRV‐EV control before cold treatment (before stress) and after exposure to −6 °C for 6 h, followed by growth recovery at room temperature for 12 h (after stress). Three independent plants were used for each line. Scale bar: 2 cm. b–d) Chlorophyll fluorescence imaging (b), EL (c), and MDA content (d) of the tested lines before and after the cold treatment. Scale bar: 2 cm. e) In situ detection of H_2_O_2_ and O_2_^•−^ in the leaves of the tested plants after the cold treatment. Scale bar: 2 cm. f) Contents of fructose, glucose, and sucrose in the tested lines. g) Detection of green fluorescent protein (GFP) in the trifoliate orange hairy roots derived from *Agrobacterium rhizogenes*‐mediated transformation of 35S:CtrZAT10‐GFP (OE‐*CtrZAT10)* or 35S:GFP empty vector (OE‐EV). The photos shown in the figure labeled OE‐EV, OE‐*CtrZAT10*‐1, and OE‐*CtrZAT10*‐2 are representative plants of each group containing three individual transgenic plants. Scale bar: 2 cm. h) Phenotypes of the plants with hairy roots of OE‐*CtrZAT10* and OE‐EV before cold treatment (before stress) and after exposure to −4 °C for 4 h, and subsequent recovery at room temperature for 12 h (after stress). Scale bar: 5 cm. i–k) Chlorophyll fluorescence imaging (i), EL (j), and MDA content (k) of the tested lines before and after the cold treatment. Scale bar: 5 cm. l) In situ detection of H_2_O_2_ in the hairy roots of the tested lines after the cold treatment. Scale bar: 2 cm. m) Root dehydrogenase activity of the tested lines before and after the cold treatment. n) The contents of fructose, glucose, and sucrose in the hairy roots of the tested lines. Error bars denote ± standard deviation (SD, *n* = 3). Two‐tailed Student^’^s *t*‐test was conducted for analyzing the significant difference (*P < 0.05, **P < 0.01, ***P < 0.001; *P* > 0.05, ns, no significance).

To illustrate the biological role of *CtrZAT10* in cold tolerance, we first used VIGS to silence *CtrZAT10* in trifoliate orange. Both the TRV‐EV control and the *CtrZAT10‐*VIGS plants grew well under normal conditions. Nevertheless, when 6‐week‐old plants were subjected to cold treatment (−6 °C for 6 h, recovery for 12 h at ambient temperature), the VIGS plants demonstrated prominently decreased cold tolerance compared with the TRV‐EV control, as manifested by quicker and more serious leaf curling and withering and weaker chlorophyll fluorescence (**Figure** **5**a,b). In line with the plant phenotype, higher EL and greater accumulation of MDA and ROS were scored in the VIGS lines relative to the TRV‐EV control in the presence of cold treatment (Figure 5c–e). In addition, the contents of soluble sugars, including fructose, glucose, and sucrose, in the *CtrZAT10‐*VIGS lines were significantly lower than in the TRV‐EV control (Figure 5f). The transcription level of *CtrSTP1* was dramatically downregulated in *CtrZAT10* VIGS lines, while the *CtrBBX32* expression level underwent minor changes (Figure S12a, Supporting Information). Likewise, we employed the *Agrobacterium rhizogenes*‐mediated transformation system to generate hairy roots overexpressing 35S:CtrZAT10‐GFP (OE‐*CtrZAT10*) or 35S:GFP (OE‐EV) in trifoliate orange plants (Figure 5g; Figure S12b, Supporting Information). Upon exposure to cold treatment (−4 °C for 4 h), the OE‐*CtrZAT10* lines exhibited dramatically increased cold tolerance in comparison with the OE‐EV plants, as indicated by better plant growth and stronger chlorophyll fluorescence (Figure 5h,i), lower EL and MDA levels (Figure 5j,k), less ROS in the roots (Figure 5l), but higher root dehydrogenase activity (Figure 5m). Meanwhile, the contents of glucose and sucrose, together with the expression level of *CtrSTP1*, were significantly elevated in the roots of OE‐*CtrZAT10* plants in comparison with OE‐EV, whereas no change in the fructose content was observed (Figure 5n; Figure S12b, Supporting Information). Taken together, these findings suggest that *CtrZAT10* positively regulates cold tolerance of trifoliate orange by upregulating *CtrSTP1* and increasing the contents of soluble sugars.

### 2.7 CtrCIPK6 Interacts with and Phosphorylates CtrBBX32

**Figure 6:**
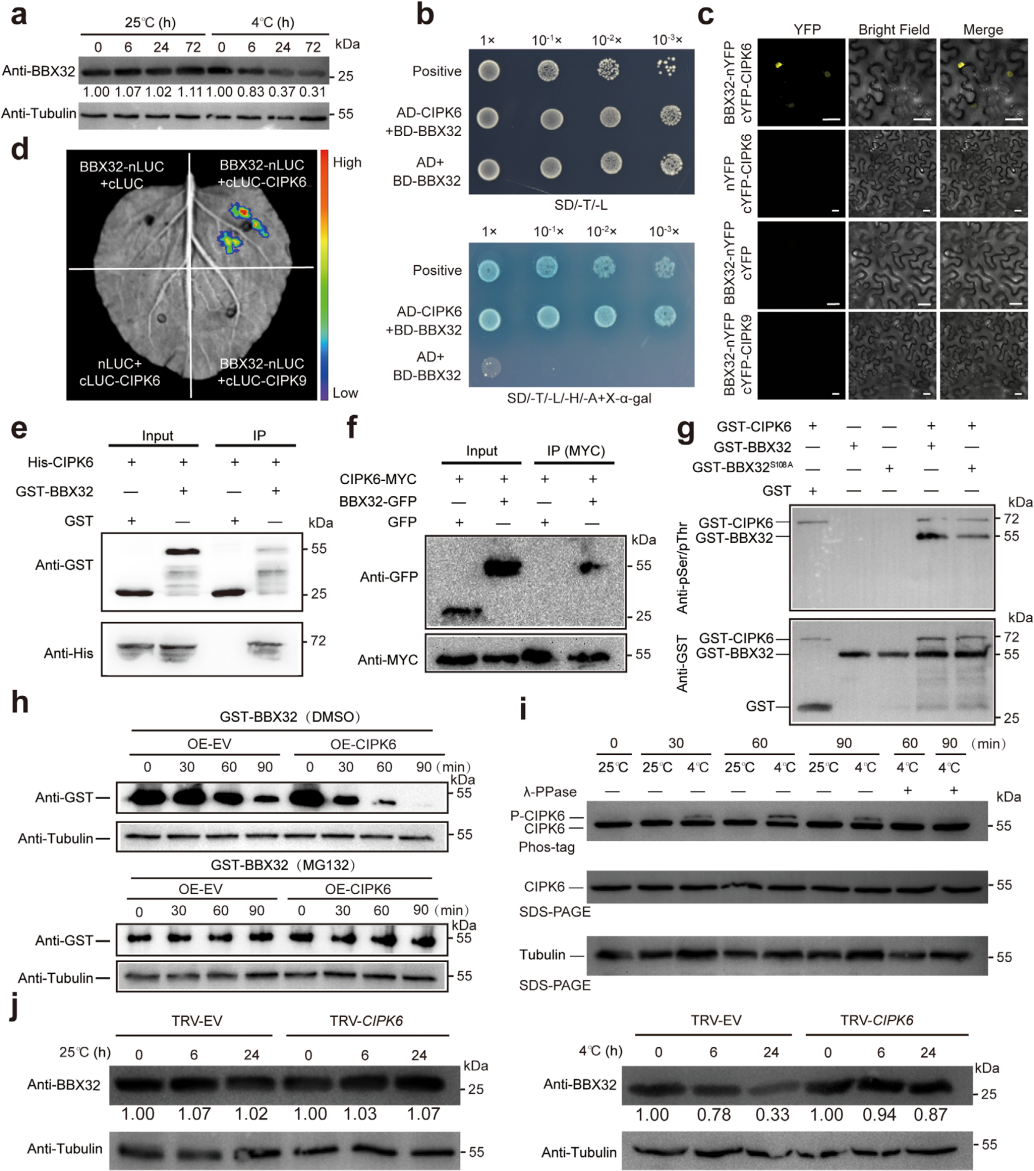
CtrCIPK6 interacts with and phosphorylates CtrBBX32. a) Immunoblotting analyses revealing the CtrBBX32 protein levels, as determined by using a specific antibody anti‐CtrBBX32, in the roots of trifoliate orange plants grown under normal temperature (25 °C) or under cold treatment (4 °C). The Tubulin was determined in parallel using an anti‐Tubulin antibody and server as an input control. b) Growth of yeast cells cotransformed with pGBKT7 (BD)‐CtrBBX32 and pGADT7 (AD)‐CtrCIPK6 on the selection medium synthetic dropout (SD)/‐T(Trp)/‐L(Leu) or SD/‐T(Trp)/‐L(Leu)/‐H(His)/‐A(Ade) added with X‐α‐gal. The positive control was BD‐P53 and AD‐T, while BD‐CtrBBX32 and AD was used as a negative control. c) BiFC assay showing the interaction between CtrBBX32 and CtrCIPK6 in the nucleus. CtrBBX32 was fused with the N‐terminus of yellow fluorescent protein (nYFP), while CtrCIPK6 or CtrCIPK9 was fused with the C‐terminal region of YFP (cYFP). The vectors were coexpressed in *N. benthamiana* leaves, using nYFP + cYFP‐CtrCIPK6, CtrBBX32‐nYFP + cYFP, and CtrBBX32‐nYFP + cYFP‐CtrCIPK9 as negative controls. Scale bars: 25 µm. d) Luciferase (LUC) complementation imaging assay showing the interaction between CtrBBX32 and CtrCIPK6 based on the LUC fluorescence of *N. benthamiana* leaf sections coinfiltrated with the constructs, using nLUC + cLUC‐CtrCIPK6, CtrBBX32‐nLUC + cLUC, and CtrBBX32‐nLUC + cLUC‐CtrCIPK9 as negative controls. e) Pull‐down assay confirming the interaction between CtrBBX32 and CtrCIPK6 in vitro. The recombinant GST‐CtrBBX32 and GST were immobilized on glutathione (GST) agarose beads and then incubated with His‐CtrCIPK6. The protein was detected by immunoblotting with anti‐His and anti‐GST antibodies. Similar results were obtained from three independent experiments. f) Co‐immunoprecipitation (IP) assay for detecting interaction between CtrBBX32 and CtrCIPK6 in vivo. CtrCIPK6‐MYC was coexpressed with CtrBBX32‐GFP or GFP in *N. benthamiana* leaves, from which soluble proteins were extracted and incubated with MYC magnetic beads. CtrCIPK6‐MYC and CtrBBX32‐GFP protein were immunoprecipitated and detected with anti‐MYC and anti‐GFP antibody, respectively. g) In vitro phosphorylation assay showing that CtrCIPK6 phosphorylates CtrBBX32 at Ser 108 site. GST‐CtrCIPK6, GST‐CtrBBX32 or GST‐CtrBBX32^S108A^ were detected by anti‐pSer/pThr antibody and anti‐GST antibody. h) Cell‐free assay revealing the protein levels of GST‐CtrBBX32. GST‐CtrBBX32 was incubated with, in the absence or presence of MG132, total proteins extracted from trifoliate orange hairy roots transformed with 35S:CtrCIPK6 (OE‐*CtrCIPK6*) or empty vector (OE‐EV). GST‐CtrBBX32 level was analyzed by immunoblotting with anti‐GST antibody at the designated time points. Tubulin as an input control, which was detected by using the anti‐Tubulin antibody. i) The autophosphorylation levels of CtrCPK6 are activated by cold. Total proteins were extracted from citrus callus of OE‐*CtrCIPK6*, CtrCIPK6 proteins were separated on 10% SDS‐PAGE added phos‐tag (with or without λ protein phosphatase) and 10% SDS‐PAGE and detected with anti‐Flag antibody. Tubulin served as a control. j) Protein levels of CtrBBX32 in the VIGS lines and the TRV‐EV control grown at 25 °C or at 4 °C for the designated time. Protein level of CtrBBX32 was detected by employing the anti‐CtrBBX32 antibody, using Tubulin as an internal control that was detected using the anti‐Tubulin antibody.

CtrBBX32 protein levels in trifoliate orange roots were detected using a specific antibody (Figure S13, Supporting Information). Notably, the CtrBBX32 protein remained stable at an ambient environment, but exhibited progressive decrease after exposure to cold treatment at 4 °C (**Figure** **6**a), suggesting that cold stress may destabilize CtrBBX32 in trifoliate orange. It has been previously reported that stability of BBX protein was modified at post‐translational level.^[^ ^40^, ^41^ ^]^ It is thus speculated that CtrBBX32 may be degraded by some proteins involved in post‐translational modification pathway. To explore the factors that might regulate CtrBBX32 stability in the presence of cold stress, we used pGBKT7‐CtrBBX32 as a prey to screen a cDNA library derived from trifoliate orange seedlings by yeast two‐hybrid (Y2H) system to identify potential interacting proteins of CtrBBX32. A total of 73 positive clones were obtained and sequenced (Table S2, Supporting Information). Among the candidates, a protein annotated as CBL‐interacting protein kinase 6 (CtrCIPK6) drew our attention, as CIPK is a crucial factor in the calcium signaling pathway that has been shown to play a key role in regulation of cold stress response.^[^ ^42^ ^]^ Moreover, expression pattern analysis demonstrated that *CtrCIPK6* was upregulated under low‐temperature conditions and showed relatively higher expression in roots, and the protein of CtrCIPK6 was localized in the nucleus and cytoplasm based on the YFP signal (Figure S14a–c, Supporting Information), indicating its potential involvement in the interaction with CtrBBX32 during cold stress. We then validated the interaction between CtrCIPK6 and CtrBBX32 via different approaches. Point‐to‐point Y2H assay using the yeast cells coexpressing both BD‐CtrBBX32 and AD‐CtrCIPK6 could grow on the selective medium, indicating that CtrCIPK6 interacted with CtrBBX32 (Figure 6b). BiFC results demonstrated that CtrCIPK6 interacted with CtrBBX32 in the nucleus, whereas no interaction was observed between CtrBBX32 and the other CIPK member of CtrCIPK9 (Figure 6c). LUC complementation image (LCI) showed that coexpression of nLUC‐CtrBBX32 and cLUC‐CtrCIPK6, in which CtrBBX32 and CtrCIPK6 were fused to the N‐terminal half and the C‐terminal half of the LUC reporter, respectively, reconstituted a functional LUC in the *N. benthamiana* leaves, confirming the presence of in vivo interaction between CtrCIPK6 and CtrBBX32 (Figure 6d). In the pull‐down assay, His‐CtrCIPK6 protein could be pulled down and enriched by GST‐CtrBBX32, but not by GST alone, which further confirmed the interaction between CtrCIPK6 and CtrBBX32 in vitro (Figure 6e). In addition, co‐immunoprecipitation (Co‐IP) assay indicated the codetection of CtrCIPK6‐MYC with CtrBBX32‐GFP in *N. benthamiana* leaves, but not with GFP alone, implying the interaction between CtrCIPK6 and CtrBBX32 in planta (Figure 6f). Collectively, these results corroborated the notion that CtrCIPK6 physically interacted with CtrBBX32 in plants. On the other hand, both Y2H and BiFC assays revealed that no point‐to‐point interaction was observed between CtrCIPK6 and CtrZAT10 (Figure S15, Supporting Information).

Given that CtrBBX32 interacted with the protein kinase of CtrCIPK6, we assumed that CtrBBX32 might act as a potential substrate of CtrCIPK6 and was possibly phosphorylated by CtrCIPK6. To confirm this assumption, we conducted an in vitro phosphorylation assay using phosphoserine/threonine antibody, which showed that the recombinant GST‐CtrCIPK6 protein phosphorylated GST‐CtrBBX32 (Figure 6g). To determine the potential phosphorylation site on CtrBBX32 targeted by CtrCIPK6, we incubated recombinant GST‐CtrBBX32 and GST‐CtrCIPK6, followed by a liquid chromatography‐tandem mass spectrometry analysis. As a consequence, the Serine (Ser)‐108 of the CtrBBX32 protein was identified as a putative phosphorylation site targeted by CtrCIPK6 (Figure S16, Supporting Information). When Ser 108 was mutated to alanine (BBX32^S108A^), a non‐phosphorylatable form, the phosphorylation intensity of CtrBBX32 by CtrCIPK6 was dramatically decreased compared with the wild‐type CtrBBX32 (Figure 6g). These results indicate that CtrBBX32 is phosphorylated by CtrCIPK6 in vitro.

Phosphorylation typically alters the activity or stability of the target protein. The cell free degradation assay was employed to determine whether CtrCIPK6 affects stability of the CtrBBX32. The total protein from OE‐EV and OE‐*CtrCIPK6* roots of trifoliate orange was extracted and incubated respectively with GST‐CtrBBX32 protein. The reaction mixtures were collected at designated periods and detected using a GST antibody. The degradation rate of GST‐CtrBBX32 incubated with the total protein of OE‐*CtrCIPK6* was faster than that of GST‐CtrBBX32 incubated with OE‐EV total protein. Meanwhile, the degradation process of GST‐CtrBBX32 was inhibited by adding MG132, indicating that CtrCIPK6 promotes the degradation of CtrBBX32 via the 26S proteasome pathway (Figure 6h). Elevated autophosphorylation levels of CIPK indicated conformational activation of the kinase domain under specific conditions, triggering release of autoinhibition (e.g., through NAF domain disengagement) and enhanced phosphorylation capacity toward downstream substrates. To investigate the cold‐induced changes in autophosphorylation level of CtrCIPK6, we subjected Flag‐tagged OE‐*CtrCIPK6* citrus callus to either ambient temperature (control) or low‐temperature treatment. The results demonstrated a significant enhancement of autophosphorylation level of CtrCIPK6 under cold stress, with peak activity observed at 1 h of cold exposure. Notably, the slow‐migrating bands detected in cold‐treated OE‐*CtrCIPK6* callus samples were nearly abolished upon incubation with λ protein phosphatase, indicating that the reduced mobility was due to phosphorylation (Figure 6i). These findings collectively demonstrate that cold stress enhances CtrCIPK6 activity. We propose that this cold‐activated autophosphorylation of CtrCIPK6 may subsequently regulate the protein stability of CtrBBX32. We further found that the abundance of CtrBBX32 protein in *CtrCIPK6*‐VIGS lines was significantly higher than that in TRV‐EV control under cold stress (4 °C) treatment, while there was not significantly different between the VIGS lines and the TRV‐EV control at 25 °C (Figure 6j). These results indicate that CtrCIPK6 promotes the degradation of CtrBBX32 protein in response to cold stress.

### 2.8 CtrCBL1/CtrCIPK6 Phosphorylates Ser‐108 of CtrBBX32 and Relieves its Inhibition of *CtrSTP1*

**Figure 7:**
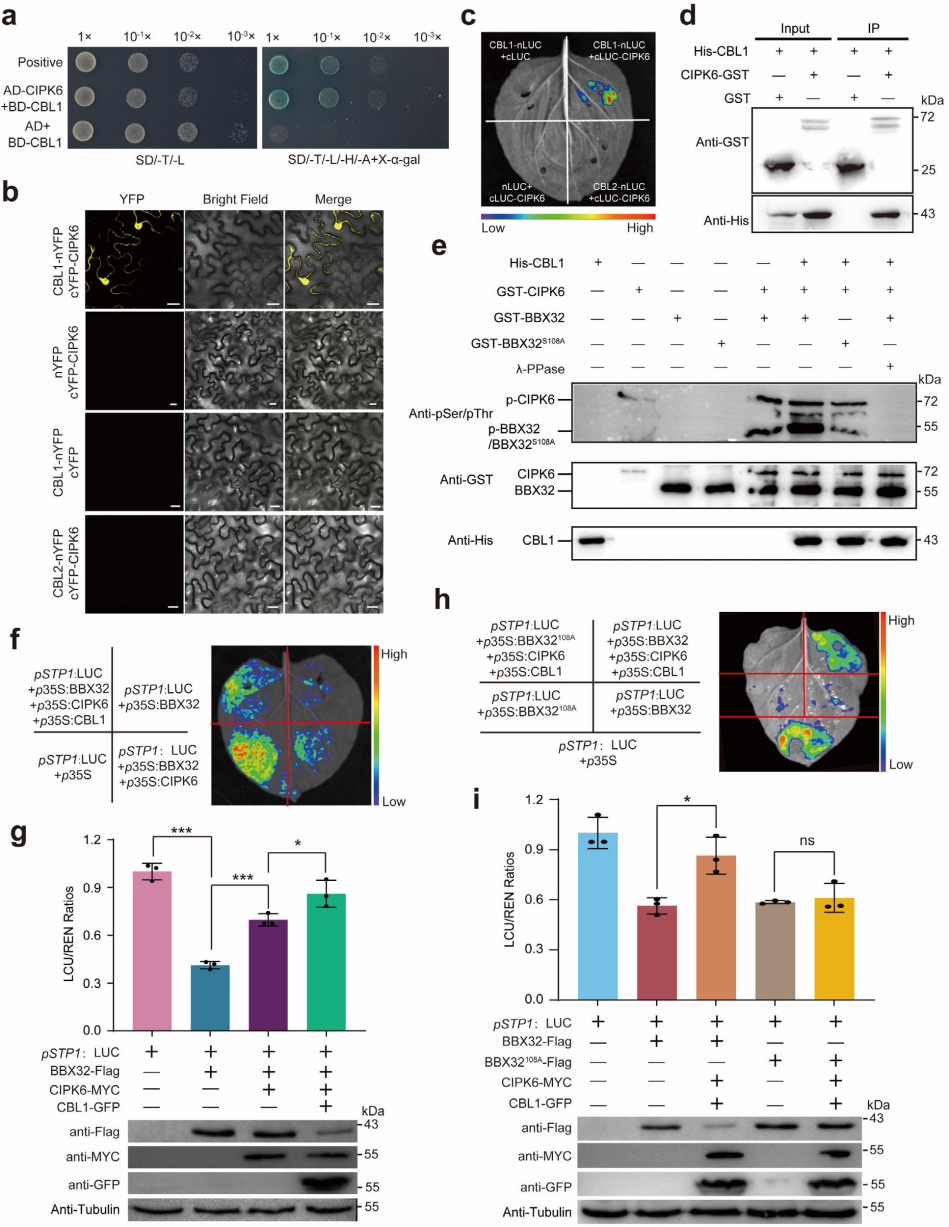
Phosphorylation of CtrBBX32 at Ser‐108 by CtrCBL1/CtrCIPK6 complex promotes protein degradation and relieves its transcriptional inhibition. a) Y2H assay showed that CtrCIPK6 interacted with CtrCBL1. The fusion vector pGBKT7‐CtrCIPK6 and pGADT7‐CtrCBL1 were cotransformed into yeast strain and then grown on SD/‐Trp/‐Leu and SD/‐Trp/‐Leu/‐His/‐Ade + X‐α‐gal medium. The positive control was pGBKT7‐P53 and pGADT7‐T, and pGBKT7 (BD)‐CtrCIPK6 and pGADT7 were used as negative controls. b) BiFC assay showed that CtrCIPK6 interacted with CtrCBL1 in the nucleus and cytoplasm, but did not interact with CtrCBL2. CtrCIPK6 and CtrCBL1/CtrCBL2 were fused to the C‐ or N‐terminus of YFP and coexpressed in *N. benthamiana* leaves, and the fluorescence of YFP was detected by confocal microscopy. Scale bars: 25 µm. c) LUC (luciferase) complementation imaging assay was used to detect the interaction between CtrCIPK6 and CtrCBL1 in *N. benthamiana* leaves with designated coexpression infiltration, using nLUC + cLUC‐CtrCIPK6, CtrCBL1‐nLUC + cLUC, and CtrCBL2‐nLUC + cLUC‐CtrCIPK6 as negative controls. d) Pull‐down assay confirmed the interaction between CtrCIPK6 and CtrCBL1 in vitro. The recombinant GST‐CtrCIPK6 or GST was immobilized on glutathione agarose and then incubated with His‐CtrCBL1. e) In vitro phosphorylation analysis of GST‐CtrBBX32 or GST‐CtrBBX32^S108A^ (a mutation from serine to alanine at 108 site) by the CtrCBL1/CtrCIPK6 complex. GST‐CtrCIPK6 was incubated with GST‐CtrBBX32 or GST‐CtrBBX32^S108A^ in the kinase buffer without or with λ‐PPase, in the presence or absence of His‐CtrCBL1, at 30 °C for 60 min. Phosphorylated GST‐CtrBBX32 and GST‐CtrBBX32^S108A^ were detected by anti‐pSer/pThr antibody. GST‐CtrCIPK6, GST‐CtrBBX32, and GST‐CtrBBX32^S108A^ were detected by anti‐GST antibody, and His‐CtrCBL1 was detected by anti‐His antibody. f–i) Luciferase (LUC) bioluminescence imaging (f) and (h) or LUC/REN ratios (g) and (i) of the *N. benthamiana* leaves infiltrated with 35S:CtrBBX32‐Flag or 35S:CtrBBX32^S108A^‐Flag and LUC reporter driven by the promoter of *CtrSTP1* (p*CtrSTP1*:LUC), along with CtrCIPK6‐MYC alone or CtrCIPK6‐MYC and CtrCBL1‐GFP together. Infiltration of p*CtrSTP1*:LUC and empty vector (EV) was used as a control. Protein levels of CtrBBX32/CtrBBX32^S108A^, CtrCIPK6, and CtrCBL1 were detected using the antibodies against Flag (anti‐Flag), MYC (anti‐MYC), and GFP (anti‐GFP), respectively. +, presence; −, absence. Error bars denote ± standard deviation (SD, *n* = 3). Two‐tailed Student^’^s *t*‐test was conducted for analyzing the significant difference (*P < 0.05, ***P < 0.001; *P* > 0.05, ns, no significance).

The CBL‐CIPK module has been well demonstrated to act as a highly conserved machinery involved in the regulation of various biological and physiological processes in higher plants.^[^ ^43^, ^44^, ^45^, ^46^ ^]^ Therefore, the Y2H assay was conducted to determine the CtrCBL members that could interact with CtrCIPK6. The results showed that CtrCBL1, CtrCBL4, CtrCBL5, and CtrCBL7 physically interacted with CtrCIPK6 (Figure S17a, Supporting Information). Further integrated analysis of cold stress transcriptome data and tissue‐specific expression profiles demonstrated that* CtrCBL1* was uniquely upregulated under low‐temperature conditions and exhibited relatively higher expression levels in root tissues (Figure S17b,c, Supporting Information). This expression pattern paralleled the cold‐induced upregulation and root‐enriched expression of* CtrCIPK6*. Based on these findings, *CtrCBL1* was selected as the primary candidate for subsequent functional studies. CtrCBL1 was further demonstrated to be localized in the nucleus and cytoplasm based on the YFP signal visualization in the subcellular localization experiment (Figure S12d, Supporting Information). Yeast two‐hybrid analysis confirmed a physical interaction between CtrCBL1 (BD fusion) and CtrCIPK6 (AD fusion), based on colony growth under selective conditions (**Figure** **7**a). BiFC assay showed that CtrCBL1 interacted with CtrCIPK6 in the nucleus and cytoplasm, while no interaction signal was detected between CtrCIPK6 and the other negative control member of CtrCBL2 (Figure 7b). The interaction between CtrCBL1 and CtrCIPK6 was further validated through the LCI and pull‐down assays (Figure 7c,d).

To gain deeper insights into the impact of the protein complex formed by CtrCBL1 and CtrCIPK6 on the phosphorylation of CtrBBX32, we performed the phosphorylation experiment by incubating CtrBBX32 or CtrBBX32^S108A^ as substrates with CtrCIPK6 in the presence or absence of CtrCBL1. CtrCIPK6 could phosphorylate CtrBBX32, and the intensity was substantially enhanced in the presence of CtrCBL1, implying that CtrCBL1 promoted the CtrCIPK6‐mediated phosphorylation of CtrBBX32. However, the phosphorylation of CtrCIPK6 on CtrBBX32^S108A^ was slightly and severely impaired relative to CtrBBX32 in the absence or presence of CtrCBL1, respectively (Figure 7e). Next, efforts were made to assess the effect of CtrCIPK6‐mediated phosphorylation, in the absence or presence of CBL1, on the transcriptional regulation of *CtrSTP1* promoter (p*CtrSTP1*) by CtrBBX32 by using the dual luciferase transient assay. Similar to the results mentioned above (Figure 3e), coexpression of p*CtrSTP1* and CtrBBX32 resulted in the reduction of promoter activity relative to the control. However, when CtrCIPK6 was included in the infiltration (CtrBBX32 + CtrCIPK6 + p*CtrSTP1*), the repression of promoter activity was evidently mitigated, as reflected by the augmented fluorescence intensity, in comparison with the infiltration of p*CtrSTP1* and CtrBBX32. Interestingly, addition of CtrCBL1 led to a substantial reduction of CtrBBX32 protein level and a concurrent drastic increase in the promoter activity, suggesting that the transcriptional inhibition of CtrBBX32 on *CtrSTP1* promoter was further released by CtrCBL1 (Figure 7f,g). Like CtrBBX32, coexpression of CtrBBX32^S108A^ and p*CtrSTP1* also led to a sharp inhibition of promoter activity relative to the control. However, in comparison with the reaction observed in the control, the mitigation effect of CtrCIPK6 and CtrCBL1 on CtrBBX32‐mediated p*CtrSTP1* repression was completely abolished when these proteins were coinfiltrated with CtrBBX32^S108A^ (Figure 7h,i). Taken together, these results indicate that CtrCBL1/CtrCIPK6‐mediated phosphorylation of CtrBBX32 at the Ser108 residue promoted the CtrBBX32 protein degradation and consequently relieved its transcriptional repression of *CtrSTP1*.

### 2.9 CtrCIPK6 Positively Modulates Cold Tolerance

**Figure 8:**
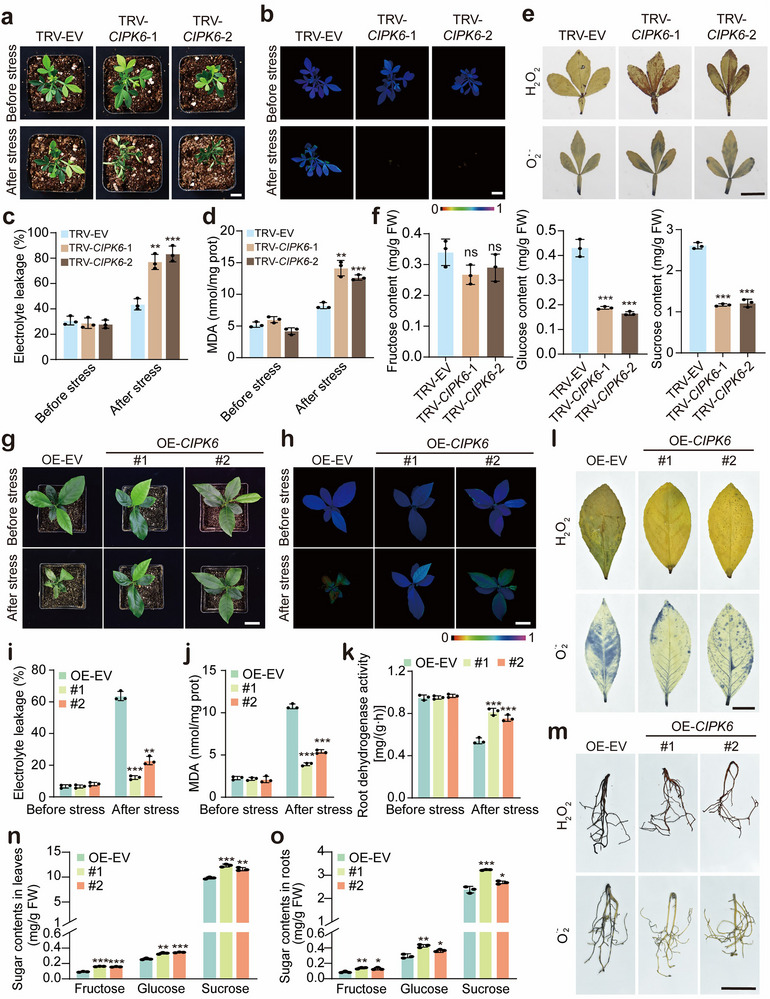
CtrCIPK6 enhances plant cold tolerance. a) Phenotypes of plants from the virus‐induced gene silencing (VIGS) lines (TRV‐*CtrCIPK6*‐1, TRV‐*CtrCIPK6*‐2) and the TRV‐EV control before cold treatment (before stress) and after exposure to −6 °C for 6 h, followed by growth recovery at room temperature for 12 h (after stress). Three independent plants were used for each line. Scale bar: 2 cm. b–d) Chlorophyll fluorescence imaging (b), EL (c), and MDA content (d) of the tested lines before and after the cold treatment. Scale bar: 2 cm. e) In situ detection of H_2_O_2_ (upper panels) and O_2_^•−^ (lower panels) in the leaves of the tested plants after the cold treatment. Scale bar: 2 cm. f) The content of fructose, glucose, and sucrose in the roots of the tested lines. g) Plant phenotypes of the OE lines (OE‐*CtrCIPK6*) and the OE‐EV (control plant) before cold treatment (before stress) and after exposure to −2 °C for 6 h, followed by growth recovery at room temperature for 12 h (after stress). Scale bars: 2 cm. h–k) Chlorophyll fluorescence imaging (Scale bar = 2 cm) (h), EL (i), MDA content (j), and root dehydrogenase activity (k) of the tested lines before and after the cold treatment. l, m) In situ detection of H_2_O_2_ and O_2_^•−^ by histochemical staining with DAB (upper panels) and NBT (lower panels) in the leaves (l) and roots (m) of the tested plants after the cold treatment (Scale bar: 2 cm). n, o) The contents of fructose, glucose, and sucrose in the in the leaves (n) and in the roots (o) of the test lines before cold treatment. Error bars denote ± standard deviation (SD, *n* = 3). Two‐tailed Student^’^s *t*‐test was conducted for analyzing the significant difference (*P < 0.05, **P < 0.01, ***P < 0.001).

To further clarify the function of CtrCIPK6 in cold tolerance, we used the VIGS approach to knock down *CtrCIPK6* in trifoliate orange. No morphological difference was observed between the TRV‐EV control and the *CtrCIPK6‐*VIGS lines (TRV‐*CtrCIPK6*). However, when exposed to cold stress (−6 °C for 6 h) followed by recovery at ambient environment for 16 h, the *CtrCIPK6‐*VIGS lines were more seriously damaged compared with the TRV‐EV control, as manifested by severe leaf curling and necrosis (**Figure** **8**a). In addition, weaker chlorophyll fluorescence, higher EL and MDA, along with greater accumulation of ROS, were detected in the VIGS plants in the presence of cold treatment (Figure 8b–e). In addition, the transcript levels of *CtrZAT10* and *CtrSTP1* were significantly downregulated in the *CtrCIPK6*‐silenced plants after 4 °C treatment for 6 h, in line with the repression of *CtrCIPK6* expression (Figure S18a, Supporting Information). Consistently, the glucose and sucrose contents in the VIGS‐*CtrCIPK6* lines were significantly lower than those in the TRV‐EV control, whereas the fructose content exhibited a slight decrease with no statistical difference (Figure 8f). In summary, silencing of *CtrCIPK6* led to enhanced cold sensitivity by reducing the soluble sugar contents through repressing the expression of *CtrZAT10* and *CtrSTP1*, implying that *CtrCIPK6* might function positively in modulation of cold tolerance.

We employed genetic transformation to introduce the *CtrCIPK6* gene into cold‐sensitive lemon plants, successfully obtaining transgenic lemon lines (#1 and #2) with stable overexpression of CtrCIPK6 (Figure S18b, Supporting Information). Under normal growth conditions, no significant differences in growth performance were observed between *CtrCIPK6*‐overexpressing transgenic lemon plants and the control (OE‐EV). However, when exposed to low‐temperature stress (−2 °C for 6 h), the control plants exhibited leaf wilting and water‐soaking symptoms, whereas the *CtrCIPK6*‐overexpressing transgenic lemon plants showed no visible damage (Figure 8g). Correspondingly, following the cold treatment, the control plants showed weaker chlorophyll fluorescence, higher EL and MDA content, reduced root dehydrogenase activity, and significantly increased ROS accumulation in both leaves and roots relative to the transgenic plants (Figure 8h–m). In addition, significantly higher concentrations of fructose, glucose, and sucrose were detected in both leaves and roots of OE‐*CtrCIPK6* lines than in the OE‐EV control (Figure 8n,o). Taken together, these findings suggest that *CtrCIPK6* positively regulates cold tolerance of trifoliate orange.

## 3 Discussion

Cold stress persists as a primary environmental constraint, significantly limiting global crop productivity and plant distribution.^[^ ^8^, ^47^ ^]^ It is widely accepted that osmotic adjustment emerges as an evolutionarily conserved physiological mechanism for maintaining cellular homeostasis through accumulation of various compatible solutes like betaine, proline, and soluble sugars.^[^ ^10^ ^]^ Studies have shown that soluble sugars contribute significantly to osmotic regulation and ROS scavenging during plant stress adaptation.^[^ ^48^, ^49^ ^]^ In this study, we identified *CtrSTP1* as a cold‐induced sugar transporter gene that was primarily expressed in roots of *Citrus trifoliata*, and illustrated its role and regulation mechanisms in soluble sugar transport for plant cold tolerance.

Under cold stress, plants preferentially convert fixed CO_2_ into soluble sugars rather than storing it as starch,^[^ ^50^ ^]^ coinciding with starch degradation across multiple tissues.^[^ ^51^, ^52^ ^]^ This coordinated metabolic reprogramming results in significant accumulation of soluble sugars,^[^ ^8^ ^]^ which alleviate energy deficits and maintain cellular osmotic potential under cold stress. The utilization of these sugars is critically dependent on sugar transporters, which govern long‐distance transport and intertissue distribution of sugars during plant stress responses.^[^ ^53^ ^]^ This study identified *CtrSTP1*, a cold‐induced sugar transporter gene that is predominantly expressed in the root cortical cells of *C. trifoliata*, a cold‐resistant plant species widely utilized as rootstock for citrus production. Functional characterization confirmed its hexose transport activity, and genetic transformation experiments further demonstrated that *CtrSTP1* enhances cold tolerance by promoting soluble sugar accumulation in root tissues. Roots, which serve as critical sink organs and signaling centers, exhibit increased susceptibility to cold stress in cortical tissues. The cortex not only functions as the primary pathway for resource transport within roots but also displays heightened vulnerability to cold‐induced membrane damage due to its thin‐walled parenchymatous structure, leading to characteristic chilling injury symptoms such as impaired water and mineral uptake as well as reduced tree vigor. While cold‐stressed roots are known to accumulate soluble sugars as adaptive stress responses,^[^ ^54^, ^55^, ^56^ ^]^ and the regulatory roles of sugar transporters in this process have recently garnered increasing attention,^[^ ^9^, ^57^, ^58^ ^]^ the functional mechanisms of root‐localized sugar transporters under cold stress remain largely unexplored. Moreover, the tissue‐specific expression patterns of *STP1* genes have been documented across various species. For instance, *AtSTP1* in *Arabidopsis* exhibits guard cell‐specific expression, providing sugars for starch accumulation and regulating light‐induced stomatal opening.^[^ ^17^ ^]^ In rice (*Oryza sativa*), *OsSTP1* demonstrates predominant expression in leaf sheaths, stems, and nodes during the grain‐filling stage, contributing to increased seed‐setting rates and yields.^[^ ^16^ ^]^ *CsHT1* shows exclusive expression in pollen, with translation occurring specifically during pollen germination and tube elongation in cucumber (*Cucumis sativus*).^[^ ^59^ ^]^ This species‐specific divergence in *STP1* expression patterns indicates that the cold tolerance of *C. trifoliata* may arise from the evolutionary tissue‐specific adaptation of *CtrSTP1*, thereby contributing to its exceptional cold hardiness. Our findings elucidate a novel functional connection between the hexose transport activity of CtrSTP1 and cold tolerance in *C. trifoliata*.

Sucrose serves as the principal form for the transport of photosynthates from source to sink in the majority of plant species. The hexose transport of STP‐mediated phloem sugar unloading process involves apoplastic compartment cell wall invertases (CWINVs), which facilitate the transport of hexoses (e.g., glucose, fructose) derived from hydrolyzed sucrose.^[^ ^60^, ^61^ ^]^ Research has shown that heat stress‐induced suppression of *CWINV* expression reduces the sink strength of tomato fruit and disrupted the source–sink balance.^[^ ^62^ ^]^ Additionally, our earlier work has demonstrated that cold stress specifically upregulated the expression of *CtrCWINV1* and *CtrCWINV5* in *C. trifoliata* roots,^[^ ^63^ ^]^ indicating that cold‐induced *CWINV* upregulation enhanced apoplastic sucrose hydrolysis into hexoses to optimize sugar allocation under low‐temperature conditions. Moreover, the hydrolysis of sucrose into hexoses is a preferred strategy for plant sugar utilization during cold stress responses.^[^ ^64^, ^65^ ^]^ Low‐molecular‐weight hexoses, characterized by their high osmotic activity, rapidly accumulate in the cytoplasm to mitigate cold‐induced osmotic imbalance and membrane damage,^[^ ^60^, ^66^ ^]^ while simultaneously contributing to ROS scavenging.^[^ ^67^ ^]^ Furthermore, their direct entry into the glycolytic pathway circumvents the ATP expenditure associated with sucrose catabolism, providing a critical metabolic advantage under energy‐constrained cold conditions. This is supported by the lack of cold‐induced expression in sucrose transporter genes observed in *C. trifoliata*. We hypothesize that the cold‐induced *CWINVs* and *CtrSTP1* coordinated module in the roots of *C. trifoliata* functionally facilitates cold adaptation by enhancing apoplastic sucrose hydrolysis and subsequent hexose transport. Future investigations should aim to clarify the mechanisms by which cold signaling components regulate the coordination between CWINVs and STPs, as well as examine the evolutionary conservation of this module across plant species.

Transcriptional regulation plays a critical role in plant cold stress responses.^[^ ^68^ ^]^ Since the cold‐induced upregulation of *CtrSTP1* transcript levels enhances cold tolerance, elucidating its upstream regulatory mechanisms becomes essential. This study identifies CtrZAT10 and CtrBBX32 as key upstream regulators of *CtrSTP1*. CtrZAT10 activates *CtrSTP1* transcription by directly binding to the CTGTAACAGTA motif within its promoter region, thereby improving plant cold tolerance. In contrast, CtrBBX32 suppresses *CtrSTP1* expression through interaction with G‐box elements in its promoter. Moreover, CtrBBX32 mitigates CtrZAT10‐mediated activation by targeting G‐box elements in the *CtrZAT10* promoter, ultimately coordinating reduced sugar transport and diminished plant cold hardiness. Previous studies elucidated the antagonistic regulation of shared target genes by multiple transcription factors, highlighting their pivotal roles in plant development and stress adaptation. For example, *VvSWEET15*, a key hexose transporter during grape berry ripening, is coordinately regulated by the transcriptional repressor VvERF105 and the activator VvNAC72.^[^ ^69^ ^]^ AtMYB43 constitutively suppresses *AtCBF* expression under normal conditions; however, cold‐induced degradation of AtMYB43 alleviates its antagonistic interaction with ICE1, thereby facilitating ICE1‐mediated activation of *AtCBF* in *Arabidopsis*.^[^ ^70^ ^]^ In strawberries, FvNAC073 and FvCMB1L competitively bind to the promoters of *FvSPS1* and *FvSUS2*, antagonistically regulating sucrose accumulation.^[^ ^71^ ^]^ These findings underscore that antagonistic regulation facilitates precise and dynamic orchestration of gene expression in response to developmental signals and environmental stimuli, thus maintaining cellular homeostasis while avoiding extreme states such as hyperactivation or complete silencing. Accordingly, we observed that the protein level of CtrBBX32 declined under cold stress, whereas the expression of *CtrZAT10* and *CtrSTP1* exhibited significant induction. We speculate that under favorable conditions, plants tend to retain more carbon resources in their aboveground tissues, while distributing basic sugar to the root system to sustain critical physiological activities. When subjected to cold conditions, the roots will trigger the high‐level expression of *CtrSTP1*, which mediates the accumulation of soluble sugars to protect root tissues from cold‐induced damage. Therefore, the findings elucidated the novel molecular mechanism through which two transcription factors, CtrBBX32 and CtrZAT10, accurately regulate the expression pattern of *CtrSTP1* in response to cold stress, thereby precisely coordinating the root tissue‐specific distribution of soluble sugars for plant cold tolerance.

Post‐translational modifications extensively regulate protein functions. We demonstrated that cold stress significantly decreases the protein abundance of CtrBBX32, thereby releasing its suppression on the transcriptional activity of *CtrSTP1*. Through Y2H screening, CtrCIPK6 was identified as a potential interaction partner of CtrBBX32. Subsequent multiple interaction assays validated their physical interaction both in vitro and in vivo. Further analysis revealed that CtrCIPK6 specifically phosphorylates CtrBBX32 at Serine 108 (Ser108), thereby mediating its stability and transcriptional regulatory function. These findings collectively support a molecular mechanism wherein CtrCIPK6 dynamically modulates CtrBBX32 protein levels in response to cold stress via a phosphorylation‐degradation cascade signaling pathway.

CIPK kinases decode Ca^2^⁺ signals via interaction with CBL proteins and execute their functions by phosphorylating downstream targets.^[^ ^72^ ^]^ By integrating Y2H assays with transcriptomic evidence of cold‐inducible *CtrCBLs* and their root‐predominant expression patterns, we identified the cytoplasm and nucleus localized CtrCBL1, which is consistent with the reported subcellular localization of its ortholog CsCBL1 in sweet orange,^[^ ^73^ ^]^ as the key calcium sensor mediating cold signaling‐induced activation of CtrCIPK6 (Figure S17a–c, Supporting Information). Studies have shown that cold stress triggers extracellular Ca^2^⁺ influx in plant roots, thereby activating downstream defense mechanisms.^[^ ^74^ ^]^ However, the potential regulatory roles of this process in sugar transport and accumulation remain to be elucidated. Our further investigation revealed that the CtrCBL1/CtrCIPK6 complex alleviates the suppression of *CtrSTP1* by CtrBBX32 through phosphorylation‐dependent modification. Genetic transformation experiments further confirm that calcium signaling, mediated by the sensor complex CtrCBL1/CtrCIPK6, enhances the cold tolerance of *C. trifoliata* by integrating into the CtrBBX32‐mediated CtrZAT10‐*CtrSTP1* regulatory module, thereby promoting sugar accumulation in root tissues (Figure 8; Figure S18, Supporting Information). The results establish a functional linkage between cold‐induced calcium signaling and sugar transport regulation, providing mechanistic insights into calcium signaling‐mediated sugar accumulation as a strategy to improve plant low‐temperature adaptation.

**Figure 9:**
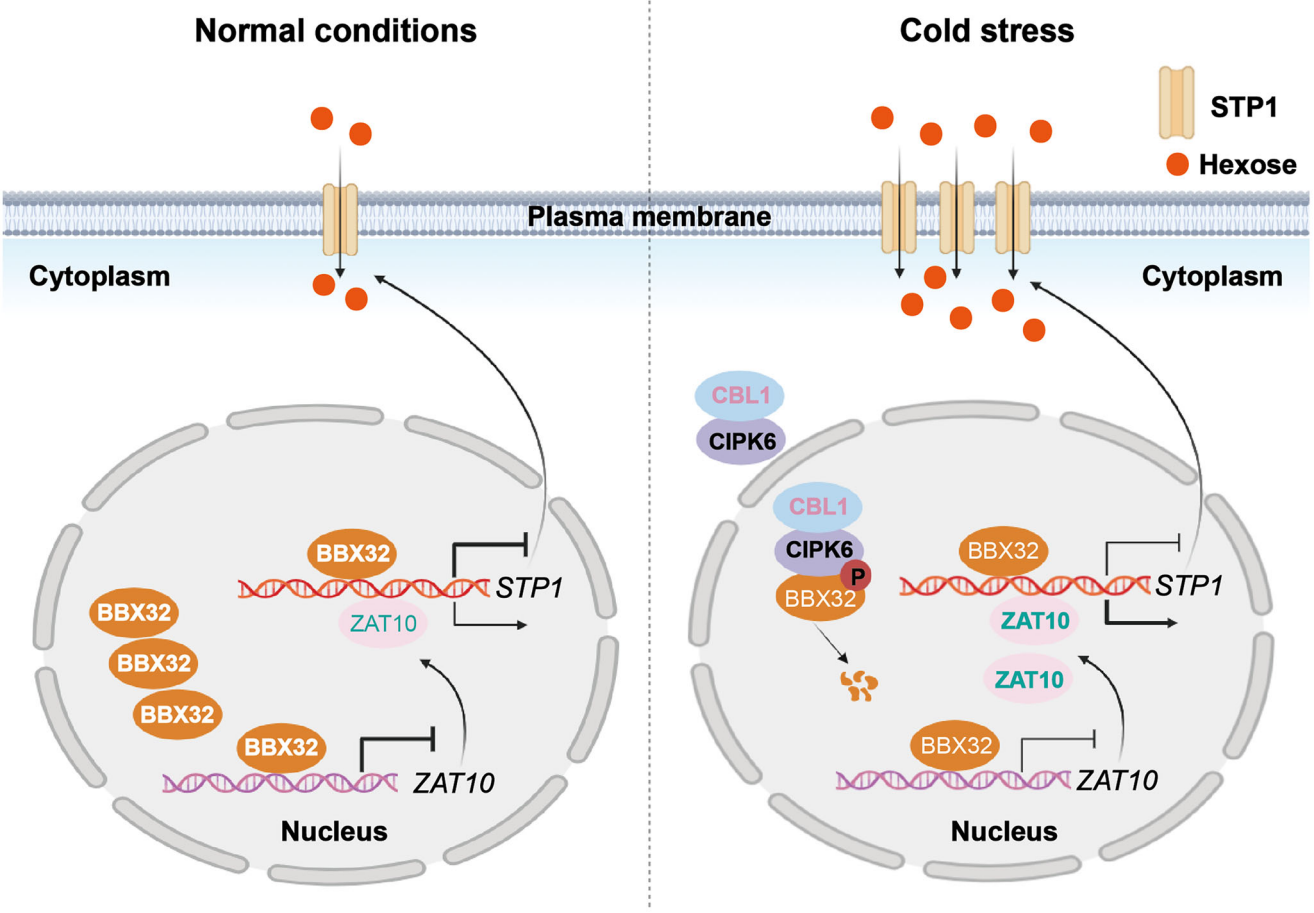
A working model illustrating the regulation of *CtrSTP1* and sugar accumulation under cold stress. Under normal growth conditions, CtrBBX32 repressed the expression of *CtrZAT10* and *CtrSTP1* to maintain moderate soluble sugar content. In response to the cold stress, CtrCBL1/CtrCIPK6 complex was activated. CtrCIPK6 then interacts with and phosphorylates CtrBBX32, facilitating its degradation and releasing its transcriptional repression on *CtrZAT10* and *CtrSTP1*. CtrZAT10, in turn, activates the expression of *CtrSTP1*, which promotes hexose transport into cells, thereby enhancing cold tolerance.

Based on these findings, we propose a working model in which the calcium sensor CtrCBL1/CtrCIPK6‐mediated regulation of CtrBBX32 governs cold‐adaptive sugar accumulation in root tissues through the CtrZAT10‐*CtrSTP1* module (**Figure** **9**). Under ambient conditions, CtrBBX32 suppresses the expression of both *CtrZAT10* and *CtrSTP1* to maintain basal sugar levels in roots. Upon exposure to cold stress, CtrCBL1/CtrCIPK6 complex reduces the stability of CtrBBX32 via Ser108 phosphorylation, thereby alleviating its repressive effect on downstream targets. This allows for CtrZAT10‐driven transcriptional activation of *CtrSTP1*, enhancing hexose transport and conferring cold tolerance. Our study provides novel mechanistic insights into calcium signaling‐mediated sugar homeostasis in root tissues during cold adaptation and identifies potential genetic targets for improving crop cold resistance.

## 4 Experimental Section

### Plant Materials and Growth Conditions

Seeds of trifoliate orange (*Citrus trifoliata*) and lemon (*Citrus× limon*) were collected from the Citrus Breeding Center at Huazhong Agricultural University (Wuhan, China). Sweet orange callus and *N. benthamiana* were stored in the laboratory. For the cold treatment, the trifoliate orange and lemon plants grown at normal ambient temperature were transferred to a growth incubator (HP400G; Ruihua, Wuhan, China) set at −6 °C to 4 °C. Samples were harvested at the specified time points, quickly frozen in liquid nitrogen, and stored at −80 °C for further analysis.

### RNA Extraction and Real‐Time Quantitative PCR (RT‐qPCR) Analysis

For the analysis of gene expression, the following reagents, instruments and procedures were applied. Total RNA was extracted by Trizol‐reagent (Invitrogen, Carlsbad, USA), and then synthesized into first chain cDNA by using the HiScript II Q RT SuperMix (Cat. R223; Vazyme, Nanjing, China). Real‐time quantitative PCR was performed on an ABI7500 system (Applied Biosystems, Foster City, CA, USA) by using the ChamQ SYBR qPCR Master Mix (Cat. Q771; Vazyme, Nanjing, China). The amplification reaction procedure was set at 95 °C for 5 min, followed by 40 cycles at 95 °C for 15 s and 60 °C for 30 s. *Actin* was used as the internal reference gene in all experiments, and the expression levels of the detected genes were normalized according to the 2^−ΔΔCT^ algorithm.^[^ ^75^ ^]^ Primers used in this study are listed in Table S3 (Supporting Information), unless otherwise stated.

### Subcellular Localization Assays

The coding sequences (CDSs) of *CtrSTP1*, *CtrBBX32*, *CtrZAT10*, *CtrCIPK6*, and *CtrCBL1* without stop codon were cloned, and they were inserted into pRI101‐YFP vector driven by CaMV 35S promoter. These vectors were introduced into *A. tumefaciens* strain GV3101, respectively. The fusion vector and the empty vector were injected into the tobacco leaves, along with the nuclear marker (35S:VirD2NLS‐mCherry) or membrane marker (35S:CBL1n‐OFP).^[^ ^76^, ^77^ ^]^ The transiently transformed *N. benthamiana* was cultured in the growth chamber for 2–3 days. In order to detect plasma membrane signals, protoplast isolation was performed according to previous reports.^[^ ^78^ ^]^ Then the fluorescence signal was observed by a laser confocal scanning microscope (Leica TCS SP8, Mannheim, Germany). GFP fluorescence was excited with a 488 nm laser and detected within the 500–550 nm emission range, while YFP signal was excited using a 514 nm laser line with detection set at 525–560 nm. For mCherry, fluorescence detection was conducted at an excitation wavelength of 561 nm and a detection range of 590–650 nm.

### In Situ Hybridization

The roots of 2‐month‐old wild‐type seedlings were fixed for in situ hybridization assay. RNASweAMI plant in situ hybridization (BCIP/nitrogen blue tetrazolium (NBT)) detection kit (Cat. GF011; Servicebio, Wuhan, China) was used to detect the specific localization of *CtrSTP1* in roots.

### Heterologous Expression of CtrSTP1 in Yeast

The CDS of *CtrSTP1* was inserted into the vector pDR196, and then the fusion vector and empty vector were transformed into the hexose/sucrose transport‐deficient yeast strain CSY4000.^[^ ^38^ ^]^ Incubation was performed in SD/‐Ura medium with 2% maltose (w/v) as the only carbon source for 2–3 days. The yeast cells were then diluted four times (10×) in succession on a solid SD/‐Ura medium containing 2% maltose, glucose, fructose, and sucrose as the sole carbon source. The yeast cells were cultured at 30 °C for 3 days and photographed.

^14^C‐Glc uptake assay was carried out according to the previous description with slight changes.^[^ ^78^ ^]^ Yeast strain CSY4000 harboring pDR196‐CtrSTP1 or pDR196 (empty vector) was grown in liquid minimal medium containing maltose at 30 °C to an optical density at 623 nm of ≈0.8. Cells were harvested by centrifugation and washed twice with 25 mm sodium phosphate buffer (pH 5.0), and suspended in the same buffer to an optical density at 623 nm of 20. The uptake experiment was performed by adding ^14^C‐Glc (0.02 µCi) into the yeast cells to a final specified concentration (100 mm) and incubated in a 30 °C shaker. The samples were collected at a specified time interval, centrifuged at 4 °C, rinsed twice with distilled water, and finally suspended in 200 µL of distilled water, and then placed in a scintillation tube containing liquid scintillation cocktails. Liquid scintillation counting was performed on a multifunctional scintillation counter (PerkinElmer, Tri‐Carb 2810TR, Singapore).

### Virus‐Induced Gene Silencing (VIGS)

A TRV‐based system was employed for VIGS in trifoliate orange.^[^ ^6^ ^]^ Partial cDNA fragments of target genes—*CtrSTP1* (580‐959 bp), *CtrBBX32* (401‐625 bp), *CtrZAT10* (81‐240 bp) and *CtrCIPK6* (597‐761 bp)—were amplified and individually cloned into the pTRV2 vector, after which each recombinant vector was transformed into *A. tumefaciens* strain GV3101 (Weidi Biotechnology, Shanghai, China). For plant infection, the bacterial cultures harboring pTRV1 and individual pTRV2‐gene constructs were centrifuged and re‐suspended to OD_600_ = 0.8. The suspensions of pTRV1 and each pTRV2 construct were then mixed 1:1 (v/v) and coincubated in darkness for 3–4 h at room temperature. Subsequently, the germinated trifoliate orange seedlings that had been cultured in dark for one month were slightly wounded using sterile needles, vacuum‐infiltrated (10 min per cycle, 3 cycles) in the infection solution, rinsed with deionized water, followed by incubation in dark under high humidity for 3 days before they were transplanted into pots containing a soil–vermiculite–perlite mixture (3:1:1, v/v/v). After one month of growth, the transgenic plants were screened by genomic PCR, and the positive plants were subjected to RT‐qPCR analysis to quantify transcript levels of target genes. Two lines composed of three independent plants that exhibited >60% silencing efficiency were generated and selected for further treatment and analysis.

### Generation of Transgenic Plants by *A. tumefaciens*‐Mediated Transformation

To obtain overexpressed transgenic lemons, the CDS of *CtrSTP1* and *CtrCIPK6* (excluding the stop codon) were inserted into the pK7WG2D vector and placed under the control of the CaMV 35S promoter. The expression vector was transformed into the *A. tumefaciens* strain GV3101 and then used for lemon transformation as previously reported.^[^ ^79^ ^]^ Transgenic plants were screened on MT medium (Cat. PM1161; Coolaber, Beijing, China) containing 50 µg mL^−1^ kanamycin. Positive transformants were identified by immunoblotting.

### Agrobacterium Rhizogenes‐Mediated Transformation

Hairy root transformation in trifoliate orange was performed following a previously described protocol with minor modification.^[^ ^39^ ^]^ The coding sequences of *CtrSTP1*, *CtrBBX32*, *CtrZAT10*, and CtrCIPK6 (excluding stop codons) were individually cloned into the pK7WG2D vector, and the recombinant plasmids were introduced into *A. rhizogenes* strain MSU440 (Cat. AC1070; Weidi Biotechnology, Shanghai, China). Leaves of two‐month‐old trifoliate orange seedlings were removed, followed by excision of the roots. The basal parts of the stems were gently pricked with a syringe needle and then vacuum‐infiltrated (three 10‐min cycles) in the *A. rhizogenes* suspension (OD_600_ = 0.8) harboring the respective plasmids. The infiltrated stems were transferred to a humidity dome tray and cultivated for two months under high‐humidity dark conditions. GFP fluorescence of the roots was subsequently visualized using a handheld UV lamp (LUYOR‐3415RG, USA), and only plants with uniformly fluorescent roots were selected for hydroponic culture in Hoagland's solution for 30 days. Transcript levels of the respective genes were confirmed by RT‐qPCR using a small amount of positive roots, and the plants with overexpression of the examined genes were selected for further analysis.

### Yeast One‐Hybrid (Y1H) Assays

The 1‐kb promoter fragment of *CtrSTP1* was amplified by PCR and cloned into the bait vector pAbAi. The cDNA library screening was carried out according to the protocol in the Matchmaker Gold Yeast One‐Hybrid Library Screening System Kit (Cat. 630491; Clontech, Mountain View, CA, USA). Subsequently, DNA sequencing analysis of positive clones (Table S2, Supporting Information) was performed. The Y1H assay was used to verify the binding of CtrBBX32 and CtrZAT10 to the *CtrSTP1* promoter, the binding of CtrBBX32 to the *CtrZAT10* promoter, and the binding of CtrZAT10 to the *CtrBBX32* promoter. The promoters of *CtrSTP1*, *CtrZAT10*, *CtrBBX32*, and the truncated promoter fragments containing the expected *Cis*‐acting element were inserted into the pAbAi vector. The coding sequences of *CtrBBX32* and *CtrZAT10* were amplified and fused with pGADT7 vector to obtain the prey vector. The bait and prey were successively transformed into yeast Y1HGold strain (pGADT7 + bait plasmid as a negative control). Y1H assay was performed following the yeast maker yeast transformation system 2 user manual (Cat. 630439; Clontech, Mountain View, CA, USA).

### Transient Dual‐Luciferase Assays

The CDSs without the stop codon of *CtrBBX32*, *CtrBBX32^S108A^*, and *CtrZAT10* were respectively inserted into pK7WG2D vector with 3×Flag tag. The CDS without the stop codon of *CtrCBL1* was inserted into pGWB405 vector with GFP tag. The CDS without the stop codon of *CtrCIPK6* was inserted into pGWB417 vector with 3×MYC tag. The fusion vectors were used as effectors. The promoters of *CtrSTP1*, *CtrBBX32*, and *CtrZAT10* were cloned into pGreenII0800‐LUC vector as reporter.^[^ ^80^ ^]^ The reporter and effector constructs were transformed into *A. tumefaciens* GV3101 harboring the helper plasmid pSoup. Four‐week‐old *N. benthamiana* leaves were injected with the various reporter/effector combinations and then cultured in the growth chamber for 2–3 days.

The lowlight‐cooled CCD imaging apparatus (Night Shade LB985, Berthold, Bad Wildbad, Germany) was used to capture LUC images, in which d‐fluorescein was evenly applied to the surface of the leaves. LUC and REN activities were measured using a dual luciferase reporting kit (Promega, WI, USA). The relative intensity of the fluorescence signal was measured using the Infinity 200 Pro enzyme‐labeled apparatus (Infinity 200 Pro, Tecan, Switzerland). For immunoblot analysis, Anti‐DDDDK (Cat. AE063; ABclonal, Wuhan, China), Anti‐MYC (Cat. AE070; ABclonal, Wuhan, China), Anti‐GFP (Cat. AE078; ABclonal, Wuhan, China), and anti‐Tubulin Antibody (Cat. M20005S; ABmart, Shanghai, China) were utilized at a dilution of 1:5000. Three biological replicates were performed for the assay of each sample.

### Electrophoretic Mobility Shift Assays

CDSs of *CtrBBX32* and *CtrZAT10* without stop codons were respectively inserted into pGEX6P‐1 vector containing GST tag and finally transformed into *Escherichia coli* strain (DE3). The recombinant proteins of GST‐CtrBBX32 and GST‐CtrZAT10 were induced with 0.5 mm isopropyl β‐d‐1‐thiogalactoside under 16 °C for 16 h, and then purified by glutathione agarose resin (Cat. P2258; Beyotime, Shanghai, China). The biotin‐labeled probe and mutation probe, as well as the biotin‐free competing probe were synthesized by Tsingke Biotechnology (Beijing, China). EMSA was performed using a chemiluminescent EMSA kit (GS009, Beyotime, Shanghai, China). The protein–DNA complex was separated on a 6% native polyacrylamide gel, then electroblotted onto a positively charged Hybond‐N + nylon membrane, followed by UV crosslink. The signal was detected by chemiluminescence imaging (Tanon 5200, Shanghai, China).

### Chromatin Immunoprecipitation (ChIP)‐qPCR Assays

The CDSs without stop codon of *CtrBBX32* and *CtrZAT10* were cloned into a pK7WG2D vector with CaMV 35S promoter driver 3×Flag tag to generate 35S:CtrBBX32‐Flag and 35S:CtrZAT10‐Flag constructs. The fusion constructs and empty vector were respectively transformed into *A. tumefaciens* GV3101 for the infection of the sweet orange callus. According to previous methods,^[^ ^81^, ^82^ ^]^ each of 1 g callus samples with the overexpression of *CtrBBX32*, *CtrZAT10* or the control callus were soaked in 1% formaldehyde solution for 20 min. After that, the cells were lysed, and the chromosomes were randomly separated and broken into fragments of a certain size through ultrasonic treatment. The extracted protein/chromatin complex was precipitated with antiagarose antibody DDDDK magnetic agarose beads (Cat. M185‐10RMBL; MBL, Nagoya, Japan). DNA fragments are released by reverse crosslinking and protein digestion. Specific primers were designed according to *CtrSTP1* promoter sequence, and the DNA products were analyzed by qPCR.

### Cold Treatments of Transgenic Plants and Physiological Measurements and Histochemical Staining

The VIGS lines and the TRV‐EV control of trifoliate orange were subjected to cold treatment at −6 °C for 6 h, followed by growth recovery at room temperature for 12 h. The overexpression or empty vector plants with hairy roots were exposed to −6 °C for 4–6 h, followed by recovery at room temperature for 12 h. As for lemon, the transgenic or wild type plants were treated at −2 °C for 6 h, followed by recovery at room temperature for 12 h. Plant phenotypes and chlorophyll fluorescence imaging were scored before cold treatment (before stress) and after growth recovery (after stress), while leaves and roots were sampled at the beginning and the end of cold treatment for following analyses. Chlorophyll fluorescence imaging was performed by IMAGINGPAM chlorophyll fluorimeter (Walz, Germany). EL was measured as previously described.^[^ ^83^ ^]^ MDA was extracted from 0.1 g of leaf powder with 1 mL of 0.1 mm phosphate buffer solution (pH 7.8), and the content was assessed using an analytical kit (Cat. A003‐1‐2; Nanjing Jiancheng Bioengineering Institute, Nanjing, China) according to the manufacturer's instruction, while total protein concentrations were determined using an analytical kit (Cat. A045‐2‐2; Nanjing Jiancheng Bioengineering Institute, Nanjing, China). For the histochemical staining of H_2_O_2_ and O_2_ **^.−^** in the tissues, 3,3′‐diaminobenzidine (DAB) and NBT were used, respectively. Root dehydrogenase activity, an indicator of root viability,^[^ ^84^, ^85^, ^86^ ^]^ was examined using the triphenyl tetrazolium chloride method with an analysis kit (Cat. TP1023; Leagene, Beijing, China) following the manufacturer's protocol.

### Extraction and Quantification of Soluble Sugars

Plant tissues (0.1 g) were sampled in liquid nitrogen. As previously described,^[^ ^78^ ^]^ the soluble sugars were extracted in 75% (v/v) methanol, and 0.12 mg of ribitol was added to each sample as an internal standard. The 200 µL volume of each sample was dried and then derivatized with methoxyamine hydrochloride and *N*‐methyl‐*N*‐trimethylsilyl‐trifluoroacetamide in turn. A FULI GC9720 Plus Gas Chromatography System equipped with a flame ionization detector and a nonpolar HP‐5(5%‐phenyl)‐methylpolysiloxane column (Fuli Instruments, Zhejiang, China) was used for sugar content analysis.

### Yeast Two‐Hybrid (Y2H) Assays

The bait vector pGBKT7‐CtrBBX32 and the library yeast strain were transformed into Y2HGold yeast strain. The two yeasts were screened by mating. The obtained monoclonal was subjected to DNA sequencing (Table S2, Supporting Information). The *CtrBBX32* and *CtrCIPK6* coding regions were cloned into the pGBKT7 vector to generate bait proteins. The constructed plasmid was transformed into yeast strain Y2HGold, and the self‐activation of the bait protein was detected on an SD/‐Trp/‐His/‐Ade/+X‐α‐gal culture plate. Then the coding sequences of *CtrCIPK6* and *CtrCBLs* were amplified and fused with pGADT7 vector to generate prey proteins. The bait protein and prey protein constructs were cotransformed into Y2HGold yeast and coated in SD/‐Trp/‐Leu culture plates for 2–3 days.

### Luciferase Complementation Imaging (LCI) Assays

The luciferase complementation imaging assay was carried out in accordance with the previously described protocol.^[^ ^87^ ^]^ The sequences of *CtrCBL1*, *CtrCBL2*, and *CtrBBX32* without stop codons were cloned into JW771 vector, and the full‐length CDS of *CtrCIPK6* and *CtrCIPK9* were cloned into JW772 vector, respectively. The obtained constructs were transformed into *A. tumefaciens* strain GV3101, and the bacterial liquid combination was infiltrated into tobacco leaves and cultured in growth chamber for 2–3 days. The lowlight‐cooled CCD imaging apparatus (Night Shade LB985, Berthold, Bad Wildbad, Germany) was used to capture LUC images, in which d‐fluorescein was evenly applied to the surface of the leaves.

### Bimolecular Fluorescence Complementation (BiFC) Assays

The CDSs of *CtrCBL1*, *CtrCBL2*, and *CtrBBX32* without stop codons were cloned into nYFP vector, and the CDSs of *CtrCIPK6* and *CtrCIPK9* without stop codon were cloned into cYFP vector. The constructs were introduced into *A. tumefaciens* GV3101 for infiltration into *N. benthamiana* leaves. The combinations of CtrCBL2‐nYFP + CtrCIPK6‐cYFP and CtrBBX32‐nYFP + CtrCIPK9‐cYFP were used as negative controls. After 2–3 days, YFP signals were observed by confocal laser scanning microscopy (Leica TCS SP8; Leica, Wetzlar; Germany) under an excitation wavelength of 514 nm.

### In Vitro Pull‐Down Assays

The same amount of GST, GST‐CtrBBX32, and His‐CtrCIPK6 protein, and the same amount of GST, GST‐CtrCIPK6, and His‐CtrCBL1 protein were incubated with glutathione agarose resin (Beyotime, China) in pull‐down buffer (10 mm Tris‐HCl pH 7.5, 100 mm NaCl, 1 mm mercaptoethanol, 1 mm EDTA, 10% glycerol (v/v), and 0.5% Triton X‐100 (v/v)) at 4 °C for 4–6 hours. The protein mixture was washed with pull‐down buffer for five times, and then eluted in the elution buffer (pull‐down buffer + 10 mm glutathione, pH 8.0). The eluted protein was boiled with SDS loading buffer for 10 min. Finally, immunodetection was performed with anti‐GST (Cat. AF0174; Beyotime, Shanghai, China), anti‐His (Cat. AF2870; Beyotime, Shanghai, China) antibodies at a dilution of 1:5000.

### Co‐Immunoprecipitation (Co‐IP) Assays

The coding sequence of *CtrBBX32* excluding the stop codon was inserted into the pGWB405 vector with the C‐terminal GFP tag. Similarly, the coding sequence of *CtrCIPK6* without the stop codon was cloned into the pGWB417 vector featuring a 4×MYC tag. The empty vector pGWB405 served as a negative control. The recombinant vectors were subsequently transformed into *A. tumefaciens* strain GV3101 and coinfiltrated into the leaves of 4‐week‐old tobacco plants. Three days later, total protein was extracted from the tobacco leaves using a lysis buffer composed of 50 mm Tris‐HCl (pH 7.5), 150 mm NaCl, 1 mm EDTA, 1% Triton X‐100 (v/v), and a protease inhibitor (Cat. P1045; Beyotime, Shanghai, China). Subsequently, the MYC or CtrCIPK6‐MYC fusion proteins were co‐immunoprecipitated using anti‐MYC Magnetic Beads (Cat. P2118; Beyotime, Shanghai, China). For immunoblot analysis, the anti‐GFP (Cat. AE078; ABclonal, Wuhan, China) or anti‐MYC (Cat. AE070; ABclonal, Wuhan, China) antibodies were applied at a dilution of 1:5000.

### Cell Free Degradation Assays

About 1 g of root was collected and put into a centrifuge tube containing 1 mL of degradation reaction solution, and the tube was placed on ice for 60 min. The degradation reaction solution contained 25 mm Tris‐HCl, 10 mm NaCl, 10 mm MgCl_2_, 4 mm PMSF, 10 mm ATP, and 5 mm DTT. After centrifugation at 4 °C for 20 min, the supernatant was taken for the extracted active protein. Then, 50 µL GST‐CtrBBX32 prokaryotic‐induced protein was added to 1 mL protein extract, incubated in a metal bath at 24 °C, and sampled at intervals. For the proteasome inhibitor experiment, 20 µm MG132 was added before 30 min of the experiment. For immunoblot analysis, anti‐GST (Cat. AF0174; Beyotime, Shanghai, China) or anti‐Tubulin Antibody (Cat. M20005S; ABmart, Shanghai, China) were applied at a dilution of 1:5000.

### In Vitro Phosphorylation Assays

For in vitro phosphorylation experiment, the CDSs of *CtrBBX32* and *CtrCIPK6* without the stop codons were ligated to the pGEX6P‐1 vector, and that of *CtrCBL1* was ligated to the pET‐30a vector. The fusion proteins were expressed and purified in *Rosetta* (DE3). GST‐CtrCIPK6 and His‐CtrCBL1‐His were incubated in the reaction buffer (25 mm Tris‐HCl pH 7.5, 10 mm MgCl_2_, 1 mm CaCl_2_, 1 mm DTT, 2 mm ATP) for 30 min, and then the substrate protein GST‐CtrBBX32 or GST‐CtrBBX32S108A was added for 30 min. λ protein phosphatase (Cat. P2316S; Beyotime, Shanghai, China) was used to remove the phosphate groups on the substrate protein. Finally, immunodetection was performed with anti‐pSer/pThr antibody (Cat. PM3801; ECM Biosciences; Versailles, USA), anti‐GST (Cat. AF0174; Beyotime, Shanghai, China) or anti‐His (Cat. AF2870; Beyotime, Shanghai, China) antibodies at a dilution of 1:5000.

### Phos‐Tag Mobility Shift Assays

Total protein was extracted from CtrCIPK6‐Flag citrus callus treated under ambient temperature or 4 °C. For detection of CtrCIPK6‐Flag protein and the Tubulin control, samples were separated using 10% (w/v) SDS‐polyacrylamide gel electrophoresis (SDS‐PAGE). Phosphorylated protein bands were resolved on 10% SDS‐PAGE gels supplemented with 25 µm Phos‐tag reagent (Cat. PA101; Vazyme, Nanjing, China) and 50 µm MnCl_2_. Following electrophoresis, gels were equilibrated in transfer buffer containing 10 mm EDTA with gentle agitation for 10 min (repeated 1–2 times), rinsed thoroughly, and subjected to Western blotting. CtrCIPK6‐Flag and its phosphorylated forms were probed using an anti‐Flag antibody (Cat. AE063; ABclonal, Wuhan, China). To verify phosphorylation specificity, selected samples were treated with λ protein phosphatase (Cat. P2316S; Beyotime, Shanghai, China) prior to analysis.

### Statistical Analysis

The experimental results obtained in this work were replicated at least three times. All data were processed using GraphPad Prism v10.0. Two‐tailed Student^’^s *t*‐test analysis of variance was used for statistical analysis, taking *P < 0.05, **P < 0.01, ***P < 0.001 as significant.

### Accession Numbers

Accession numbers for *CtrCBL1*, *CtrSTP1*, *CtrZAT10*, *CtrBBX32*, and *CtrCIPK6* are LC887592, LC887593, LC887594, LC887595, and LC887596, respectively, which have been deposited in DDBJ. Sequences of other genes in this article can be found in the database of CPBD (Citrus Pan‐genome to Breeding Database, http://citrus.hzau.edu.cn/): *CtrSPS1* (Pt1g023140), *CtrSPS2* (Pt1g000180), *CtrSPS3* (Pt9g011690), *CtrSPS4* (Pt3g020450), *CtrCIPK9* (Pt3g034250), *CtrCBL2* ( Pt1g016950), *CtrCBL3* (Pt7g013370), *CtrCBL4* (Pt3g016110), *CtrCBL5* (Pt3g003240), *CtrCBL6* (PtUn002410), *CtrCBL7* (Pt1g016950), *CtrCBL8* (Pt3g003250), *CtrCBL9* (Pt9g001080), *CtrCBL10* (Pt1g004100), *CtrCBL11* (Pt1g016550), *CtrCBL12* (Pt2g002880), *CtrCBL13* (Pt5g007640), *CtrCBL14* (Pt1g020070), *CtrCBL15* (Pt9g014980), and *CtrCBL16* (Pt7g007060).

## Conflict of Interest

The authors declare no conflict of interest.

## Author Contributions

X.S., C.L., and J.‐H.L. planned and designed the research. X.S. performed the experiments and analyzed the data, with the assistance of Z.Z., W.X., Y.Z., M.L., X.J., B.D., L.C., and M.W. X.S. wrote the manuscript draft. C.L. and J.‐H.L. finalized writing and revision of the manuscript. All authors have read and approved the final version of the manuscript.

## Supporting information



Supporting Information

Supporting DataFile

## Data Availability

The data that support the findings of this study are available from the corresponding author upon reasonable request.
