# Supplementary material for: The CtrCBL1/CtrCIPK6 Complex of Citrus Phosphorylates CtrBBX32 to Regulate CtrSTP1‐Mediated Sugar Accumulation and Cold Tolerance
Source: Adv Sci (Weinh). 2025 Sep 26;12(46):e08372. doi: 10.1002/advs.202508372 (PMC12697822; doi:10.1002/advs.202508372)
Supplement: Supplementary file 1 — Supporting Information [file ADVS-12-e08372-s001.docx]

Supporting information

**The CtrCBL1/CtrCIPK6 Complex of Citrus Phosphorylates CtrBBX32 to Regulate *CtrSTP1*-mediated Sugar Accumulation and Cold Tolerance**

Xiangming Shang, Zeqi Zhao, Wei Xiao, Yike Zeng, Mengdi Li, Xin Jiang, Bachar Dahro, Lele Chu, Min Wang, Chunlong Li, Ji-Hong Liu

**Figure S1.** Root-highly expression of *CtrSTP1* encodes a cold-inducible sugar transporter revealed by transcriptomic profiling.

**Figure S2.** Low-temperature treatment promoted the accumulation of soluble sugars in the roots of trifoliate orange.

**Figure S3.** The gene expression levels, photosynthetic rate and protein levels in the tested plants.

**Figure S4.** Hairy root-specific overexpression of *CtrSTP1* enhances plant cold tolerance in *Citrus trifoliata*.

**Figure S5.** Expression analysis of *CtrZAT10* and *CtrBBX32* in response to cold stress and across distinct plant tissues.

**Figure S6.** Subcellular localization and transcriptional activation activity of CtrBBX32 and CtrZAT10.

**Figure S****7.** CtrBBX32 and CtrZAT10 could not interact with each other.

**Figure S8.** CtrZAT10 fails to bind to the promoter of *CtrBBX32*.

**Figure S9.** Analysis of *cis*-acting elements in the promoter of *CtrZAT10*.

**Figure S10.** CtrBBX32 binds to the promoter of *CtrZAT10*.

**Figure S11.** The expression levels of *CtrBBX32*, *CtrSTP1*, and *CtrZAT10* in the transgenic plants overexpressing *CtrBBX32*.

**Figure S12.** The expression levels of *CtrZAT10, CtrSTP1,* and *CtrBBX32* in the transgenic plants overexpressing *CtrZAT10*.

**Figure S13.** Characterization of CtrBBX32 antibody specificity.

**Figure S14.** The expression pattern of *CtrCIPK6*.

**Figure S15.** Detection of interaction between CtrCIPK6 and CtrZAT10.

**Figure S16.** Identification of phosphorylated residues in CtrBBX32 by using LC-MS/MS analysis.

**Figure S17.** Examination of CtrCBLs-CtrCIPK6 interactions, transcriptional profiling of *CtrCBLs*, and subcellular localization of CtrCBL1.

**Figure S18.** The gene expression levels and protein levels in the tested plants.

**Table S1.** The list of candidates obtained from Y1H library screening.

**Table S2.** The list of candidates obtained from Y2H library screening.

**Table S3.** List of primers used in this study.


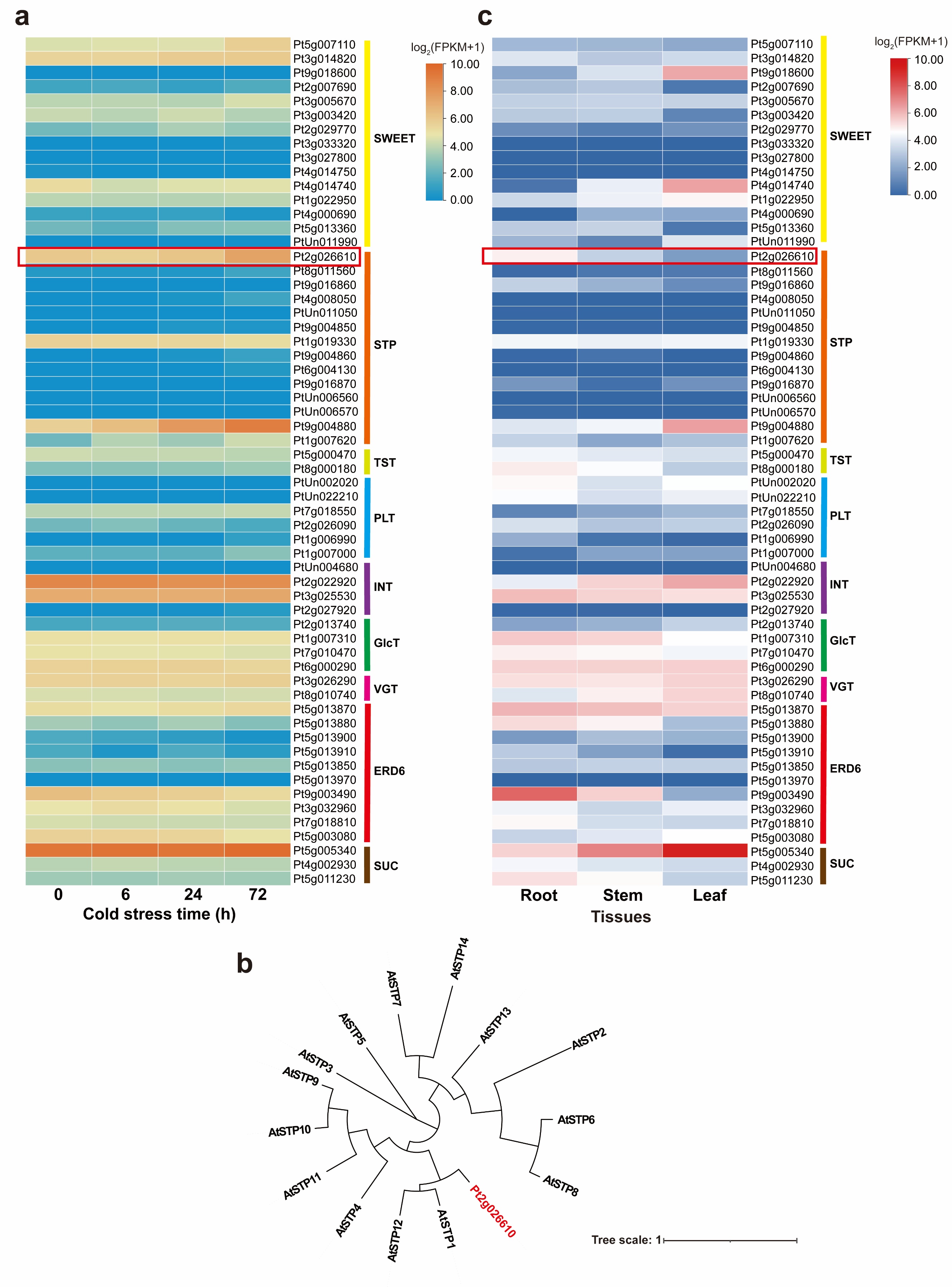


**Figure S1.** Root-highly expression of *CtrSTP1* encodes a cold-inducible sugar transporter revealed by transcriptomic profiling. a) Heatmap showing the expression levels of sugar transporters in trifoliate orange across various time points under 4°C treatment. b) The phylogenetic analysis of *Pt2g026610* (*CtrSTP1*) with *STP* members in *Arabidopsis*. c Heatmap showing the expression levels of sugar transporters in different plant tissues of trifoliate orange. In panels A and C, the indicated scale represents the log2-transformed values of gene expression levels.


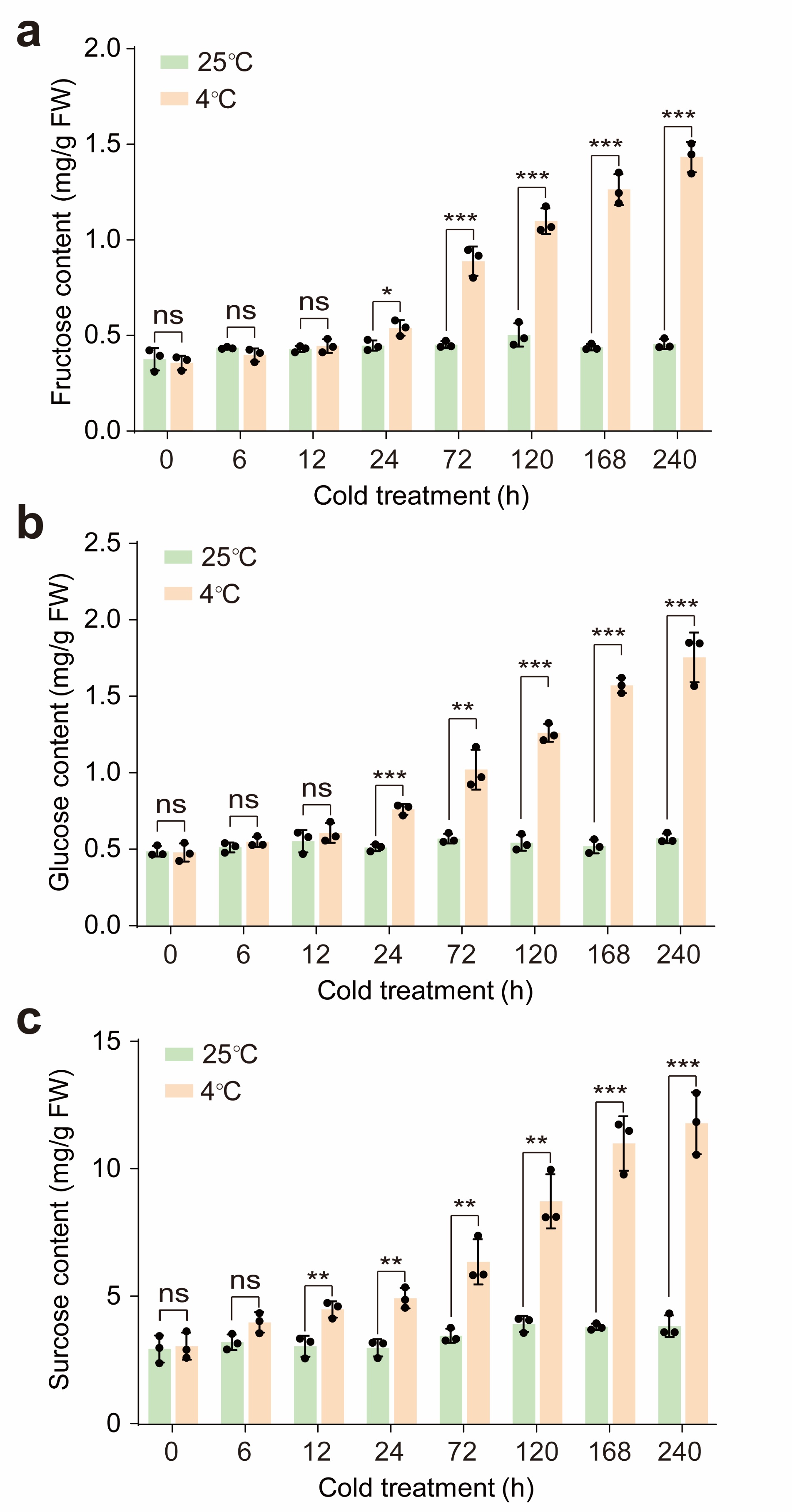


**Figure S2.** Low-temperature treatment promoted the accumulation of soluble sugars in the roots of trifoliate orange. a-c) The content of fructose (a), glucose (b), and sucrose (c) in the roots of two-month-old trifoliate orange seedlings treated at 4°C or 25°C. Error bars denote ± standard deviation (SD, n = 3). Two-tailed Student^’^s *t-test* was conducted for analyzing the significant difference (**P* < 0.05, ***P* < 0.01, ****P* < 0.001; *P* > 0.05, ns, no significance).

**
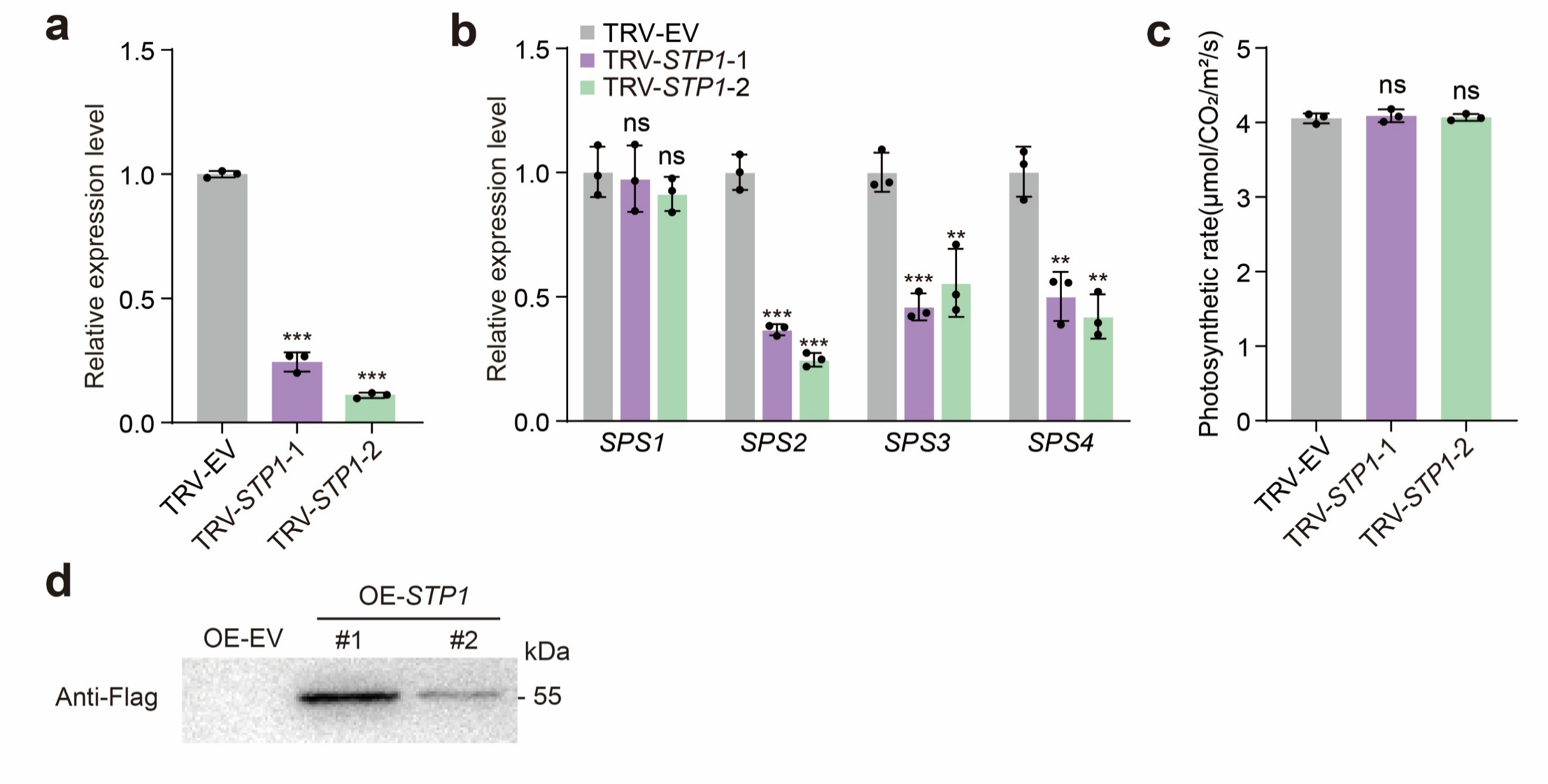
**

**Figure S3.** The gene expression levels, photosynthetic rate and protein levels in the tested plants. a) The relative expression of *CtrSTP1* in TRV-*CtrSTP1* lines was determined by RT-qPCR. The expression level of *CtrSTP1* in TRV-EV control was set to 1.0. b) The relative expression of *CtrSPS1*/*CtrSPS2*/*CtrSPS3*/*CtrSPS4* in TRV-*CtrSTP1* lines was determined by RT-qPCR. The expression level of target genes in TRV-EV control was set to 1.0. c) Photosynthetic rate in TRV-*CtrSTP1* lines. d) Western blot analysis was performed to determine the protein levels of CtrSTP1-Flag in transgenic lemon plants. Error bars denote ± standard deviation (SD, n = 3). Two-tailed Student^’^s *t-test* was conducted for analyzing the significant difference (***P* < 0.01, ****P* < 0.001; *P* > 0.05, ns, no significance).


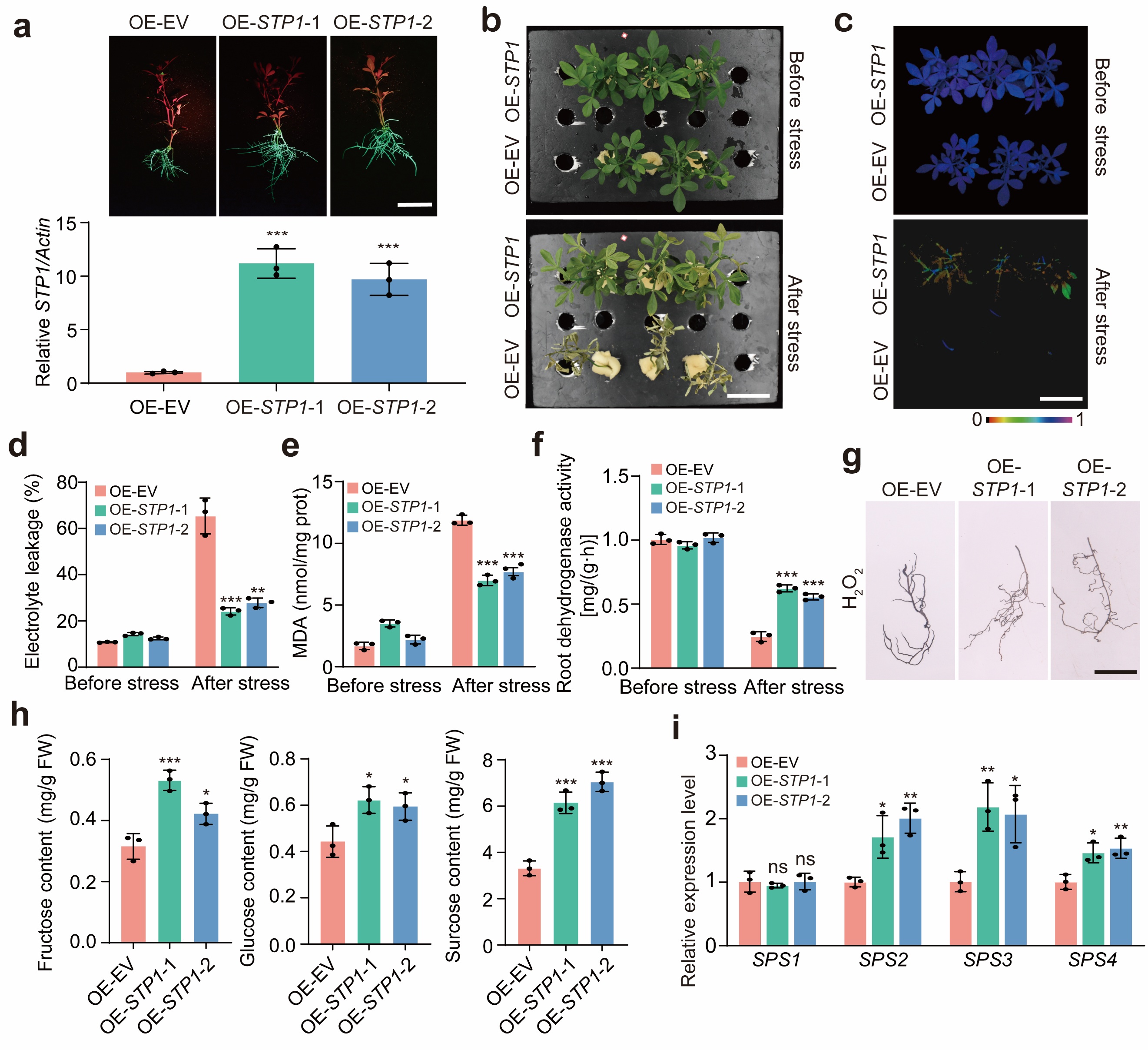


**Figure S4.** Hairy root-specific overexpression of *CtrSTP1* enhances plant cold tolerance in *Citrus trifoliata*. a) Observation of green fluorescent protein (GFP) signals (upper panel) and expression levels of *CtrSTP1* (lower panel) in the hairy roots of trifoliate orange plants expressing 35S:CtrSTP1-GFP (OE-*CtrSTP1*) or the empty vector (OE-EV). The photos shown in the figure labeled OE-EV, OE-*CtrSTP1*-1, and OE-*CtrSTP1*-2 are representative plants of each group containing three individual transgenic plants. Scale bar, 2 cm. The expression level of *CtrSTP1* in the root hair expressing the EV vector (OE-EV) was set to 1.0. b) Phenotypes of the plants containing hairy roots of OE-*CtrSTP1* and OE-EV before and after cold treatment at -4°C for 12 h, and then 12 h recovery at room temperature. Scale bars, 5 cm. c-f) Chlorophyll fluorescence imaging (c), electrolyte leakage (d), MDA content (e) and root activity (f) of the tested lines before and after the cold treatment. Scale bars, 5 cm. g) *In situ* detection of H_2_O_2_ in the roots of tested plants after the cold treatment. Scale bars, 2 cm. h) The contents of fructose, glucose, and sucrose in the hairy roots of the test lines. i) The relative expressions of *CtrSPS1/CtrSPS2/CtrSPS3/CtrSPS4* in the tested lines was determined by RT-qPCR. The expression level of target genes in OE-EV control was set to 1.0. Error bars denote ± standard deviation (SD, n = 3). Two-tailed Student^’^s *t-test* was conducted for analyzing the significant difference (**P* < 0.05, ***P* < 0.01, ****P* < 0.001; *P* > 0.05, ns, no significance).


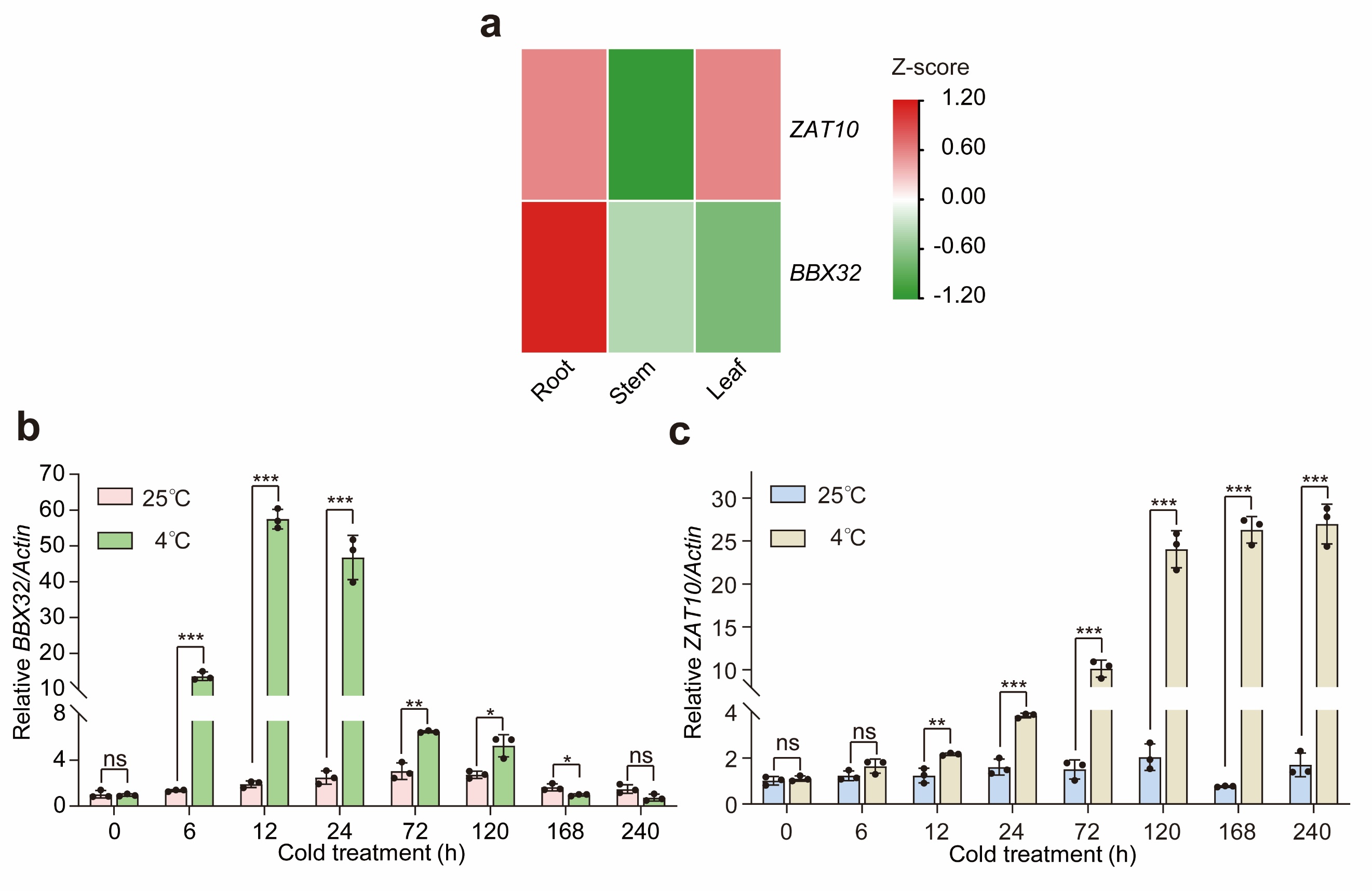


**Figure S5.** Expression analysis of *CtrZAT10* and *CtrBBX32* in response to cold stress and across distinct plant tissues. a) Heatmap showing the expression levels of *CtrZAT10* and *CtrBBX32* in the different tissues of trifoliate orange based on the transcriptome data. *CtrZAT10* was highly expressed in roots and leaves, while *CtrBBX32* was highly expressed in roots. The scale represents the line standardization of gene expression level. b,c) The relative expressions of *CtrBBX32* (b) and *CtrZAT10* (c) in trifoliate orange roots under normal conditions (25°C) or under cold treatment (4°C). The expression levels of *CtrBBX32* and *CtrZAT10* at 0 h under 25°C was set to 1.0. Error bars denote ± standard deviation (SD, n = 3). Two-tailed Student^’^s *t-test* was conducted for analyzing the significant difference (**P* < 0.05, ***P* < 0.01, ****P* < 0.001; *P* > 0.05, ns, no significance).


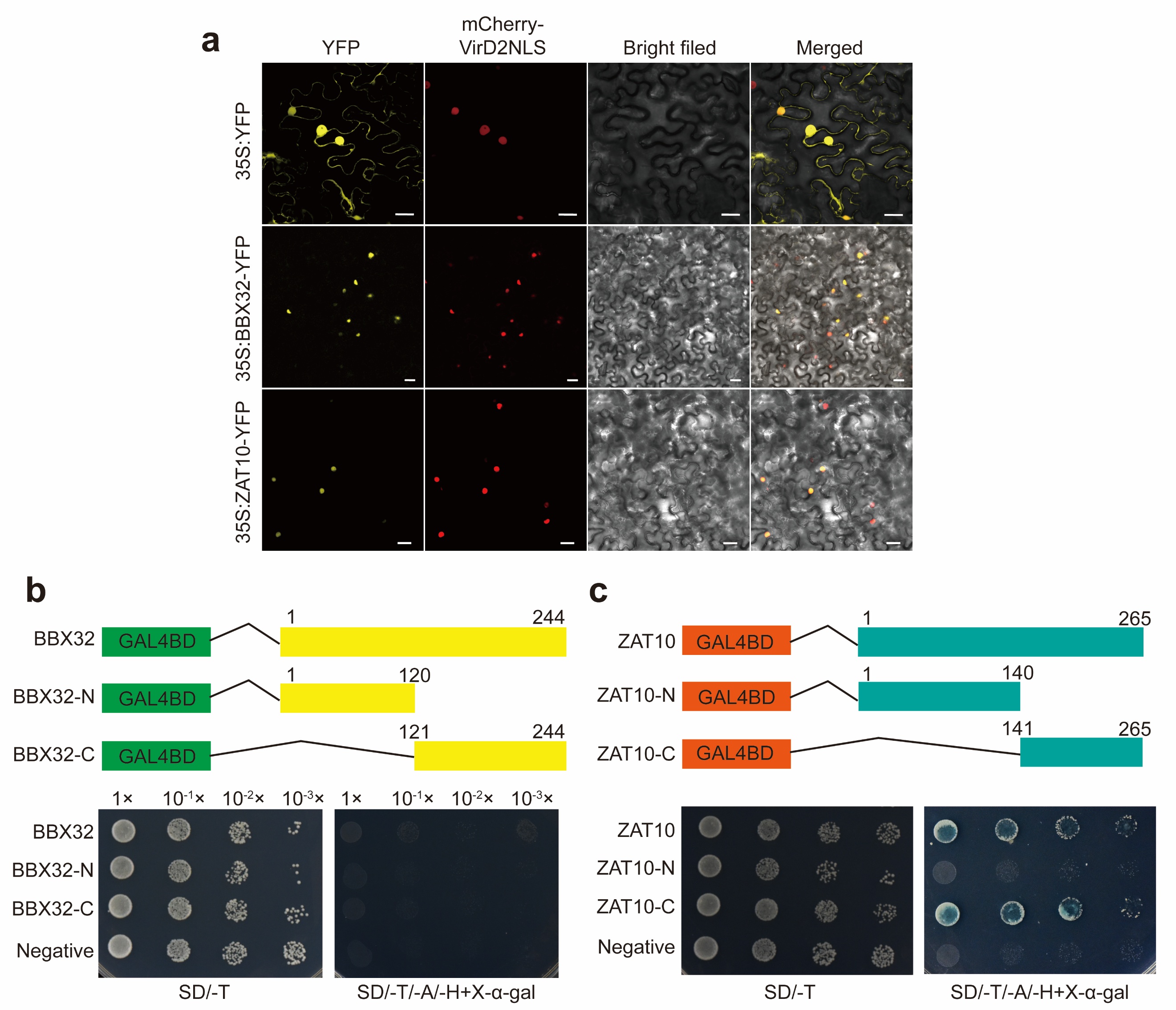


**Figure S6. Subcellular localization and transcriptional activation activity of CtrBBX32 and CtrZAT10.** a) The subcellular localization of CtrBBX32-YFP and CtrZAT10-YFP in *N.benthamiana* leaves, and VirD2NLS fused to mCherry as a nucleus marker. The results showed that CtrBBX32 and CtrZAT10 were expressed in the nucleus. Scale bars, 25 μm. b,c) Transcriptional activation analysis of CtrBBX32 and CtrZAT10. Full-length CtrBBX32 and CtrZAT10, as well as their truncated N-terminal (CtrBBX32-N, CtrZAT10-N) and C-terminal (CtrBBX32-C, CtrZAT10-C) fragments, were fused to the GAL4 DNA-binding domain of the pGBKT7 vector. The empty pGBKT7 vector (BD) was used as a negative control. The growth of the transformed yeast strain was assessed on SD/-Trp and SD/-Trp/-His/-Ade+X-α-gal medium, which were used to evaluate transcriptional activation activity.


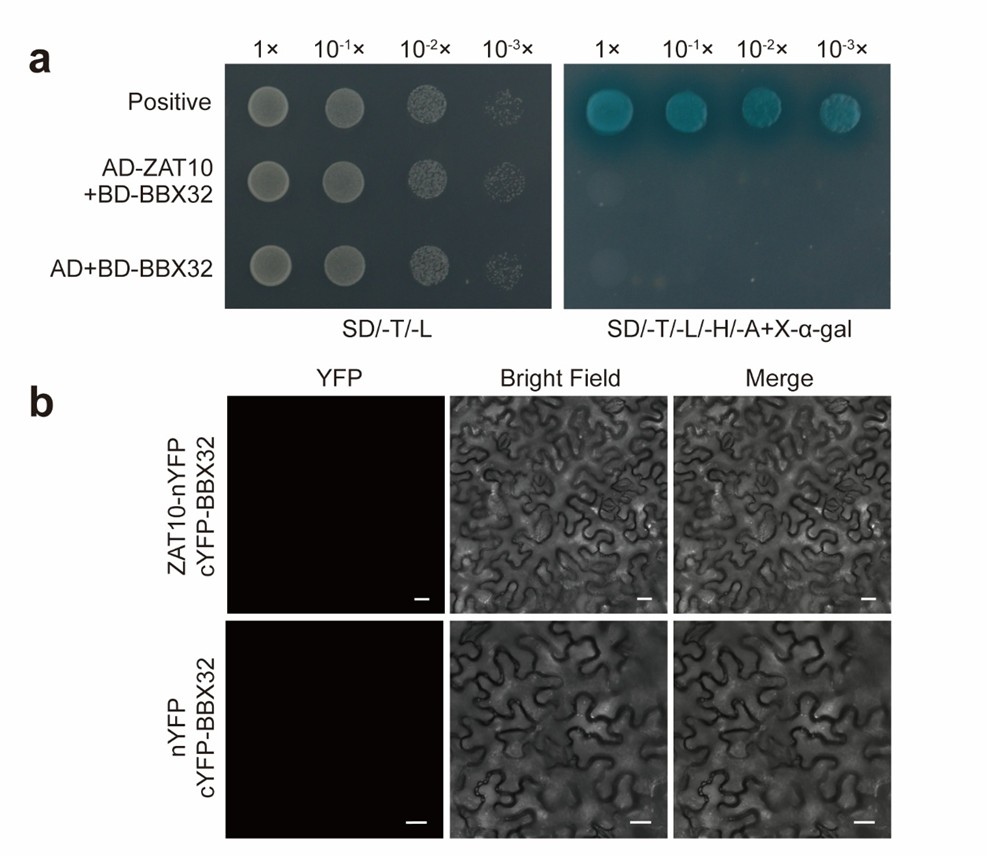


**Figure S7.** CtrBBX32 and CtrZAT10 could not interact with each other. a) The fusion vector pGBKT7-CtrBBX32 and pGADT7-CtrZAT10 were co-transformed into yeast strain and then grown on SD/-Trp/-Leu and SD/-Trp/-Leu/-His/-Ade/+X-α-gal medium. The positive control was pGBKT7-P53 +pGADT7-P53, and pGBKT7-CtrBBX32+pGADT7 empty vector was used as negative control. b) BiFC assay showed no interaction between CtrBBX32 and CtrZAT10. CtrBBX32 was fused with the C-terminus of yellow fluorescent protein (cYFP), while CtrZAT10 was fused with the N-terminal region of YFP (nYFP). Using nYFP + cYFP-CtrBBX32 as a negative control. Scale bars, 25 μm.


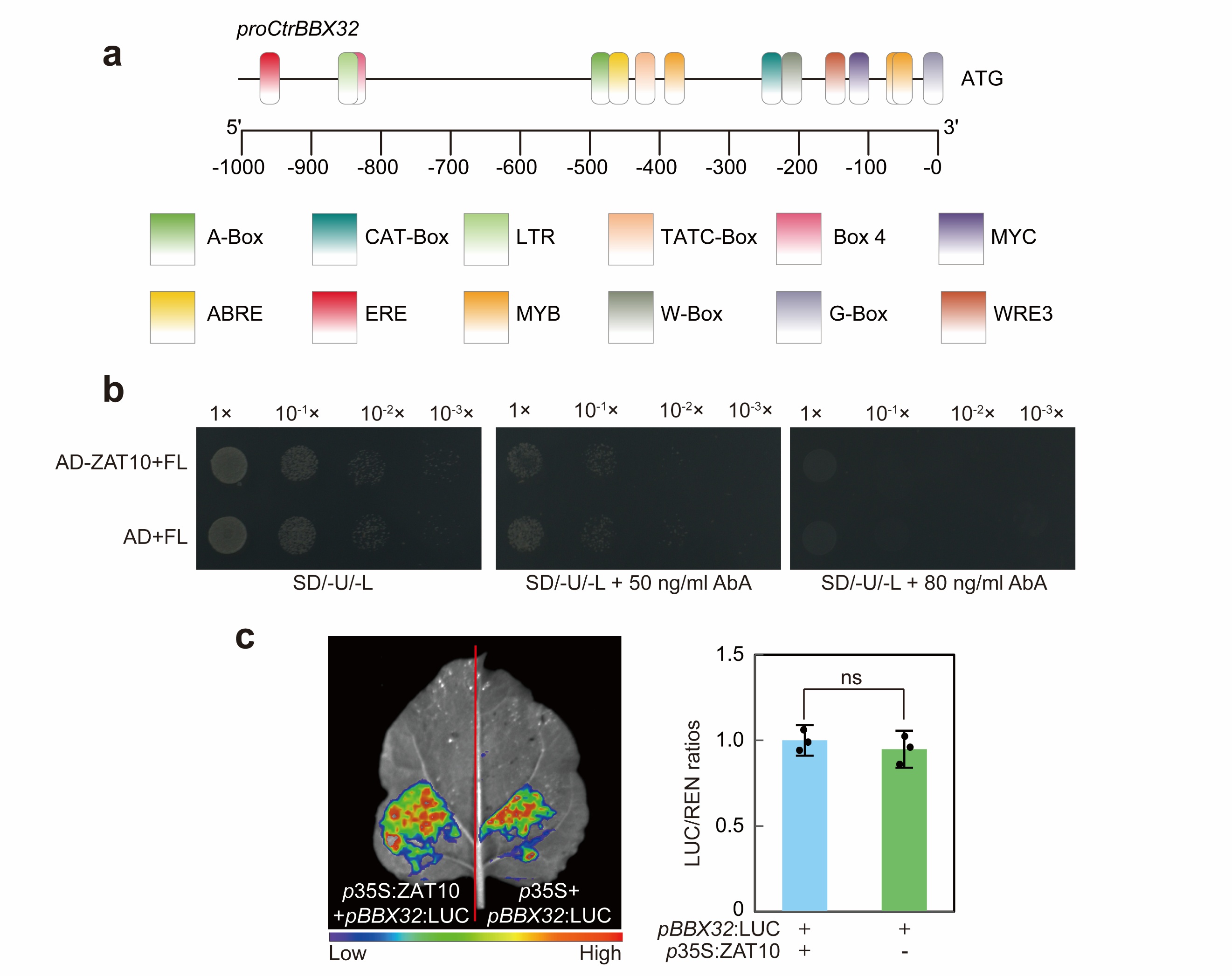


**Figure S8.** CtrZAT10 fails to bind to the promoter of *CtrBBX32*. a) Analysis of *cis*-acting elements of *CtrBBX32* promoter. b) The yeast strain transformed with prey (pGADT7-CtrZAT10) and bait (*CtrBBX32* promoter, FL), as well as the negative control (FL + pGADT7), grew on SD/–Ura/–Leu, SD/–Ura/–Leu/+AbA (50 ng/ml) and SD/–Ura/–Leu/+AbA (80 ng/ml) medium. c) The effect of CtrZAT10 on the LUC activity of *CtrBBX32* promoter was detected by dual luciferase assay. The left panel shows a representative bioluminescence image of the LUC signal from *N. benthamiana* leaves infiltrated with the indicated effectors and reporters. CtrZAT10 was driven by the 35S promoter as effectors. LUC was driven by the *CtrBBX32* promoter as a reporter. The relative LUC activity was shown on the right panel. Error bars denote ± standard deviation (SD, n = 3). Two-tailed Student^’^s *t-test* was conducted for analyzing the significant difference (ns, no significance, *P* > 0.05).


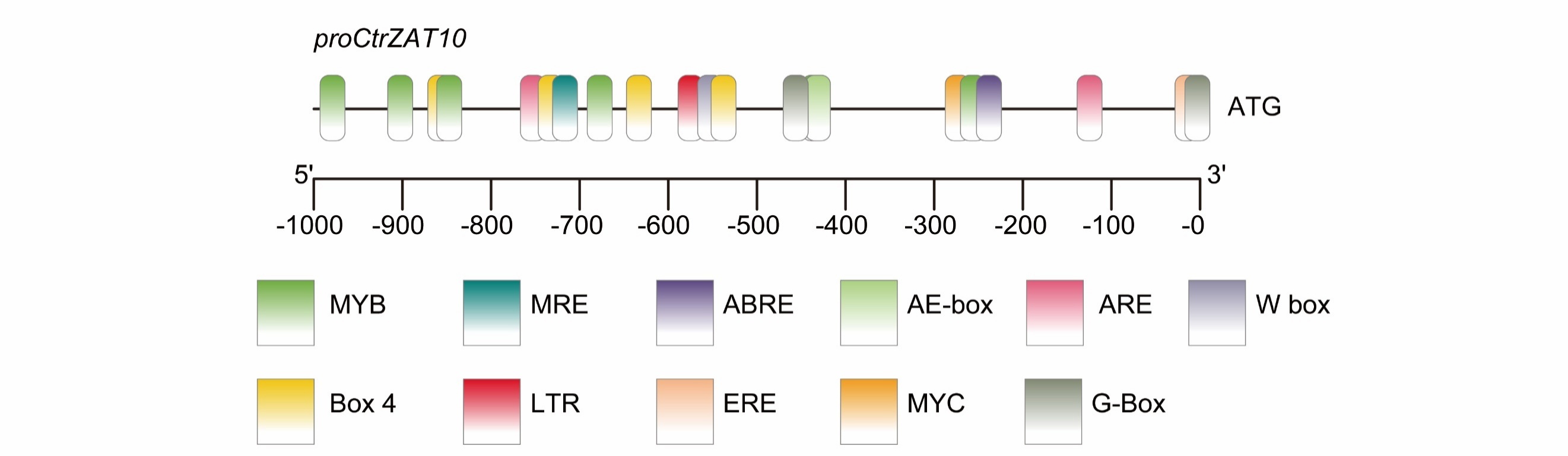


**Figure S9.** Analysis of *cis*-acting elements in the promoter of *CtrZAT10*.


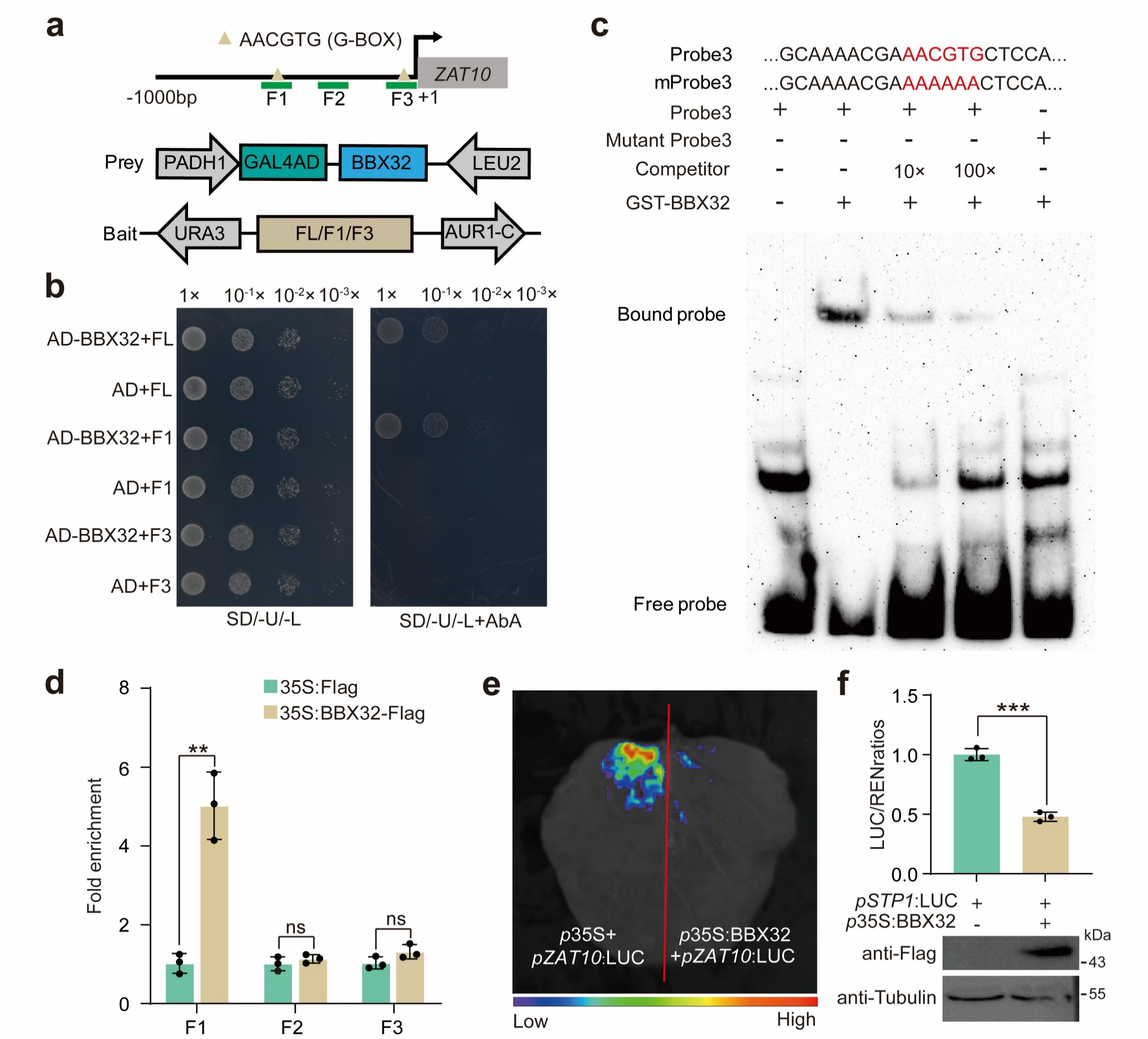


**Figure S10.** CtrBBX32 binds to the promoter of *CtrZAT10*. a) The *CtrZAT10* promoter schematic diagram for yeast one-hybrid experiment and ChIP-qPCR, as well as the bait and prey structure schematic diagram for yeast one-hybrid experiment. The yellow triangle represents the G-Box element in the promoter fragment (F1 and F3). b) Yeast one-hybrid assay showed that CtrBBX32 directly bound to promoter of CtrZAT10 at F1 fragment. The yeast strain transformed with prey (pGADT7-CtrBBX32) and bait (FL, F1 or F3), as well as the negative control (bait + pGADT7), then grown on SD/–Ura/–Leu and SD/–Ura/–Leu+AbA (150 ng/ml) medium. c) EMSA assay showed that GST-CtrBBX32 fusion protein bound to the promoter of *CtrZAT10*. The red parts in the promoter sequence represent the core binding motif. d) ChIP-qPCR assay of CtrBBX32 bound to the F1 fragments of the *CtrZAT10* promoter. e,f) CtrBBX32 inhibited the expression of *CtrZAT10*. The left panel is a representative bioluminescence image of the LUC signal of the *N.benthamiana* leaves infiltrated with the specified effectors and reporters (e). CtrBBX32 are driven by the 35S promoter as effectors. LUC was driven by the *CtrZAT10* promoter as a reporter. The relative LUC activity is shown on the right panel (f). Error bars denote ± standard deviation (SD, n = 3). Two-tailed Student^’^s *t-test* was conducted for analyzing the significant difference (***P* < 0.01, ****P* < 0.001; *P* > 0.05, ns, no significance,).

*
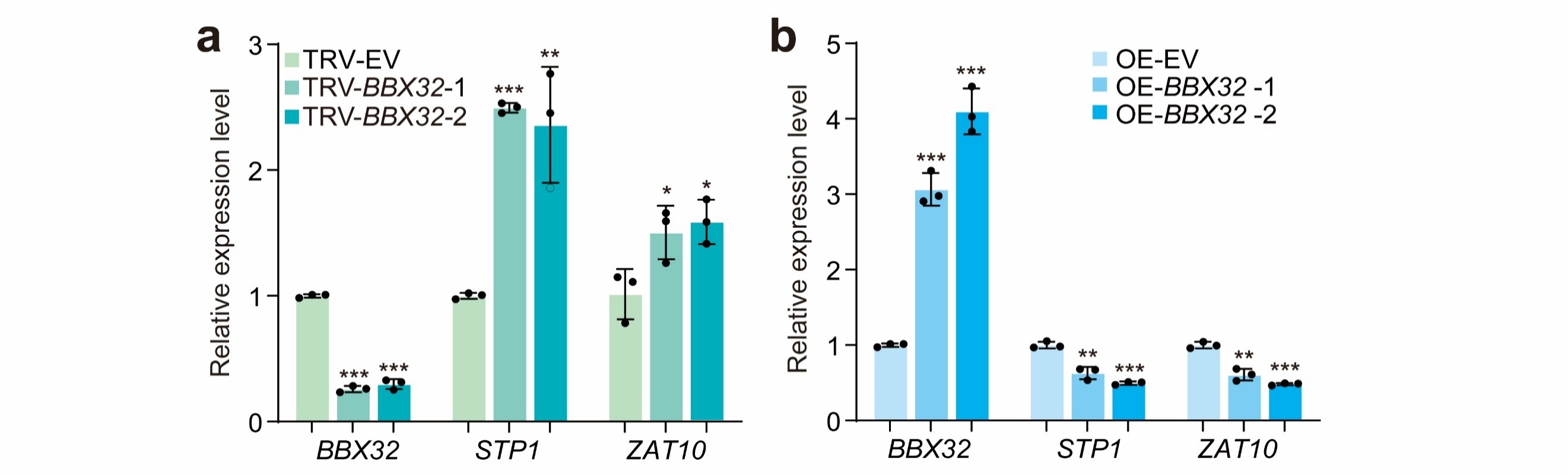
*

**Figure S11.** The expression levels of *CtrBBX32*/*CtrSTP1*/*CtrZAT10* in *CtrBBX32* transgenic plants. a,b) The relative expression of *CtrBBX32*/*CtrSTP1*/*CtrZAT10* in the tested lines was determined by RT-qPCR. The expression level of *CtrBBX32*/*CtrSTP1*/*CtrZAT10* in TRV-EV control and OE-EV were set to 1.0. Error bars denote ± standard deviation (SD, n = 3). Two-tailed Student^’^s *t-test* was conducted for analyzing the significant difference (**P* < 0.05, ***P* < 0.01, ****P* < 0.001).


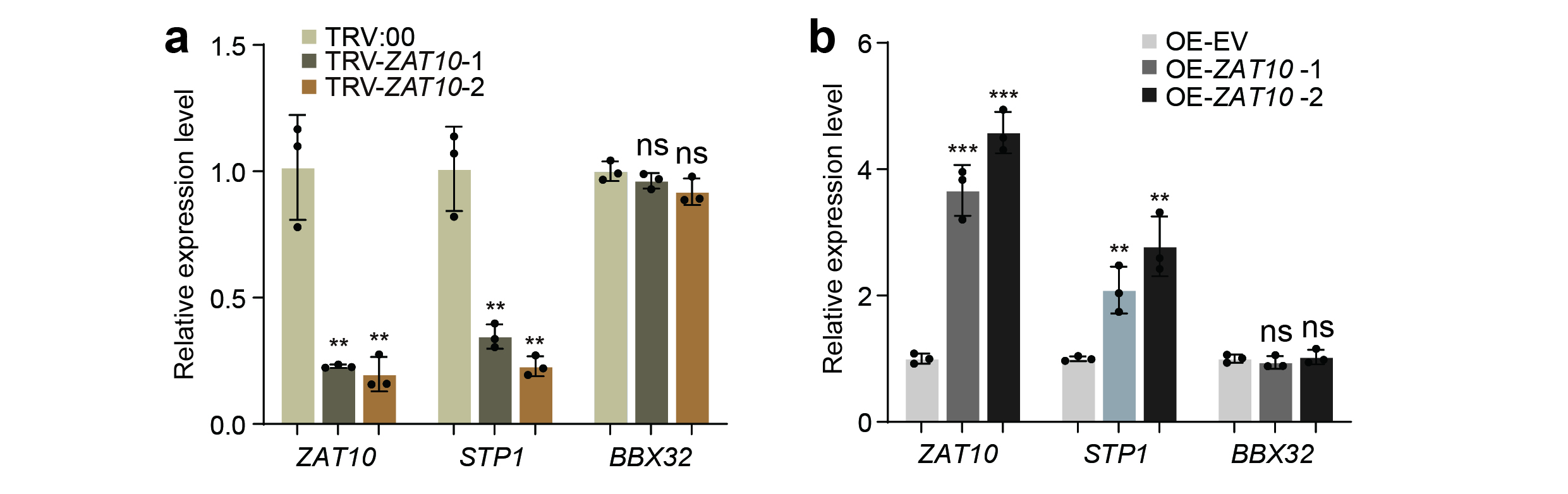


**Figure S12.** The expression levels of *CtrZAT10/CtrSTP1/CtrBBX32* in *CtrZAT10* transgenic plants. a,b) The relative expression of *CtrZAT10* */CtrSTP1/CtrBBX32* in the tested lines was determined by RT-qPCR. The expression level of *CtrZAT10/CtrSTP1/CtrBBX32* in TRV-EV control and OE-EV were set to 1.0. Error bars denote ± standard deviation (SD, n = 3). Two-tailed Student^’^s *t-test* was conducted for analyzing the significant difference (***P* < 0.01, ****P* < 0.001; *P* > 0.05, ns, no significance).


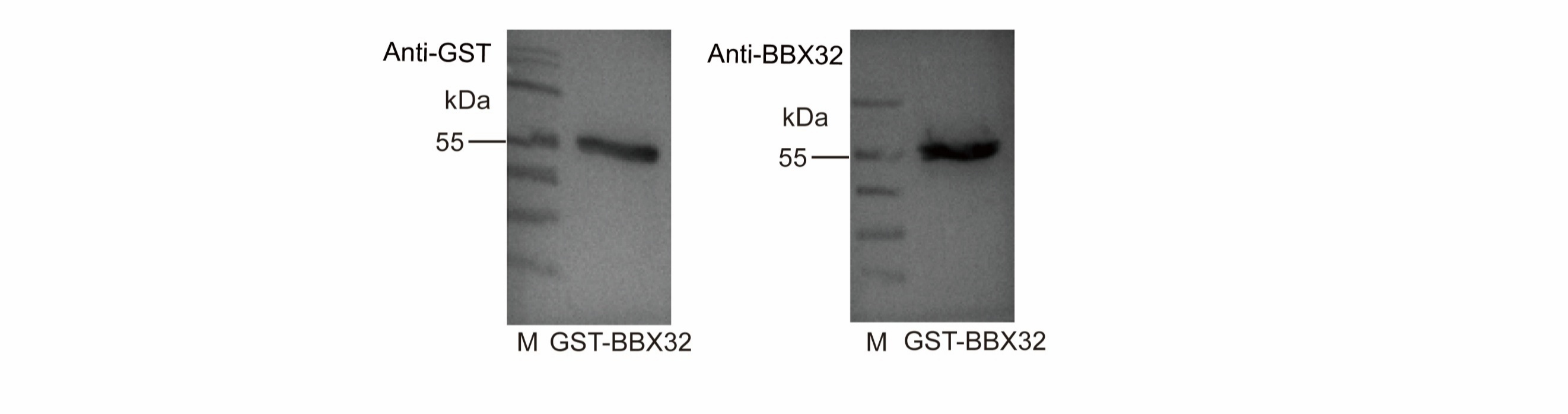


**Figure S13**. Characterization of CtrBBX32 antibody specificity. The left panel shows detection using an anti-GST antibody (diluted 1:5000), while the right panel shows detection with a specific CtrBBX32 antibody (diluted 1:5000).


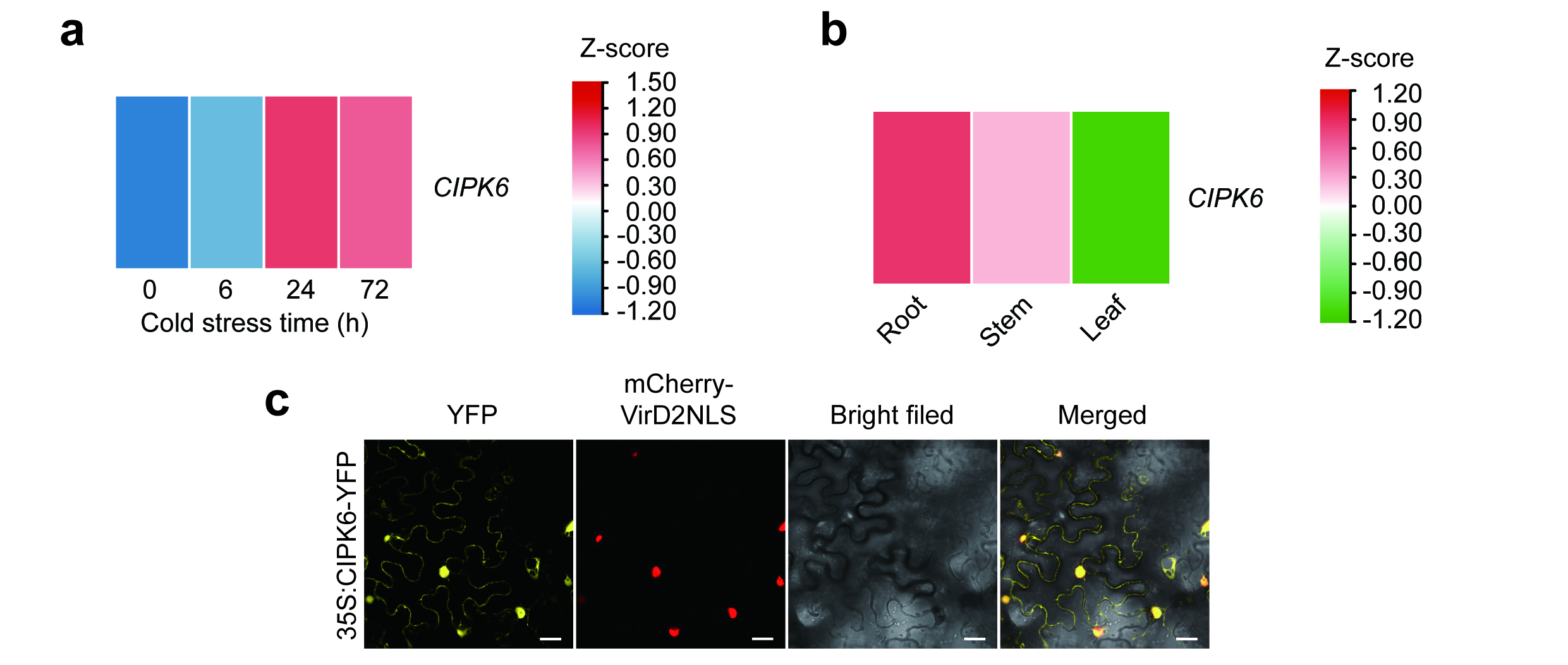


**Figure S14.** The expression pattern of *CtrCIPK6* and the subcellular localization of its encoded protein. a) Heatmap showed that *CtrCIPK6* was transcriptionally up-regulated by cold stress in the transcriptome data of trifoliate orange treated at low temperature. b) Heatmap showed that *CtrCIPK6* was highly expressed in roots in the transcriptome data of different tissues of trifoliate orange. The scale represents the line standardization of gene expression level in (a) and (b). c) The CtrCIPK6 protein was localized in the nucleus and cytoplasm. VirD2NLS fused to mCherry works as a nucleus marker. Scale bars, 25 μm.

**
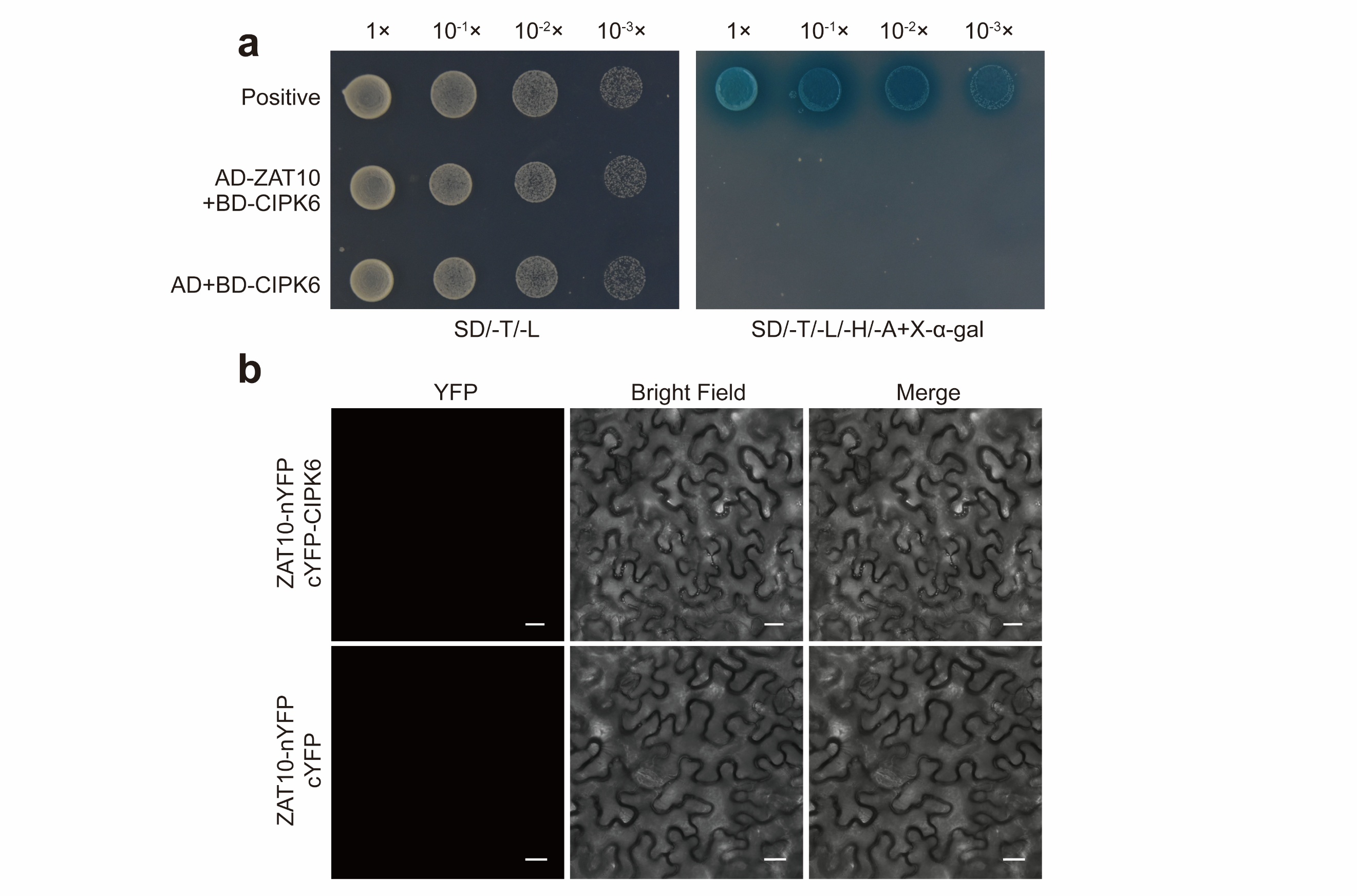
**

**Figure S15.** No interaction between CtrCIPK6 and CtrZAT10. a) Yeast cells transformed with the vectors (pGBKT7-CtrCIPK6 and pGADT7-CtrZAT10), along with the positive control (pGBKT7-p53 + pGADT7-p53) and negative control (pGBKT7-CtrCIPK6 + pGADT7), were plated on SD/-Trp/-Leu and SD/-Trp/-Leu /-His/-Ade/+X-α-gal medium. b) BiFC assay showed no interaction between CtrCIPK6 and CtrZAT10. CtrCIPK6 was fused with the C-terminus of yellow fluorescent protein (cYFP), while CtrZAT10 was fused with the N-terminal region of YFP (nYFP). Using CtrZAT10-nYFP + cYFP as a negative control. Scale bars, 25 μm.


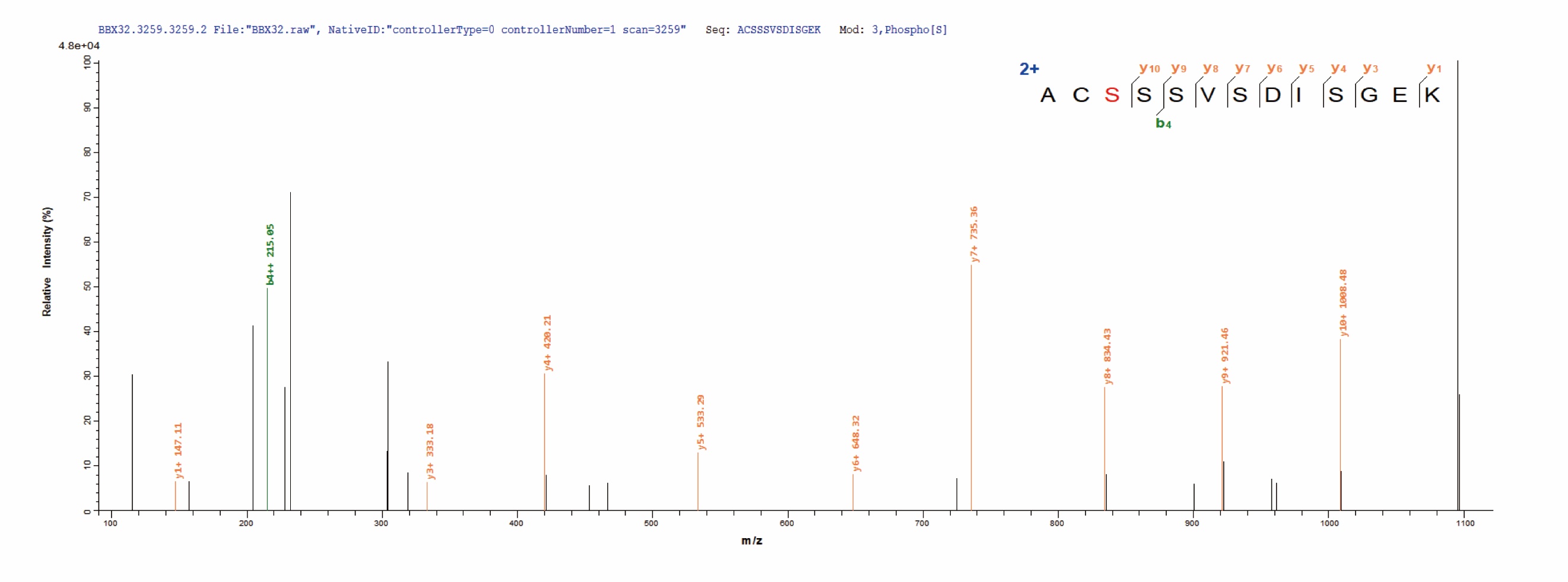


**Figure S16.** Phosphorylated residues in CtrBBX32 were identified using LC-MS/MS analysis. The notation "b" refers to N-terminal ions, while "y" indicates C-terminal ions of the peptide, both retaining their respective charges. The term "m/z" represents the mass-to-charge ratio.


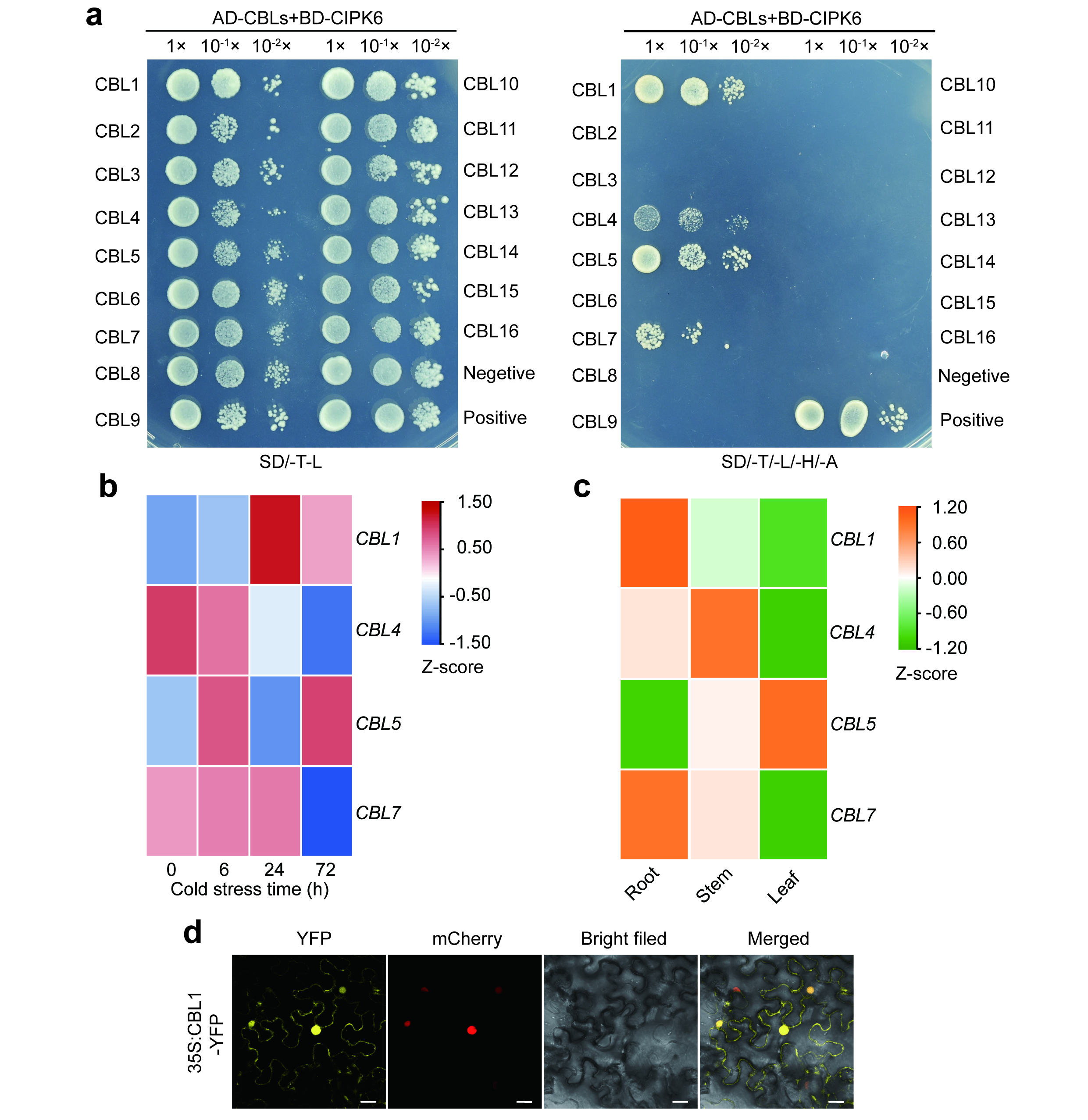


**Figure S17.** Examination of CtrCBLs-CtrCIPK6 interactions, transcriptional profiling of *CtrCBLs*, and subcellular localization of CtrCBL1. a) Y2H assay showed that CtrCIPK6 interacted with CtrCBL1/CtrCBL4/CtrCBL5/CtrCBL7. Yeast cells transformed with the vectors (pGBKT7-CtrCIPK6 + pGADT7-CtrCBLs), along with the positive control (pGBKT7-p53 + pGADT7-p53) and negative control (pGBKT7- CtrCIPK6 + pGADT7), were plated on SD/-Trp/-Leu and SD/-Trp/-Leu /-His/-Ade medium. b) The heat map results showed that the expression pattern of *CtrCBL1/CtrCBL4/CtrCBL5/CtrCBL7* in the transcriptome data of trifoliate orange treated at low temperature. c) The results of heat map showed the expression patterns of *CtrCBL1/CtrCBL4/CtrCBL5/CtrCBL7* in the transcriptome data of different tissues of trifoliate orange. The scale represents the line standardization of gene expression level in (b) and (c). d) The CtrCBL1 protein was localized in the nucleus and cytoplasm. VirD2NLS fused to mCherry as a nucleus marker. Scale bars, 25 μm.


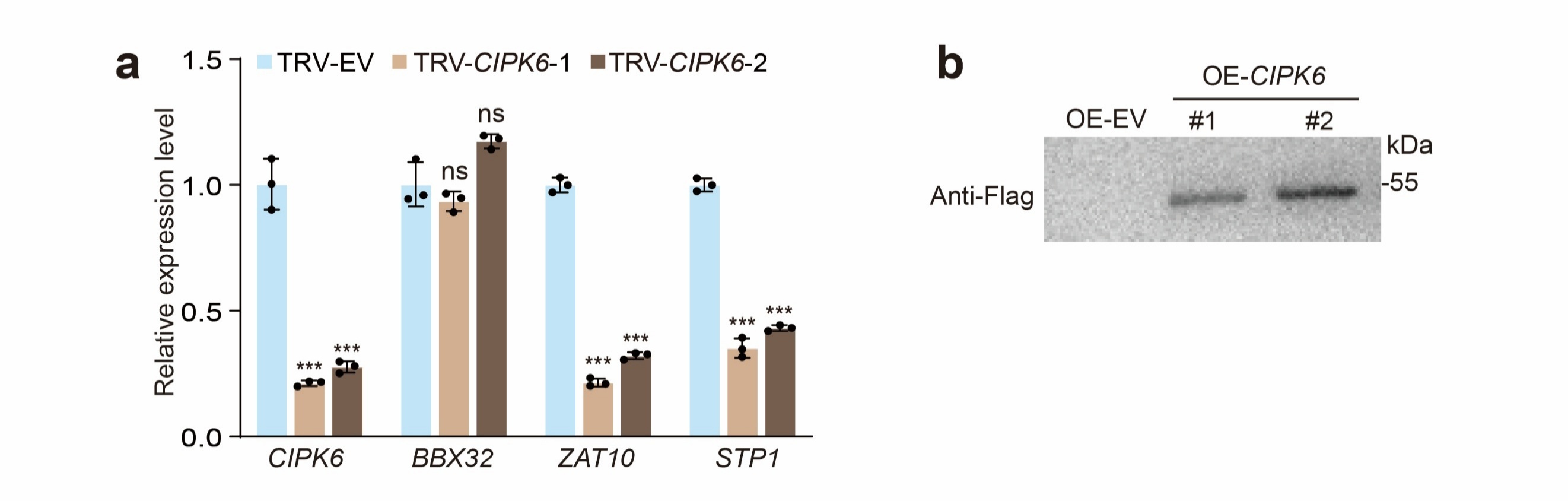


**Figure S18.** The gene expression levels and protein levels in the tested plants. a) Relative expression levels of *CtrCIPK6*/*CtrBBX32*/*CtrZAT10*/*CtrSTP1* in the TRV-*CtrCIPK6* lines. The expression level of *CtrCIPK6*/*CtrBBX32*/*CtrZAT10*/*CtrSTP1* in TRV-EV control was set to 1.0. Error bars denote ± standard deviation (SD, n = 3). Two-tailed Student^’^s *t-test* was conducted for analyzing the significant difference (****P* < 0.001; *P* > 0.05, ns, no significance). b) Western blot analysis was performed to determine the protein levels of CtrCIPK6-Flag in lemon plants.

**Table S1. The list of members obtained from Y1H library screening.**

**Table S2. The list of members obtained from Y2H library screening.**

**Table S3. List of primers used in this study.**
