# Supplementary material for: The CtrCBL1/CtrCIPK6 Complex of Citrus Phosphorylates CtrBBX32 to Regulate CtrSTP1‐Mediated Sugar Accumulation and Cold Tolerance
Source: Adv Sci (Weinh). 2025 Sep 26;12(46):e08372. doi: 10.1002/advs.202508372 (PMC12697822; doi:10.1002/advs.202508372)
Supplement: Supplementary file 2 — Supporting DataFile [file ADVS-12-e08372-s002.zip › 20250909 supplemental figures for blots.docx]

Original images


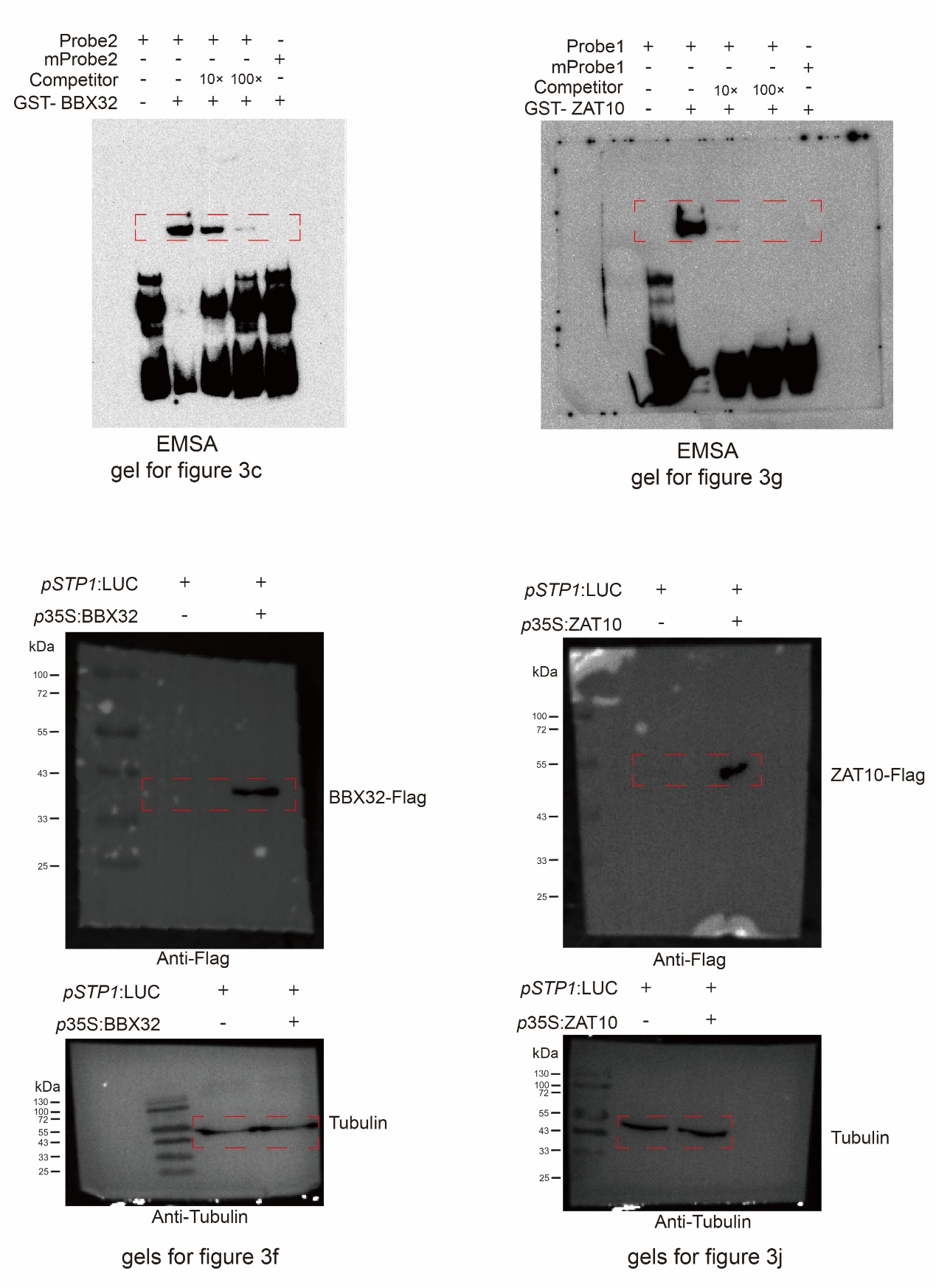


Original images for Figure 3.


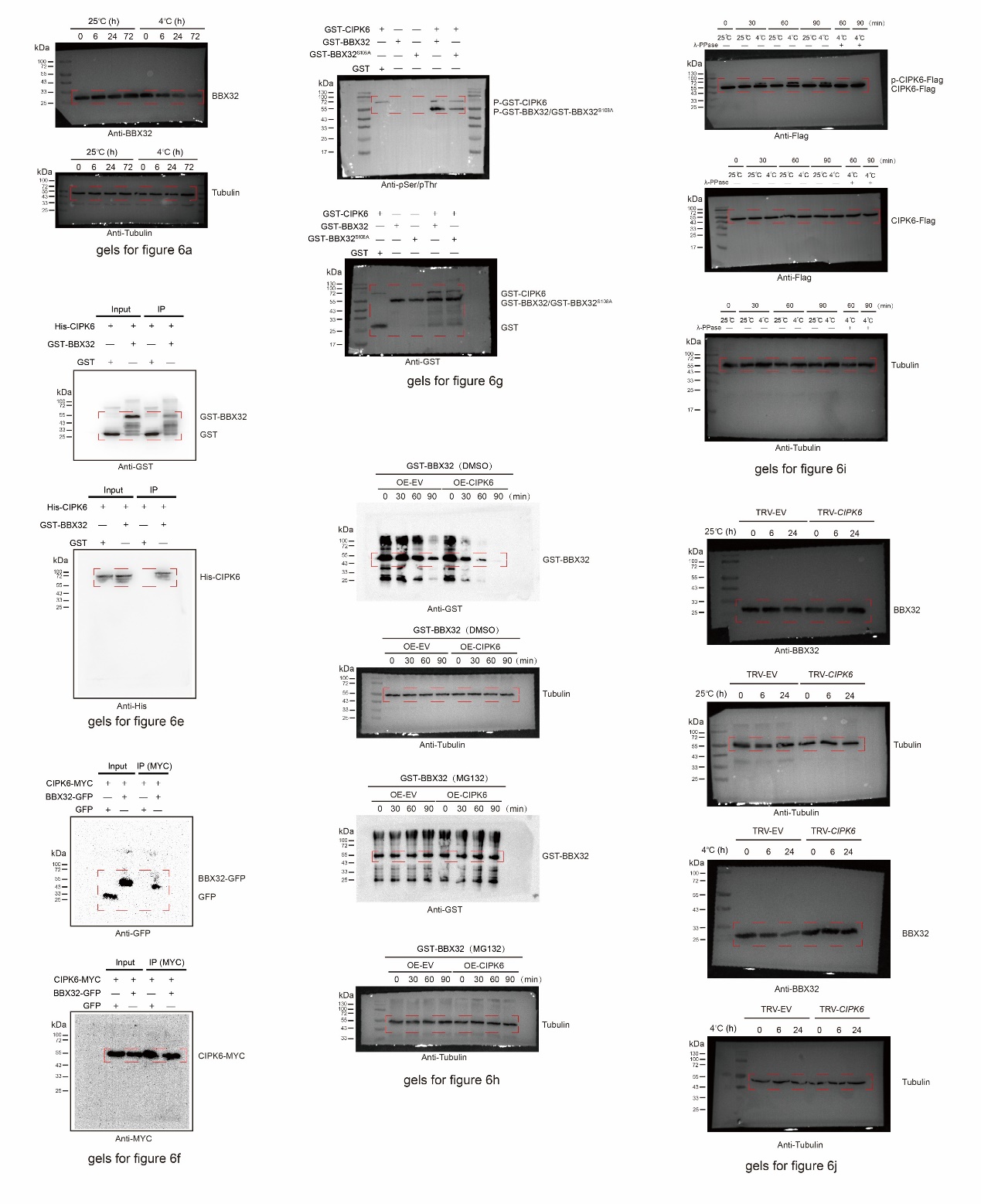


Original images for Figure 6.


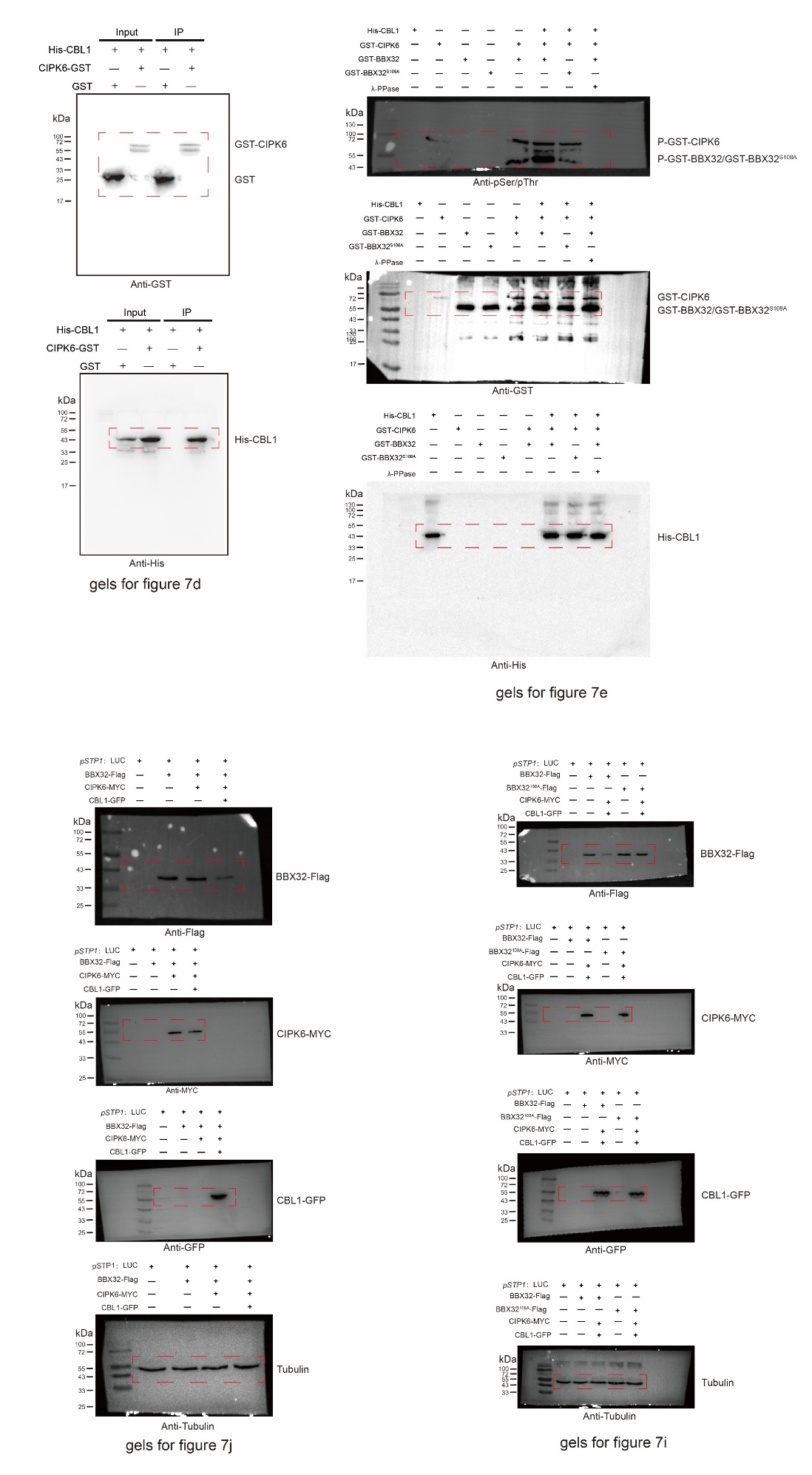


Original images for Figure 7.


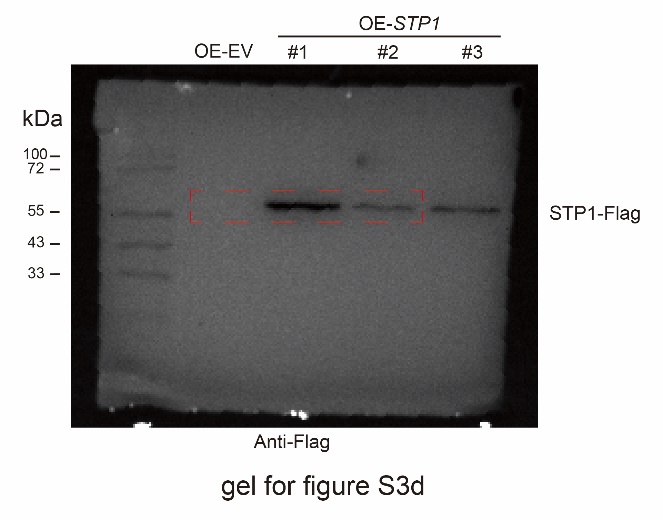


Original images for Figure S3.


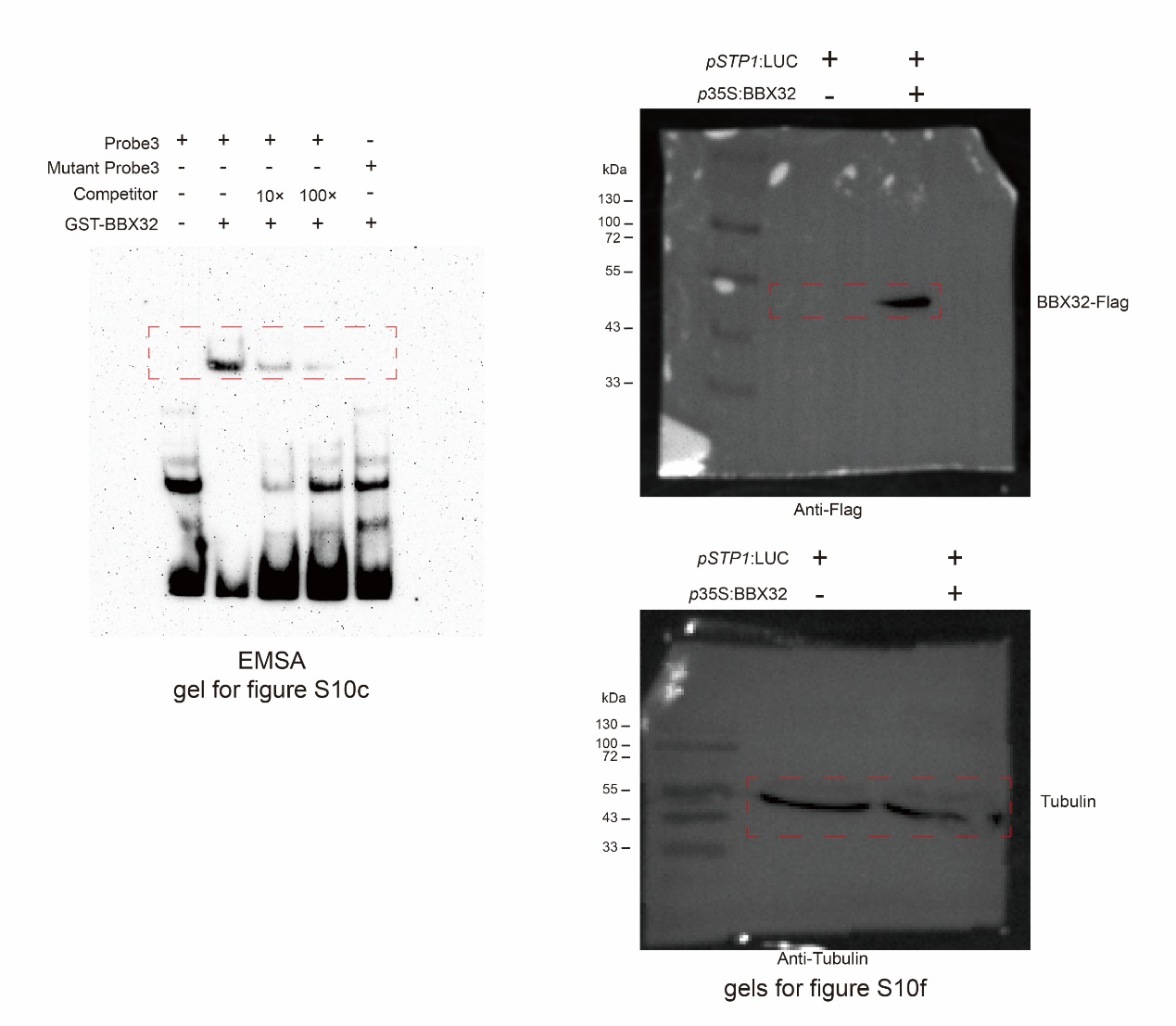


Original images for Figure S10.


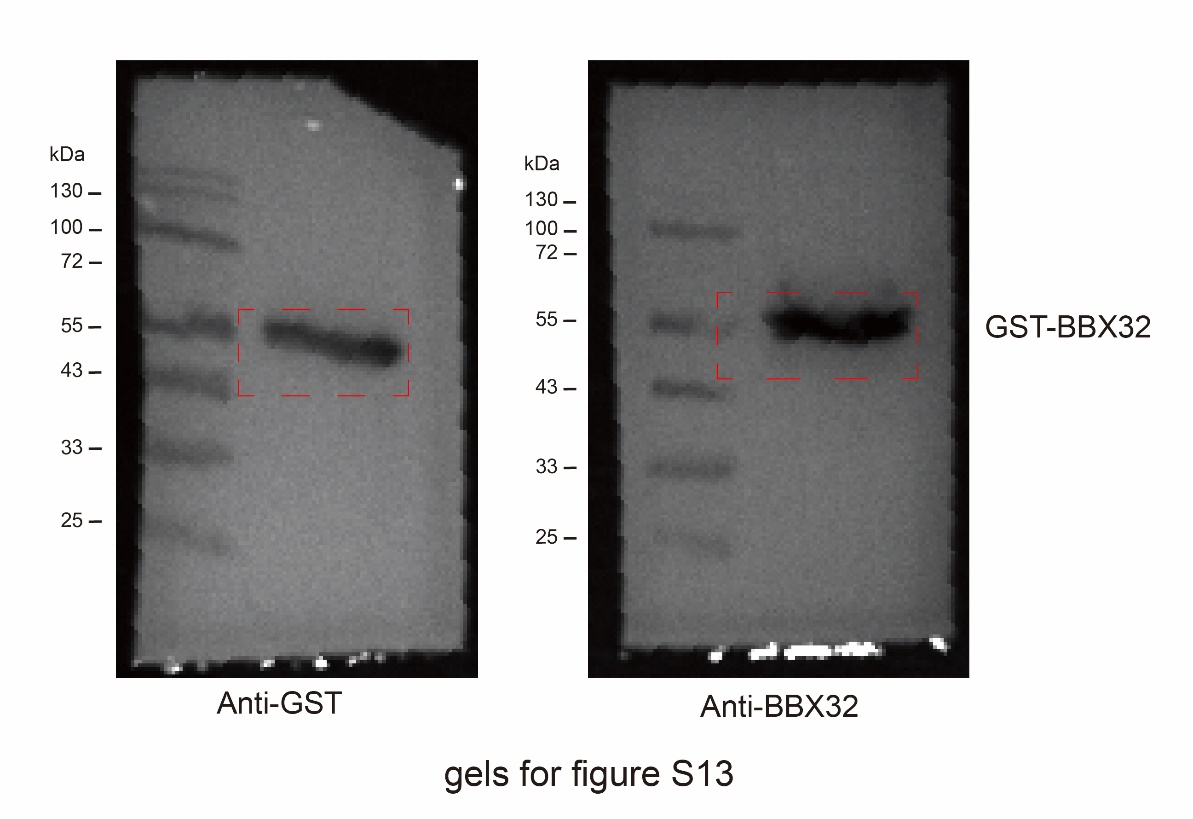


Original images for Figure S13.


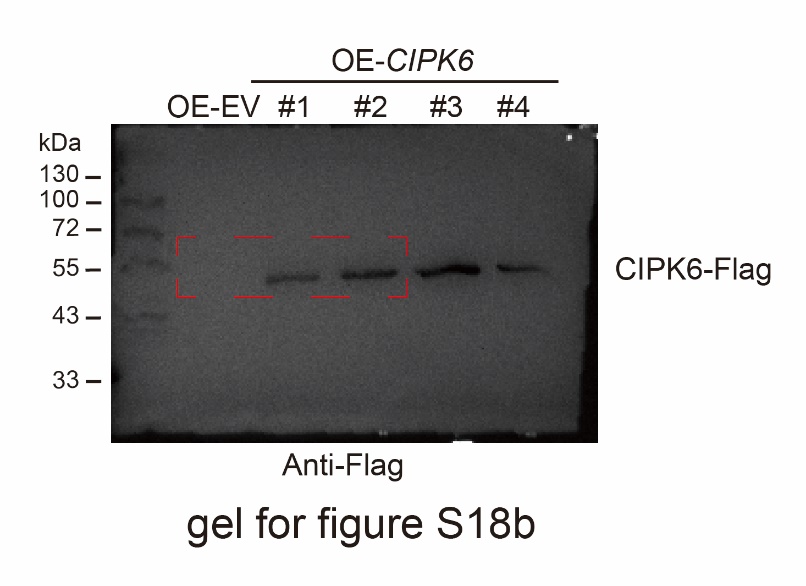


Original images for Figure S18.
